# Mapping the binding site topology of amyloid protein aggregates using multivalent ligands†

**DOI:** 10.1039/d1sc01263k

**Published:** 2021-06-07

**Authors:** Elena Sanna, Margarida Rodrigues, Steven G. Fagan, Timothy S. Chisholm, Klara Kulenkampff, David Klenerman, Maria Grazia Spillantini, Franklin I. Aigbirhio, Christopher A. Hunter

**Affiliations:** Department of Chemistry, University of Cambridge Lensfield Road Cambridge CB2 1EW UK herchelsmith.orgchem@ch.cam.ac.uk; Department of Clinical Neurosciences, Clifford Allbutt Building, University of Cambridge Cambridge CB2 0AH UK; Department of Clinical Neuroscience, Wolfson Brain Imaging Centre, University of Cambridge CB2 0QQ UK

## Abstract

A key process in the development of neurodegenerative diseases such as Alzheimer's and Parkinson's diseases is the aggregation of proteins to produce fibrillary aggregates with a cross β-sheet structure, amyloid. The development of reagents that can bind these aggregates with high affinity and selectivity has potential for early disease diagnosis. By linking two benzothiazole aniline (BTA) head groups with different length polyethylene glycol (PEG) spacers, fluorescent probes that bind amyloid fibrils with low nanomolar affinity have been obtained. Dissociation constants measured for interaction with Aβ, α-synuclein and tau fibrils show that the length of the linker determines binding affinity and selectivity. These compounds were successfully used to image α-synuclein aggregates *in vitro* and in the post-mortem brain tissue of patients with Parkinson's disease. The results demonstrate that multivalent ligands offer a powerful approach to obtain high affinity, selective reagents to bind the fibrillary aggregates that form in neurodegenerative disease.

## Abstract 2

Multivalent ligands offer a powerful approach to obtain high affinity reagents to bind the aggregates that form in neurodegenerative disease. Selectivity for different proteins was achieved by using different linkers to connect the head groups.

## Graphical abstract

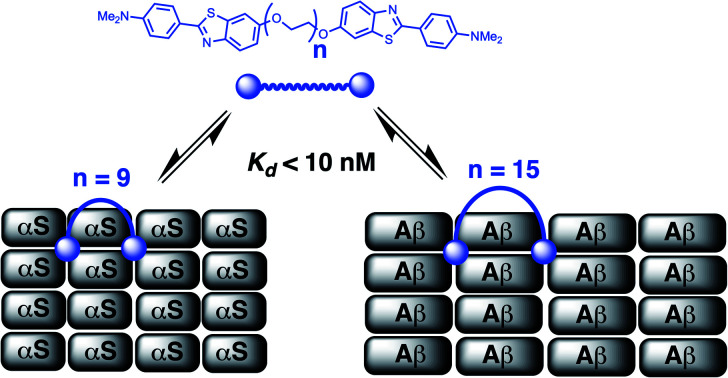

## Introduction

Neurodegenerative disorders like Alzheimer's and Parkinson's diseases are a growing medical problem in modern society.^1^ Although the molecular mechanism that leads to a propagation of these diseases is not well-understood, it has been established that the misfolding and aggregation of proteins such as β-amyloid and tau in Alzheimer's disease and α-synuclein in Parkinson's disease play a key role in the initiation and spread of the disease. Aggregation ultimately results in the deposition of insoluble fibrillary aggregates, which have a characteristic cross β-sheet structure, amyloids.^2,3^ The resulting fibrils are large in size, low in solubility, and are not crystalline. However, the smaller soluble aggregates formed in this process appear to be the toxic species that damage by different mechanisms and ultimately kill neurons. Recently using super-resolution imaging and atomic force microscopy, β-amyloid aggregates ranging in size from 20–200 nm were observed in the cerebrospinal fluid of patients with Alzheimer's disease, and the development of protofibrillar aggregates about 100 nm in length was found to cause increased inflammation.^4^ Thus soluble fibrillary aggregates play an important role in the development of Alzheimer's disease. However, currently there are a limited number of approaches to detect and characterise these soluble aggregates that are typically present in biofluids at low picomolar concentrations.^5^ The main approach is based on the use of antibodies that either target the post-translationally modified protein present in aggregates to make them aggregate selective or are based on sandwiching the aggregate with two antibodies that bind the same epitope on the protein.^5^ Small molecules such as thioflavin T have been developed, but the affinity and selectivity that can be achieved with these compounds is limited. Here we describe an alternative strategy where two head groups, which have a micromolar affinity for fibrillary aggregates, are linked by a flexible linker to obtain nanomolar dissociation constants. We show how variation of the linker length can be used to optimise the affinity and selectivity of these constructs for the fibrillar aggregates of Aβ, tau and α-synuclein.

**Fig. 1:**
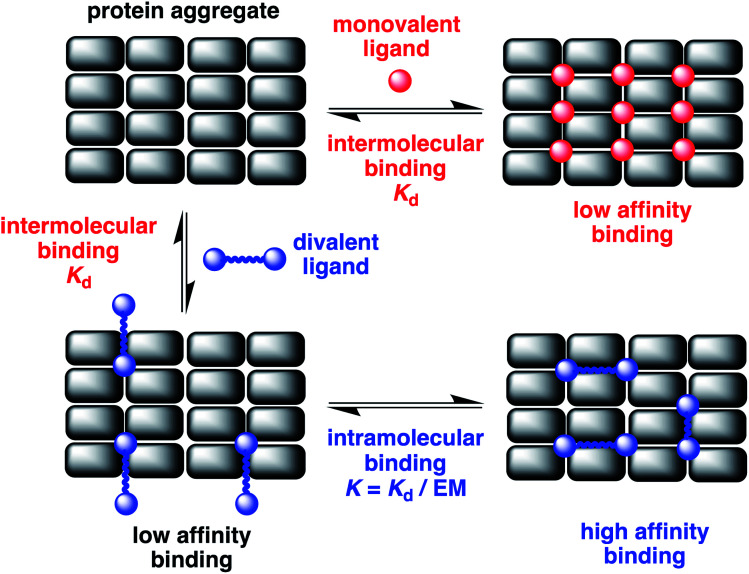
Multivalent binding to protein aggregates. A cross β-sheet fibrillar aggregate presents an organised array of similar binding sites. The binding affinity of monovalent ligands (red) that bind at one site will be low. Divalent ligands (blue) that cooperatively bind at two sites simultaneously can lead to significantly higher binding affinities. The ratio of *K*_d_ for the first intermolecular binding interaction (highlighted in red) compared to the effective molarity (EM) for the second intramolecular binding interaction (highlighted in blue) determines the overall increase in binding affinity.

The misfolding of amyloid proteins generates aggregates rich in β-sheet structures, giving rise to an ordered array of similar binding sites (Fig. 1). When a ligand binds to a single binding site, the affinity is measured by the dissociation constant (*K*_d_). But, if two ligand moieties are linked together, the binding of the first head group at one site will bring the second head group into mutual proximity with another binding site, and this increase in effective molarity (EM) will lead to an enhancement in the overall affinity.^6^ If the linker is too short, simultaneous binding to two head groups at two different binding sites will not be possible. However, if the linker is longer than the binding site separation in the aggregate, cooperative binding of two head groups to two different binding sites should be possible. Values of effective molarity generally fall in the range 10–100 mM, even for long flexible linkers, so provided a ligand head group with a *K*_d_ significantly lower than 10 mM is used, significant enhancements in binding affinity can be expected.^7,8^ Identification of the optimal linker length for different amyloid proteins (Aβ, α-synuclein or tau) will also provide insights into the differences in the binding site topologies of the aggregates, providing new strategies for improving selectivity.

**Fig. 2:**
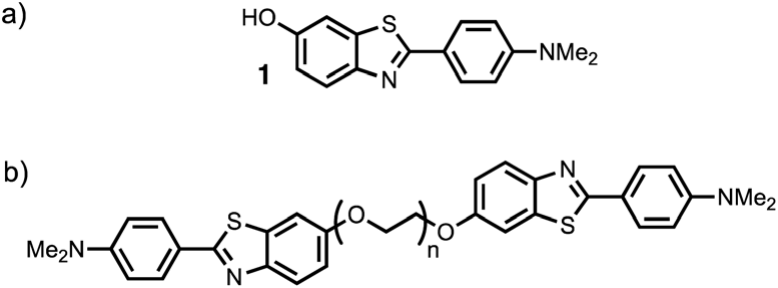
Structures of (a) benzothiazole aniline derivative **1**, and (b) the BTA-PEG_*n*_-BTA dimers.

Thioflavin T dimers linked by short oligoethylene glycol chains were previously found to have enhanced affinity for Aβ40 aggregates.^9^ Thioflavin T is charged, which limits biological applications of these compounds, so we have applied this approach to the neutral benzothiazole aniline head group **1** (Fig. 2).^10^ Benzothiazole anilines (BTA) are fluorescent dyes,^11^ which have been used for the detection of Aβ related amyloidosis.^12,13^ We chose polyethylene glycol (PEG) as the linker, because it is known to improve the solubility, stability, and blood circulation time of drug conjugates, it does not compete for interactions with biological molecules, and a range of different chain lengths are readily accessible.^14^

## Results and discussion

### Monodisperse BTA dimers

**Scheme 1:**
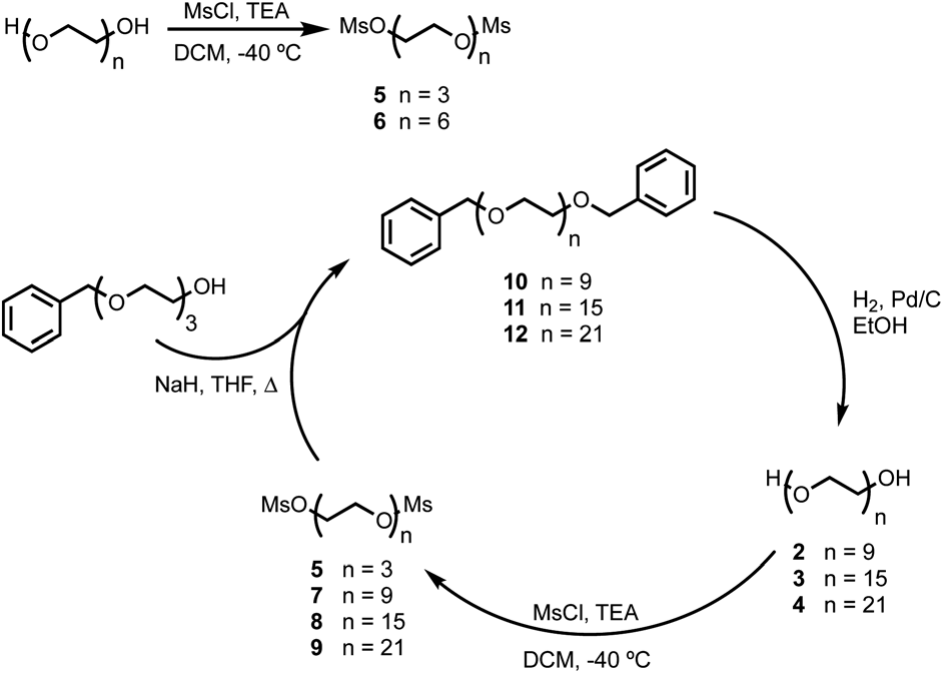
Synthesis of oligoethylene glycol mesylates.

**Scheme 2:**
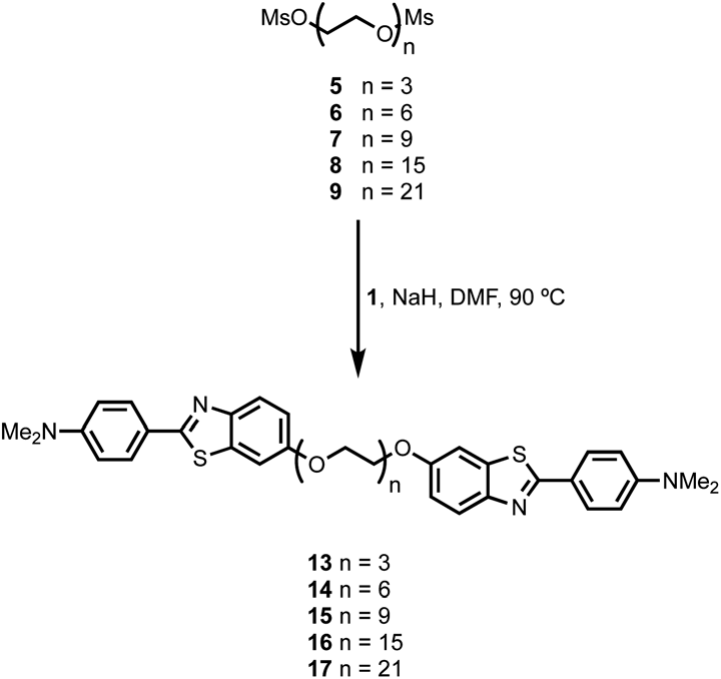
Synthesis of monodisperse BTA-PEG_*n*_-BTA dimers.

BTA dimers were synthesised with oligoethylene glycol linkers between 3 and 21 ethylene glycol units in length. The 3-unit and 6-unit linkers were commercially available. The longer linkers **2**, **3** and **4** were prepared from benzyl triethylene glycol by an iterative sequence of mesylation, Williamson ether coupling, and hydrogenation to remove the benzyl protecting groups (Scheme 1). BTA derivative **1** was synthesized using a three step procedure previously reported.^15^ Coupling of oligoethylene glycol mesylates **5–9** with compound **1** with gave the corresponding BTA dimers **13–17** (Scheme 2), and the final products were purified by HPLC.^16^

**Fig. 3:**
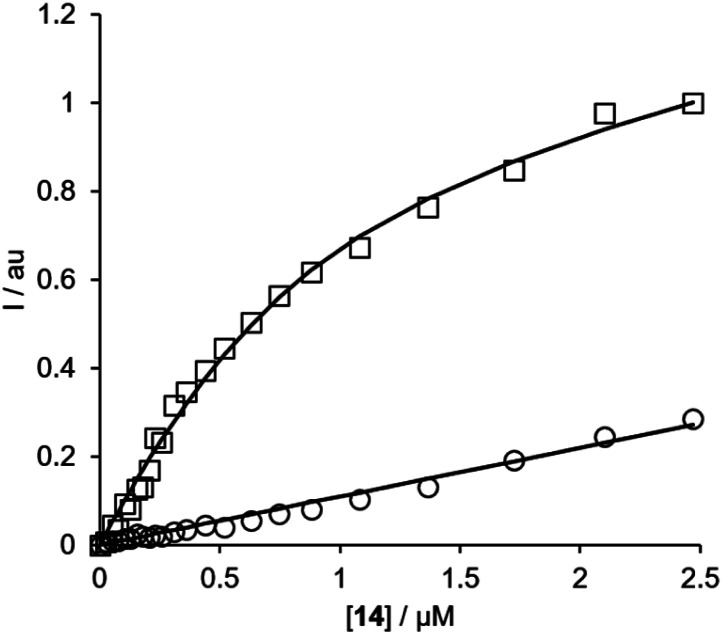
Fluorescence titration of compound **14** into a 1 μM solution of α-synuclein in PBS buffer at pH 7.4 (squares) and into PBS 1× buffer with no protein (circles) at 298 K. The spectra were recorded using 355 nm as excitation wavelength and using the emission spectra at 425 nm. The lines show the best fit to eqn (1)–(3).

For each of the BTA dimers, fluorescence titrations were carried out with fibrils of α-synuclein, Aβ42 and tau in PBS 1× buffered solutions (pH 7.4). Fig. 3 shows typical results from a fluorescence titration of **14** into a 1 μM solution of α-synuclein. The BTA dimers are fluorescent in the absence of protein, so for each titration, the increase in fluorescence measured in the presence of protein was compared with the fluorescence measured for a control sample in the absence of protein. At low concentrations of BTA dimer, there is a sharp increase in the fluorescence emission intensity for the solution containing protein relative to the control. Once saturation is reached, the fluorescence emission from the solution containing protein continues to increase, but by a similar amount to the control. The titration data must therefore be analysed allowing for the emission of both the free and bound BTA dimer. Eqn (1) describes how the emission intensity (*I*) is related to the concentration of free and bound dye.

1 *I* = *ε*_f_*ϕ*_f_[free] + *ε*_b_*ϕ*_b_[bound]

where *ε*_f_*ϕ*_f_ and *ε*_b_*ϕ*_b_ are product of the UV-Vis absorption extinction coefficient and the fluorescence quantum yield for the free and bound BTA dimer respectively.

The free and bound concentrations of the BTA dimer are related to the dissociation constant for binding to the protein fibrils (*K*_d_) by eqn (2) and (3).

2 [L] = [free] + [bound]

3
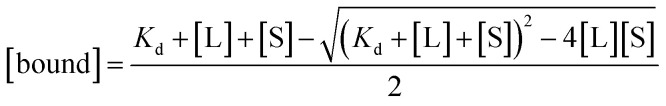
where [L] and [S] are the total concentrations of BTA dimer and protein binding sites respectively.

Eqn (1)–(3) can be fit to the two sets of titration data in Fig. 3 to determine the dissociation constant *K*_d_ and brightness (ef) of the free and bound BTA dimer, and in the case of high affinity binding, the concentration of binding sites on the protein fibrils. In all cases, the titration data fit well to a 1 : 1 binding isotherm, and the lines shown in Fig. 3 are the calculated lines of best fit. For the highest affinity ligands, where the concentration of binding sites is well-defined by the titration data, the values of [S] fall in the range 200–500 nM range, which indicates that there is one BTA dimer binding site for every 2–5 protein monomers in the fibrils. The BTA dimer with the 3-unit linker **13** was not sufficiently soluble to determine accurate dissociation constants, but the results for the other BTA dimers for all three proteins are summarised in Table 1.

**Table 1:** Dissociation constants (*K*_d_/nM) for binding of monodisperse BTA dimers to protein fibrils measured using fluorescent titrations in PBS 1× at pH 7.4 and 298 K^a^

Compound	*n*	α-Synuclein	Protein Aβ42	Tau
**13**	3	—	—	—
**14**	6	190 ± 140	1400 ± 1600	900 ± 700
**15**	9	2.7 ± 1.5	78 ± 26	1000 ± 300
**16**	15	35 ± 15	7 ± 5	110 ± 57
**17**	21	210 ± 180	320 ± 75	280 ± 48

a All measurements were repeated at least three times, and the errors are quoted at the 95% confidence limit. *n* is the number of ethylene glycol units in the linker.

**Fig. 4:**
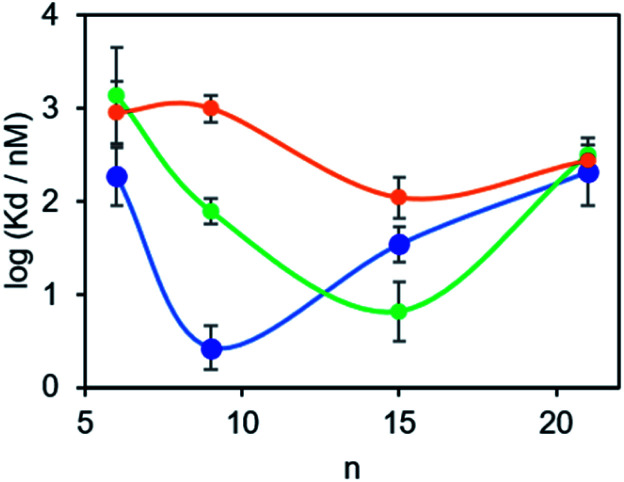
Dissociation constants (*K*_d_) measured for the binding of monodisperse BTA-PEG_*n*_-BTA dimers to solutions of aggregates of α-synuclein (1 μM blue), Aβ42 (0.5 μM green) and tau (0.5 μM orange) in PBS 1× buffer at pH 7.4 at 298 K.

The results in Table 1 are plotted as a function of linker length for each of the three proteins in Fig. 4. For tau, the dissociation constants are in the micromolar range for all of the BTA dimers, and there is little variation with linker length.^17^ However, for the other two proteins, a distinct optimum in linker length is observed. For α-synuclein, the 9-unit linker gives the highest binding affinity with a dissociation constant in the low nanomolar range. For Aβ42, the 15-unit linker gives the lowest dissociation constant, again in the nanomolar range. Thus, variation in linker length can modulate the affinity of a BTA dimer by between two and three orders of magnitude. More interestingly, the selectivity is different for different proteins. Thus the 9-unit linker gives an α-synuclein-selective dye, and the 15-unit linker gives an Aβ42-selective dye, both with nanomolar affinities. The differences in the affinities observed must be related to differences in the arrangement of the binding sites on the surfaces of the fibrils, which provides some indirect information of the three-dimensional structures of the aggregates. A monomeric BTA analogue equipped with an oligoethylene glycol chain was also prepared (see ESI†). The binding affinity of this compound for α-synuclein was too low to measure (*K*_d_ > 1 μM), which shows that the linker is not involved in enhancement of the binding affinity of the BTA dimers.

### Polydisperse BTA dimers

**Scheme 3:**
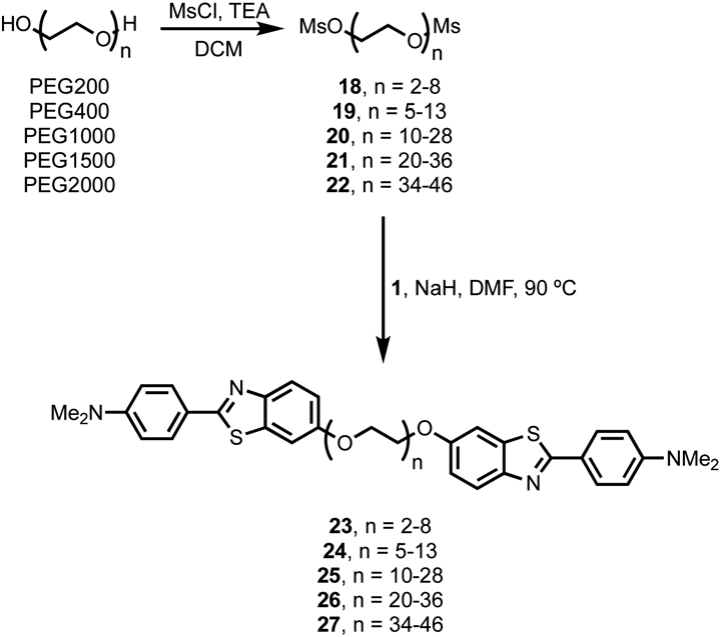
Synthesis of polydisperse BTA-PEG_*n*_-BTA dimers.

**Fig. 5:**
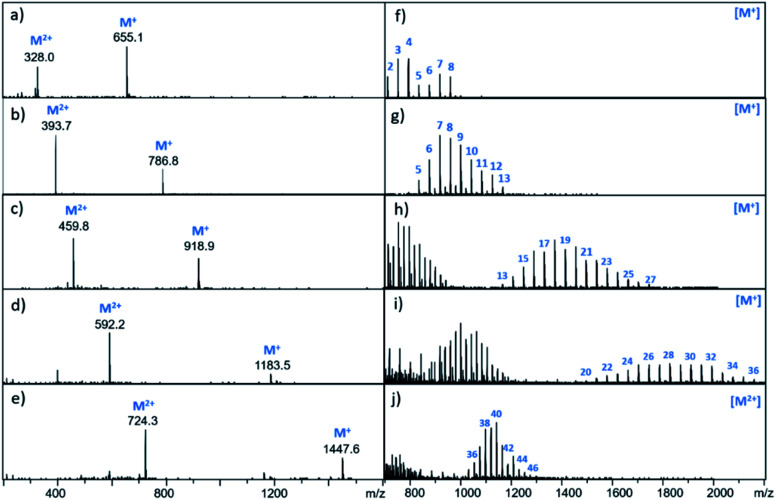
Mass spectra of BTA dimers. The left panel shows the monodisperse BTA dimers (a) **13** (3-unit linker) (b) **14** (6-unit linker) (c) **15** (9-unit linker) (d) **16** (15-unit linker) (e) **17** (21-unit linker). The right panel shows the polydisperse BTA dimers (f) **23**, (g) **24**, (h) **25**, (i) **26**, (j) **27**. Peaks corresponding to the M^+^ and M^2+^ ions are indicated. For the polydisperse samples, individual peaks are labelled with the number of ethylene glycol units in the chain.

It is possible to prepare BTA dimers in a more straightforward manner by using polydisperse PEG linkers. Commercial polydisperse mixtures (PEG200, PEG400, PEG1000, PEG1500 and PEG2000) were functionalised with BTA as shown in Scheme 3. This route provides rapid access to a wide range of linker lengths for screening purposes. The polydisperse mixtures of BTA dimers were obtained with yields of 50–98% over two steps, and the desired products were separated from unfunctionalized PEG starting materials using preparative HPLC. Fig. 5 compares the mass spectra of the polydisperse BTA dimers with the monodisperse compounds described above. The polydisperse samples contain a distribution of chain lengths, and the data in Fig. 5 were used to calculate the average chain length of the polydisperse BTA dimers (*n̄* in Table 2). PEG200 contains the 3-unit and 6-unit linkers, PEG400 contains the 6-unit and 9-unit linkers, PEG1000 contains the 15-unit and 21-unit linkers, and the other two polydisperse BTA dimers are longer than the monodisperse analogues with linkers up to 46 ethylene glycol units in length.

**Table 2:** Apparent dissociation constants (*K*_d_/nM) for binding of polydisperse BTA-PEG_*n*_-BTA dimers to α-synuclein fibrils measured using fluorescent titrations in PBS 1× at pH 7.4 and 298 K^a^

Mixture	*n*	*n̄*	*K*_d_/nM
**23**	2–8	6	—
**24**	5–13	9	13 ± 7
**25**	10–28	20	38 ± 21
**26**	20–36	28	130 ± 75
**27**	34–46	40	>1000

a All measurements were repeated at least three times, and the errors are quoted at the 95% confidence limit. *n* is the range of ethylene glycol units in the linker, and *n̄* is the average number of ethylene glycol units in the linker.

**Fig. 6:**
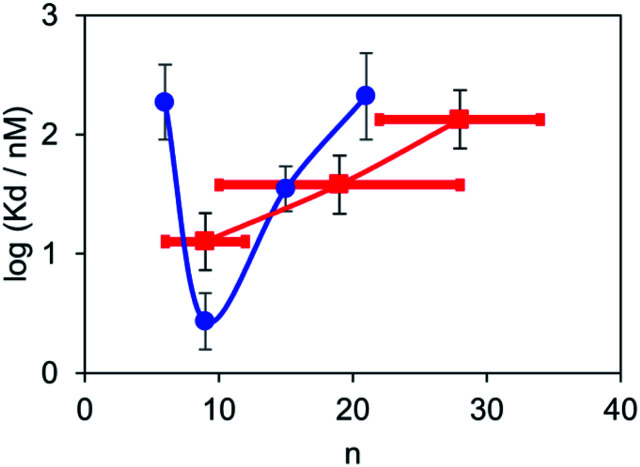
Dissociation constants (*K*_d_) measured for the binding of monodisperse (blue) and polydisperse BTA dimers (red) to 1 μM solutions of α-synuclein aggregates in PBS 1× buffer at pH 7.4 at 298 K. The points for the polydisperse mixtures are plotted at the average linker length *n̄*, and the horizontal bars represent the range of values of n observed in the mass spectra.

Fluorescent titrations with the polydisperse mixtures of BTA dimers were used to measure the apparent binding affinities for α-synuclein fibrils in the same way as described above for the monodisperse BTA dimers. The titration data fit well to a 1 : 1 binding isotherm allowing for the fluorescence emission from the free dye as described above, and the results are summarised in Table 2. The BTA dimer with the shortest polydisperse linker obtained from PEG200 was not sufficiently soluble to allow accurate measurement of the dissociation constant, and the dissociation constant for the longest polydisperse linker obtained from PEG2000 was too low to be measured. The dissociation constants for the other polydisperse BTA dimers are in the same range as those measured for the monodisperse analogues. In this case, the observed values are a weighted average of the dissociation constants of all of the dimers present, and Fig. 6 compares the results obtained for the monodisperse and polydisperse BTA dimers. The dependence on linker length observed for the polydisperse BTA dimers is similar to that observed for the monodisperse dimers, and the highest binding affinity was found for the mixture with an average linker length of 9 ethylene glycol units. These results show that polydisperse mixtures of linkers could be used to provide a rapid screen of a large number of different linker lengths to identify where to focus efforts on the synthesis of monodisperse compounds with high affinity.

### Total internal reflection fluorescence microscopy (TIRFM) imaging

**Fig. 7:**
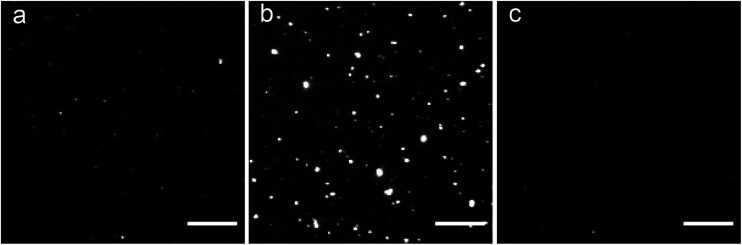
TIRFM images of α-synuclein sonicated fibrils in the presence of (a) 4 nM **15**, (b) 200 nM **15** and (c) 5 μM ThT. Fibrils were obtained from 200 nM samples of the monomer. All images have the same contrast. Scale bar 10 μm.

The highest affinity BTA dimers were investigated as fluorescence imaging agents for protein aggregates. Fig. 7 compares *in vitro* imaging of α-synuclein aggregates with **15** and with thioflavin T (ThT). ThT is widely used to image aggregates, because it has a very low fluorescence emission intensity in the free state, and there is a large increase in fluorescence emission on binding to protein aggregates. The corresponding change in fluorescence emission intensity is much lower for the BTA dimers described here (see Fig. 3). However, the localisation of **15** provided by high affinity binding to the aggregates means that the dye can be used at very low concentrations with minimal background emission from the free dye. Fig. 7 shows that the images obtained with nanomolar solutions of the BTA dimer are brighter than the images obtained with micromolar solutions of ThT, which is consistent with the three orders of magnitude increase in binding affinity measured for **15** above.

**Fig. 8:**
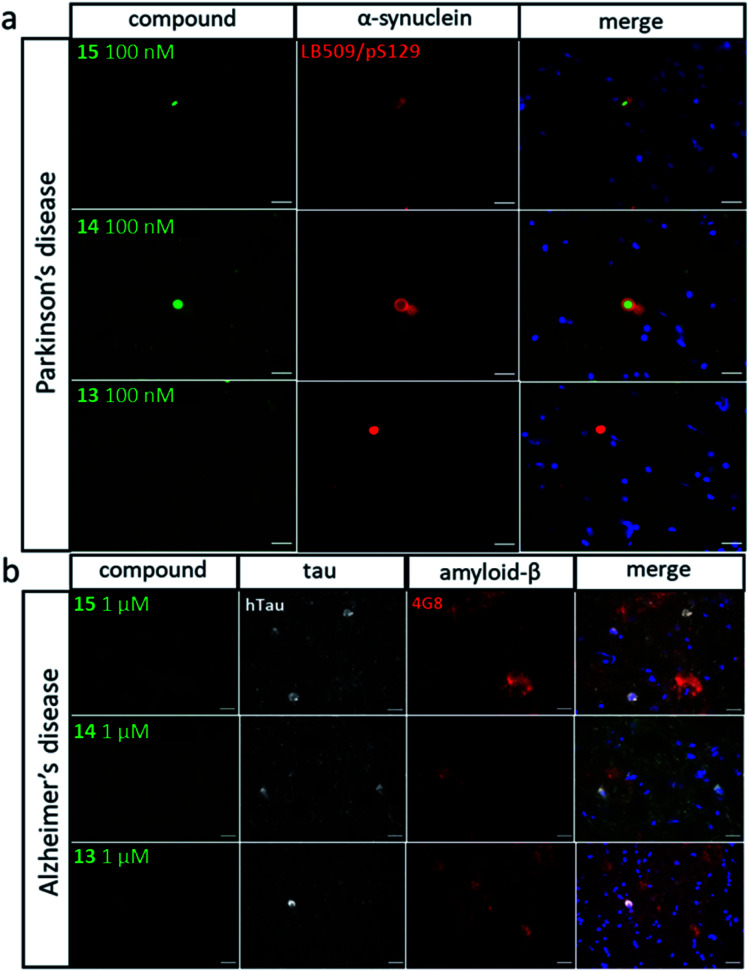
Fluorescence microscope images of human brain sections. (a) Sections from patients with Parkinson's disease labelled with antibodies against α-synuclein (LB509 and pS129) and stained with compounds **13**, **14** and **15** (100 nM). (b) Sections from patients with Alzheimer's disease labelled with antibodies against tau (dako tau) and Aβ (4G8) and stained with compounds **13**, **14** and **15** (1 μM). Scale bar 20 μm.

Fig. 8 shows the results for imaging of aggregates in human tissue. Brain sections from patients with Parkinson's disease were labelled with antibodies against α-synuclein (LB509 and pS129) and stained with compounds **13**, **14** and **15** (Fig. 8a). LB509/pS129-positive Lewy bodies, that were identified as cytoplasmic spheres, were stained by both compounds **14** and **15** but not by compound **13**. Compound **14** shows significant amounts of co-localisation with the antibodies elsewhere in the brain section. To assess any potential cross-reactivity with other pathological amyloid aggregates, such as Aβ plaques and neurofibrillary tangles of tau, brain sections from patients with Alzheimer's disease were labelled with antibody against tau (dako tau) and Aβ (4G8) and stained with compounds **13**, **14** and **15** (Fig. 8b). Tau-positive neuronal inclusions and 4G8-positive extracellular plaques were identified and showed no staining by any of the compounds at concentrations one order of magnitude higher than that which stained Lewy bodies. This result shows that compounds **14** and **15** both selectively bind α-synuclein aggregates in human brain tissue.

## Conclusions

Cross β-sheet protein aggregates present an ordered array of binding sites that are ideally suited for a multivalent approach to obtain high affinity ligands. In order to explore this strategy, we have synthesised a series of ligands composed of oligoethylene glycol linkers connecting two benzothiazole aniline (BTA) head groups. The BTA head groups show micromolar affinities for amyloid aggregates, when bound as monomeric dyes. In contrast, some of the BTA dimers described here show dissociation constants in the low nanomolar range. Two series of BTA dimers were synthesised: in the monodisperse series, the head groups were connected by five different linkers between 3 and 21 ethylene glycol units in length; in the polydisperse series, the head groups were connected by mixtures of linkers with an average number of ethylene glycol units ranging from 6 to 40 in length. The dissociation constants for binding of the monodisperse BTA dimers to Aβ, α-synuclein and tau fibrils were measured by fluorescence titration experiments. In the case of tau, all of the BTA dimers bind with micromolar affinities, but for Aβ and for α-synuclein, low nanomolar affinities were observed. Moreover, there is a clear dependence of binding affinity on the length of the linker connecting the two head groups. For Aβ, the highest affinity was observed for the 15-unit linker, and for α-synuclein, the highest affinity was observed for the 15-unit linker. This result suggests that there are repeating binding sites on Aβ aggregates that are spaced further apart than those on the surface of α-synuclein aggregates. Thus it is possible to achieve selectivity for different proteins based on the topological distribution of binding sites rather than based on binding site selectivity. This discovery opens new avenues for the development of high affinity, high selectivity based on multivalent ligands.

Excellent results were obtained when the highest affinity α-synuclein ligands were tested as imaging agents *in vitro*. Although binding of BTA dyes to protein aggregates leads to a relatively small increase in optical brightness, the very high affinities of the BTA dimers means that they can be used at nanomolar concentrations where there is almost no background from unbound dye. The BTA dimers were also shown to selectively image α-synuclein aggregates in human brain tissue. Two of the dimers stained α-synuclein aggregates in brain sections from patients with Parkinson's disease and did not stain either Aβ or tau aggregates in brain sections from patients with Alzheimer's disease. The results demonstrate that multivalent ligands offer a powerful approach to obtain high affinity, selective reagents to bind the fibrillary aggregates that form in neurodegenerative disease.

## Author contributions

CAH, DK, FIA and MGS designed the experiments. ES carried out the synthesis and binding studies. KK and TSC prepared and characterised the protein aggregates. MR carried out the in vitro imaging. SGF carried out the tissue imaging. All authors contributed to the writing the text.

## Conflicts of interest

There are no conflicts to declare.

## Supplementary Material

SC-012-D1SC01263K-s001

## References

[cit1] Kumar S., Udgaonkar J. B. (2010). Curr. Sci..

[cit2] Iadanza M. G., Jackson M. P., Hewitt E. W., Ranson N. A., Radford S. E. (2018). Nat. Rev. Mol. Cell Biol..

[cit3] Krebs M. R. H., Bromley E. H. C., Donald A. M. (2005). J. Struct. Biol..

[cit4] De S., Whiten D. R., Ruggeri F. S., Hughes C., Rodrigues M., Sideris D. I., Taylor C. G., Aprile F. A., Muyldermans S., Knowles T. P. J., Vendruscolo M., Bryant C., Blennow K., Skoog I., Kern S., Zetterberg H., Klenerman D. (2019). Acta Neuropathol. Commun..

[cit5] Cheng Y., Ono M., Kimura H., Ueda M., Saji H. (2012). J. Med. Chem..

[cit6] Zhou Y., Zhang H., Liu L., Li C., Chang Z., Zhu X., Ye B., Xu M. (2016). Sci. Rep..

[cit7] Hunter C. A., Anderson H. L. (2009). Angew. Chem., Int. Ed..

[cit8] Kramer R. H., Karpen J. W. (1998). Nature.

[cit9] Krishnamurthy V., Semetey V., Bracher P., Shen N., Whitesides G. (2007). J. Am. Chem. Soc..

[cit10] Qin L., Vastl J., Gao J. (2010). Mol. BioSyst..

[cit11] Diner I., Dooyema J., Gearing M., Walker L. C., Seyfried N. T. (2017). Bioconjug. Chem..

[cit12] Mathis C. A., Wang Y., Holt D. P., Huang G. -F., Debnath M. L., Klunk W. E. (2003). J. Med. Chem..

[cit13] Lockhart A., Lamb J. R., Osredkar T., Sue L. I., Joyce J. N., Ye L., Libri V., Leppert D., Beach T. G. (2007). Brain.

[cit14] Bulic B., Pckhardt M., Mandelkow E. (2013). J. Med. Chem..

[cit15] Liese S., Netz R. R. (2018). ACS Nano.

[cit16] Ahmed S., Tanaka M. (2006). J. Org. Chem..

[cit17] Klunk W. E., Wang Y., Huang G.-F., Debnath M. L., Holt D. P., Shao L., Hamilton R. L., Ikonomovic M. D., DeKosky S. T., Mathis C. A. (2003). J. Neurosci..

