# Supplementary material for: Mapping the binding site topology of amyloid protein aggregates using multivalent ligands
Source: Chem Sci. 2021 Jun 7;12(25):8892–9. doi: 10.1039/d1sc01263k (PMC8246084; doi:10.1039/d1sc01263k)
Supplement: SC-012-D1SC01263K-s001 [file SC-012-D1SC01263K-s001.pdf]

Electronic Supplementary Material (ESI) for Chemical Science.
This journal is © The Royal Society of Chemistry 2021

Electronic Supporting Information

Mapping the Binding Site Topology of Amyloid Protein Aggregates using Multivalent Ligands

Elena Sanna,[†] Margarida Rodrigues,[†] Steven G. Fagan,[‡] Timothy S. Chisholm, Klara Kulenkampff, David Klenerman,[†] Maria Grazia Spillantini,[‡] Franklin I. Aigbirhio[⊥] and Christopher A. Hunter*,[†]

† Department of Chemistry, University of Cambridge, Lensfield Road, Cambridge CB2 1EW, United Kingdom.

‡ Department of Clinical Neurosciences, Clifford Allbutt Building, University of Cambridge, Cambridge, CB2 0AH, United Kingdom.

⊥ Department of Clinical Neuroscience, Wolfson Brain Imaging Centre, University of Cambridge, CB2 0QQ, United Kingdom.

Email: herchelsmith.orgchem@cam.ac.uk.

1. General experimental details

All the reagents and materials used in the synthesis of the compounds described below were bought from commercial sources, without prior purification. UV irradiations were performed using a UVP lamp model UVGL-58 (1x365 nm tube, 6 wattss) and a UVP lamp model UVL-28 (2x365 nm tubes, 8 watts). Thin layer chromatography was carried out using silica gel 60F (Merck) on glass plates. Flash chromatography was carried out on an automated system (Combiflash Companion, Combiflash Rf+ or Combiflash Rf Lumen) using prepacked cartridges of silica (25µ or 50µ PuriFlash® Columns). ^{1}H and ^{13}C NMR spectra were recorded on either a Bruker AV3400 or AV3500 spectrometer at 298 K unless specifically stated otherwise. Residual solvent was used as an internal standard. All chemical shifts are quoted in ppm on the δ scale and the coupling constants expressed in Hz. Signal splitting patterns are described as follows: s (singlet), d (doublet), t (triplet), q (quartet), m (multiplet). UV-Vis spectroscopic data were recorded using a Cary 60 spectrophotometer (Agilent). Fluorescence spectroscopic data were recorded using a Cary Eclipse fluorescence spectrophmeter (Agilent). FT-IR spectra were recorded on a PerkinElmer Spectrum One FT-IR spectrometer equipped with a ATR cell. The microwave used was Biotage Initiator+. Preparative HPLC were performed in an Agilent HP1100. The LCMS analysis of samples was performed using a Waters Acquity H-Class UPLC coupled with a single quadrupole Waters SQD2 or a Waters Xevo G2-S bench top QTOF machine.

2. Synthesis and charaterization of the described compounds

2-(4-(dimethylamino)phenyl)benzo[d]thiazol-6-ol (1)

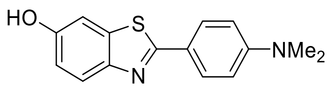

The product was synthesized following a previously reported procedure.[1]

General procedure for the mesylation of PEG compounds

The synthesis was performed following a previously reported procedure.[2] In a typical experiment: 2.5 equivalents of methanesulfonyl chloride were added dropwise at -40 °C to a solution of 1 equivalent of diol and 3 equivalents of TEA in 15 mL of anhydrous CH_2Cl_2. The resulting solution was stirred overnight at room temperature. Then, the solution was extracted with water (40 mL). The aqueous layer was re-extracted with CH_2Cl_2 (3 x 5 mL). The combined organic fractions were dried over anhydrous Na_2SO_4, filtered, and concentrated. The residue was purified by flash chromatography (silica, CH_2Cl_2:EtOAc (0-100 %) then, EtOAc:MeOH (0-50%)).

Compound 5

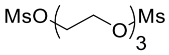

97% yield. ^{1}H NMR ($CDCl_3$, 400 MHz) δ (ppm): 4.43 – 4.35 (m, 4H), 3.83 – 3.76 (m, 4H), 3.70 (s, 4H), 3.09 (s, 6H). ^{13}C NMR ($CDCl_3$, 100.6 MHz) δ (ppm): 70.5, 69.3, 68.9, 37.6. ESI-HRMS(+) m/z (%): calc. $C_8H_{18}O_8S_2$ 307.0521; exp. 307.0527 $[M+H]^+$; IR (ATR, cm^{-1}): 1339, 1168, 1013, 971, 911, 796, 729, 524, 451.

Compound 6

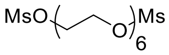

66% yield. ^{1}H NMR ($CDCl_3$, 400 MHz) δ (ppm): 4.43 – 4.33 (m, 4H), 3.80 – 3.73 (m, 4H), 3.70 – 3.55 (m, 16H), 3.08 (s, 6H). ^{13}C NMR ($CDCl_3$, 100.6 MHz) δ (ppm): 70.8, 70.8, 70.7, 69.5, 69.2, 37.9. ESI-HRMS(+) m/z (%): calc. $C_{14}H_{31}O_{11}S_2$ 439.1308; exp. 439.1285 $[M+H]^+$. IR (ATR, cm^{-1}): 3021, 2872, 1745, 1455, 1413, 1344, 1249, 1169, 1099, 1013, 971, 912, 797, 731, 525, 454, 418.

Compound 7

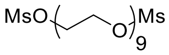

97% yield. ^{1}H NMR ($CDCl_3$, 400 MHz) δ (ppm): 4.42 – 4.34 (m, 4H), 3.80 – 3.73 (m, 4H), 3.71 – 3.57 (m, 28H), 3.09 (s, 6H). ^{13}C NMR ($CDCl_3$, 100.6 MHz) δ (ppm): 70.8, 70.7, 70.6, 69.4, 69.1, 37.8. ESI-HRMS(+) m/z (%): calc. $C_{20}H_{42}O_{14}S_2$ 571.2094; exp. 571.2087 $[M+H]^+$; IR (ATR, cm^{-1}): 2872, 1348, 1172, 1105, 919, 807, 528.

Compound 8

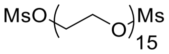

81% yield. ^{1}H NMR ($CDCl_3$, 400 MHz) δ (ppm): 4.43 – 4.33 (m, 4H), 3.80 – 3.73 (m, 4H), 3.64 (d, *J* = 3.0 Hz, 52H), 3.08 (s, 6H). ^{13}C NMR ($CDCl_3$, 100.6 MHz) δ (ppm): 70.7, 70.6, 70.5, 69.3, 69.0, 37.7. ESI-HRMS(+) m/z (%): calc. $C_{32}H_{67}O_{20}S_2$ 835.3667; exp. 835.3647 $[M+H]^+$; IR (ATR, cm^{-1}): 2873, 1457, 1349, 1301, 1249, 1173, 1104, 1016, 973, 921, 862, 804, 717, 560, 524, 498, 480, 462, 439, 420.

Compound 9

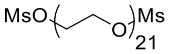

90% yield. ^{1}H NMR ($CDCl_3$, 400 MHz) δ (ppm): 4.43 – 4.32 (m, 4H), 3.79 – 3.73 (m, 4H), 3.64 (m, 76H), 3.08 (s, 6H). ^{13}C NMR ($CDCl_3$, 100.6 MHz) δ (ppm): 70.6, 70.5, 69.2, 68.9, 37.7. ESI-HRMS(+) m/z (%): calc. $C_{44}H_{91}O_{26}S_2$ 1099.5240; exp. 1099.5236 $[M+H]^+$; IR (ATR, cm^{-1}): 2922, 2872, 2856, 1456, 1348, 1248, 1173, 1105, 1017, 973, 920, 809, 729, 566, 549, 527, 507, 495, 453, 425.

Dimesylated PEG200 (Compound 18)

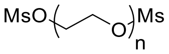

73%. ^{1}H NMR ($CDCl_3$, 400 MHz) δ (ppm): 4.40 – 4.34 (m, 4H), 3.82 – 3.74 (m, 4H), 3.71 – 3.60 (m, ~6H), 3.10 – 3.03 (m, 6H). ^{13}C NMR ($CDCl_3$, 100.6 MHz) δ (ppm): 70.7, 70.7, 70.6, 69.3, 69.2, 69.1, 69.1, 37.8. IR (ATR, cm^{-1}): 3028, 2938, 2874, 1454, 1413, 1340, 1249, 1167, 1130, 1107, 1013, 971, 908, 796, 731, 524, 454.

Dimesylated PEG400 (Compound 19)

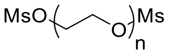

79%. ^{1}H NMR ($CDCl_3$, 400 MHz) δ (ppm): 4.41 – 4.34 (m, 4H), 3.80 – 3.73 (m, 4H), 3.68 – 3.60 (m, ~24H), 3.08 (s, 6H). ^{13}C NMR ($CDCl_3$, 100.6 MHz) δ (ppm): 70.7, 70.6, 70.6, 69.4, 69.1, 37.8. IR (ATR, cm^{-1}): 3022, 2873, 1730, 1641, 1454, 1413, 1344, 1249, 1170, 1093, 1013, 972, 915, 800, 732, 526, 455.

Dimesylated PEG1000 (Compound 20)

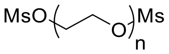

56 %. ^{1}H NMR ($CDCl_3$, 400 MHz) δ (ppm): 4.46 – 4.32 (m, 4H), 3.82 – 3.74 (m, 4H), 3.67 (s, ~66H), 3.11 (s, 6H). ^{13}C NMR ($CDCl_3$, 100.6 MHz) δ (ppm): 70.6, 70.5, 70.5, 69.3, 69.0, 37.7. IR (ATR, cm^{-1}): 2870, 1723, 1453, 1348, 1283, 1248, 1173, 1092, 1018, 972, 918, 841, 802, 730, 699, 527, 454, 424.

Dimesylated PEG1500 (Compound 21)

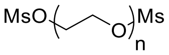

62%. ^{1}H NMR ($CDCl_3$, 400 MHz) δ (ppm): 4.42 – 4.33 (m, 4H), 3.80 – 3.73 (m, 4H), 3.64 (m, ~100H), 3.08 (s, 6H). ^{13}C NMR ($CDCl_3$, 100.6 MHz) δ (ppm): 70.6, 70.5, 70.5, 69.3, 69.0, 37.7. IR (ATR, cm^{-1}): 2867, 2362, 1645, 1452, 1348, 1298, 1249, 1173, 1091, 1039, 946, 919, 843, 805, 729, 699, 527, 454.

Dimesylated PEG2000 (Compound 22)

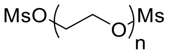

40%. ^{1}H NMR ($CDCl_3$, 400 MHz) δ (ppm): 4.43 – 4.34 (m, 4H), 3.91 – 3.70 (m, 4H), 3.64 (s, ~102H), 3.09 (s, 6H). ^{13}C NMR ($CDCl_3$, 100.6 MHz) δ (ppm): 70.7, 70.6, 70.5, 69.3, 69.0, 37.8. IR (ATR, cm^{-1}): 2869, 1642, 1466, 1344, 1279, 1242, 1173, 1093, 946, 921, 841, 807, 729, 699, 527, 473, 461.

General procedure for the preparation of benzylated PEG

The synthesis was performed following a previously reported procedure.[3] In a typical experiment: 1 equivalent of the corresponding dimesylated PEG compound in 20 mL of THF_{anh} was added to a solution of 2.2 equivalents of trietyleneglycol monobenzyl ether and 11 equivalents of NaH in 5 mL of anhydrous THF. The resulting solution was reflux overnight. Then, 10 mL of HCl 1M were added to quench the excess of NaH and stirred 10 minutes. The solution was concentrated under reduced pressure. The residue was dissolved in 10 mL of CH_2Cl_2 and 10 mL of H_2O. The aqueous layer was re-extracted with CH_2Cl_2 (2 x 10 mL). The combined organic fractions were dried over anhydrous Na_2SO_4, filtered, and concentrated. The residue was purified by flash column chromatography (silica, CH_2Cl_2:EtOAc (0-100 %) then, EtOAc:MeOH (0-50%)).

Compound 10

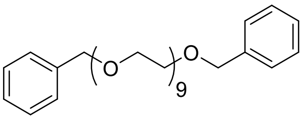

73 % yield. ^{1}H NMR ($CDCl_3$, 400 MHz) δ (ppm): 7.43 – 7.26 (m, 10H), 4.56 (s, 4H), 3.93 – 3.38 (m, 36H). ^{13}C NMR ($CDCl_3$, 100.6 MHz) δ (ppm): 138.2, 128.3, 127.7, 127.5, 73.2, 70.6, 70.6, 70.5, 69.4. ESI-HRMS(+) m/z (%): calc. $C_{32}H_{50}O_{10}$ 595.3482; exp. 595.3481 $[M+H]^+$; IR (ATR, cm^{-1}): 3510, 3494, 2866, 1453, 1349, 1296, 1248, 1172, 1098, 1024, 973, 920, 842, 801, 741, 699, 527.

Compound 11

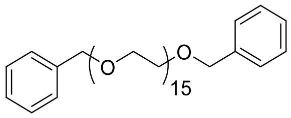

60 % yield. ^{1}H NMR ($CDCl_3$, 400 MHz) δ (ppm): 7.41 – 7.29 (m, 10H), 4.59 (s, 4H), 3.67 (m, 60H). ^{13}C NMR ($CDCl_3$, 100.6 MHz) δ (ppm): 138.4, 128.5, 127.8, 127.7, 73.3, 70.8, 70.7, 70.7, 69.6. ESI-HRMS(+) m/z (%): calc. $C_{60}H_{87}N_4O_{16}S_2$ 1183.5553; exp. 1183.5539 $[M+H]^+$; IR (ATR, cm^{-1}): 2865, 1453, 1349, 1325, 1298, 1249, 1100, 1039, 948, 849, 740, 699, 528.

Compound 12

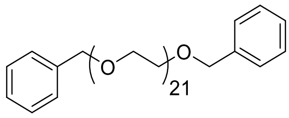

78 % yield. . ^{1}H NMR ($CDCl_3$, 400 MHz) δ (ppm): 7.45 – 7.29 (m, 10H), 4.58 (d, *J* = 1.8 Hz, 4H), 3.87 – 3.47 (m, 84H). ^{13}C NMR ($CDCl_3$, 100.6 MHz) δ (ppm): 138.2, 128.3, 128.3, 127.7, 127.7, 127.5, 73.2, 73.2, 70.6, 70.6, 70.5, 70.3, 69.4, 69.3, 61.7. ESI-HRMS(+) m/z (%): calc. $C_{72}H_{111}N_4O_{22}S_2$ 1447.7126; exp. 1447.7100 $[M+H]^+$; IR (ATR, cm^{-1}): 3481, 2865, 1606, 1495, 1453, 1350, 1296, 1249, 1100, 946, 881, 847, 740, 699.

General procedure for the preparation of long PEG

The product was performed following a previously reported procedure. In a typical experiment: 1 equivalent of dibenzylated PEG and 5 equivalents of Pd/C 10% were dissolved in 8 mL of absolute EtOH. The solution was hydrogenated with stirring from four hours to overnight. Then, the solution was filtered through celite and concentrated.

Compound 2

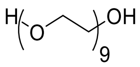

95 % yield. ^{1}H NMR ($CDCl_3$, 400 MHz) δ (ppm): 3.76 – 3.57 (m, 36H). ^{13}C NMR ($CDCl_3$, 100.6 MHz) δ (ppm): 72.6, 70.7, 70.7, 70.6, 70.5, 61.8. ESI-HRMS(+) m/z (%): calc. $C_{18}H_{39}O_{10}$ 415.2543; exp. 415.2550 $[M+H]^+$; IR (ATR, cm^{-1}): 3444, 3419, 2869, 1453, 1350, 1298, 1249, 1105, 947, 885, 485.

Compound 3

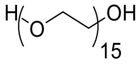

98 % yield. ^{1}H NMR ($CDCl_3$, 400 MHz) δ (ppm): 3.88 – 3.39 (m, 60H). ^{13}C NMR ($CDCl_3$, 100.6 MHz) δ (ppm): 72.6, 70.7, 70.5, 61.8. ESI-HRMS(+) m/z (%): calc. $C_{30}H_{61}O_{16}Na$ 701.3936; exp. 701.3954 $[M+Na]^+$; IR (ATR, cm^{-1}): 3474, 2866, 1457, 1349, 1293, 1251, 1100, 946, 885, 838, 569, 555, 532, 411.

Compound 4

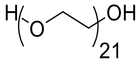

90 % yield. ^{1}H NMR ($CDCl_3$, 400 MHz) δ (ppm): 3.99 – 3.35 (m, 84H). ^{13}C NMR ($CDCl_3$, 100.6 MHz) δ (ppm): 72.8, 72.6, 70.6, 70.3, 70.3, 61.7, 61.6. ESI-HRMS(+) m/z (%): calc. $C_{42}H_{86}O_{22}Na$ 965.5503; exp. 965.5472 $[M+Na]^+$. IR (ATR, cm^{-1}): 3422, 2868, 1454, 1349, 1291, 1248, 1100, 939, 886, 534.

General procedure for the preparation of dimers

The product was synthesized following a previously reported procedure.[4] In a typical experiment: 1 equivalent of mesylated PEG in 1 mL of DMF_{anh} was added to a solution of 2.2 equivalents of **1** and 8 equivalents of NaH in 5 mL of anhydrous DMF. The resulting solution was stirred overnight at 90 °C. Then, 3 mL of aqueous NH_4Cl conc. were added and the solution was stirred 5 minutes. The solution was extracted with CH_2Cl_2 (3 × 10 mL) until no color is observed in the organic layer. The combined organic fractions were dried over anhydrous Na_2SO_4, filtered, and concentrated. The residue was purified by flash column chromatography (silica, CH_2Cl_2:EtOAc (0-100 %) then, EtOAc:MeOH (0-50%)).

Compound 13

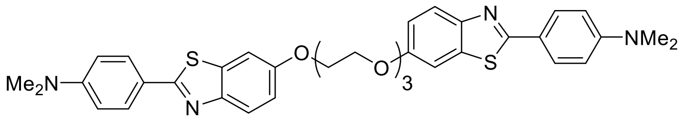

98 % yield. ^{1}H NMR ($CDCl_3$, 400 MHz) δ (ppm): 7.88 (d, J = 8.9 Hz, 4H), 7.84 (d, J = 8.8 Hz, 2H), 7.31 (d, J = 2.5 Hz, 2H), 7.05 (dd, J = 8.8, 2.5 Hz, 2H), 6.72 (d, J = 8.9 Hz, 4H), 4.23 – 4.15 (m, 4H), 3.95 – 3.86 (m, 4H), 3.79 (s, 4H), 3.04 (s, 12H). ^{13}C NMR ($CDCl_3$, 100.6 MHz) δ (ppm): 166.5, 156.2, 151.9, 151.7, 149.0, 135.7, 128.5, 122.6, 121.6, 115.4, 111.7, 105.4, 70.8, 70.6, 70.5, 70.5, 69.7, 69.6, 68.1, 40.2. ESI-HRMS(+) m/z (%): calc. $C_{36}H_{39}N_4O_4S_2$ 655.2407; exp. 655.2399 $[M+H]^+$; IR (ATR, cm^{-1}): 1607, 1559, 1489, 1448, 1413, 1365, 1284, 1262, 1226, 1190, 1168, 1127, 1067, 1040, 941, 819, 647, 625, 603, 480.

Compound 14

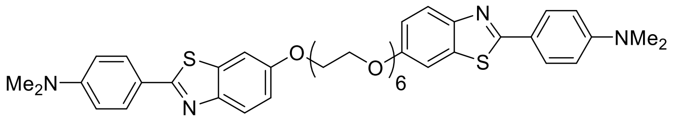

58 %. ^{1}H NMR ($CDCl_3$, 400 MHz) δ (ppm): 7.88 (d, *J* = 8.9 Hz, 4H), 7.84 (d, *J* = 8.9 Hz, 2H), 7.31 (d, *J* = 2.5 Hz, 2H), 7.04 (dd, *J* = 8.9, 2.5 Hz, 2H), 6.72 (d, *J* = 9.0 Hz, 4H), 4.20 – 4.12 (m, 4H), 3.90 – 3.82 (m, 4H), 3.75 – 3.69 (m, 4H), 3.69 – 3.59 (m, 12H), 3.02 (s, 12H). ^{13}C NMR ($CDCl_3$, 100.6 MHz) δ (ppm): 166.8, 156.4, 152.1, 149.2, 135.9, 128.7, 122.8, 121.8, 115.6, 111.9, 105.6, 71.0, 70.8, 70.7, 69.9, 68.3, 40.4. ESI-HRMS(+) m/z (%): calc. $C_{42}H_{51}N_4O_7S_2$ 787.3194; exp. 787.3170 $[M+H]^+$; IR (ATR, cm^{-1}): 3066, 2884, 2816, 1605, 1560, 1530, 1491, 1448, 1362, 1319, 1283, 1262, 1223, 1189, 1168, 1116, 1099, 1065, 1005, 942, 883, 818, 728, 700, 689, 627, 590, 557, 538, 516, 478, 436.

Compound 15

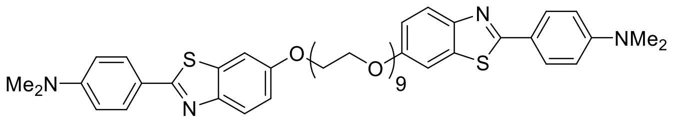

53 % yield. ^{1}H NMR ($CDCl_3$, 400 MHz) δ (ppm): 7.89 (d, *J* = 9.0 Hz, 4H), 7.84 (d, *J* = 8.9 Hz, 2H), 7.33 (d, *J* = 2.6 Hz, 2H), 7.05 (dd, *J* = 8.9, 2.5 Hz, 2H), 6.73 (d, *J* = 9.0 Hz, 4H), 4.25 – 4.11 (m, 4H), 3.95 – 3.83 (m, 4H), 3.80 – 3.54 (m, 28H), 3.04 (s, 12H). ^{13}C NMR ($CDCl_3$, 100.6 MHz) δ (ppm): 166.6, 156.3, 152.0, 149.1, 135.8, 128.6, 122.8, 121.7, 115.5, 111.8, 105.5, 70.9, 70.7, 70.6, 69.8, 68.2, 40.3. ESI-HRMS(+) m/z (%): calc. $C_{48}H_{62}N_4O_{10}S_2$ 919.3986; exp. 919.4024 $[M+H]^+$; IR (ATR, cm^{-1}): 2919, 2871, 1607, 1560, 1532, 1492, 1449, 1363, 1285, 1264, 1224, 1190, 1116, 1068, 1036, 943, 859, 817, 452.

Compound 16

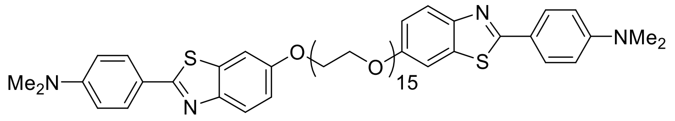

77 % yield. ^{1}H NMR ($CDCl_3$, 400 MHz) δ (ppm): 7.86 (d, *J* = 8.7 Hz, 4H), 7.81 (d, *J* = 8.9 Hz, 2H), 7.30 (d, *J* = 2.5 Hz, 2H), 7.02 (dd, *J* = 8.9, 2.5 Hz, 2H), 6.69 (d, *J* = 8.7 Hz, 4H), 4.15 (t, *J* = 4.8 Hz, 4H), 3.85 (t, *J* = 4.8 Hz, 4H), 3.74 – 3.53 (m, 52H), 3.00 (s, 12H). ^{13}C NMR ($CDCl_3$, 100.6 MHz) δ (ppm): 166.5, 156.2, 151.9, 149.0, 135.7, 128.5, 122.7, 121.6, 115.4, 111.7, 105.4, 70.8, 70.6, 70.5, 69.7, 68.1, 40.2. ESI-HRMS(+) m/z (%): calc. $C_{60}H_{87}N_4O_{16}S_2$ 1183.5553; exp. 1183.5539 $[M+H]^+$; IR (ATR, cm^{-1}): 2868, 1677, 1606, 1559, 1527, 1491, 1450, 1415, 1349, 1314, 1284, 1263, 1225, 1189, 1110, 1069, 943, 820, 516, 417.

Cmpound 17

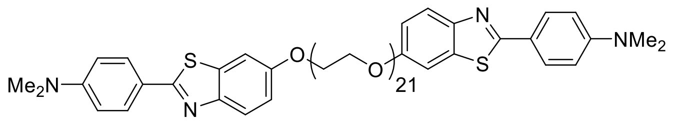

67 % yield. ^{1}H NMR ($CDCl_3$, 400 MHz) δ (ppm): 7.90 (d, *J* = 8.9 Hz, 4H), 7.85 (d, *J* = 8.9 Hz, 2H), 7.34 (d, *J* = 2.5 Hz, 2H), 7.05 (dd, *J* = 8.9, 2.5 Hz, 2H), 6.74 (d, *J* = 9.0 Hz, 4H), 4.23 – 4.16 (m, 4H), 3.85 (t, *J* = 4.8 Hz, 4H), 3.89 (dd, *J* = 5.7, 4.1 Hz, 4H), 3.63 (m, 71H), 3.05 (s, 9H). ^{13}C NMR ($CDCl_3$, 100.6 MHz) δ (ppm): 166.7, 156.4, 152.0, 149.1, 135.8, 128.7, 122.8, 121.7, 115.6, 111.9, 105.5, 71.0, 70.8, 70.7, 69.9, 68.3, 40.3. ESI-HRMS(+) m/z (%): calc. $C_{72}H_{111}N_4O_{22}S_2$ 1447.7126; exp. 1447.7100 $[M+H]^+$; IR (ATR, cm^{-1}): 2957, 2930, 2861, 1722, 1606, 1460, 1409, 1381, 1268, 1250, 1115, 1102, 1019, 954, 876, 807, 730, 479, 446, 438.

DimerPEG200 (Compound 23)

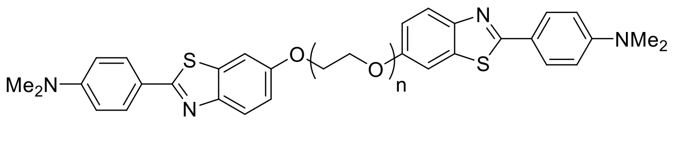

87%. ^{1}H NMR ($CDCl_3$, 400 MHz) δ (ppm): 7.95 – 7.78 (m, 6H), 7.38 – 7.29 (m, 2H), 7.11 – 7.00 (m, 2H), 6.72 (dd, *J* = 9.1, 2.2 Hz, 4H), 4.28 – 4.10 (m, 4H), 4.06 – 3.83 (m, 4H), 3.83 – 3.61 (m, 5H), 3.04 (s, 12H). ^{13}C NMR ($CDCl_3$, 100.6 MHz) δ (ppm): 166.7, 156.4, 152.0, 149.3, 149.2, 135.8, 128.6, 122.8, 121.7, 115.6, 111.8, 105.7, 105.5, 105.5, 71.1, 71.0, 70.8, 70.1, 70.0, 69.9, 68.4, 68.3, 40.3. IR (ATR, cm^{-1}): 3005, 2918, 1659, 1607, 1491, 1437, 1408, 1315, 1225, 1019, 952, 819, 705, 669, 636, 605, 554, 520, 434.

DimerPEG400 (Compound 24)

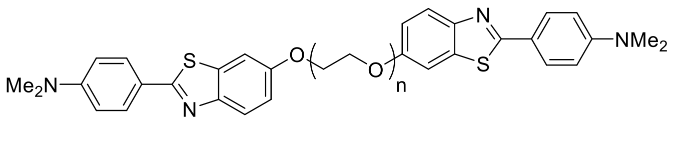

49%. ^{1}H NMR ($CDCl_3$, 400 MHz) δ (ppm): 7.89 (d, *J* = 8.6 Hz, 4H), 7.84 (d, *J* = 8.9 Hz, 2H), 7.32 (d, *J* = 2.6 Hz, 2H), 7.04 (dd, *J* = 8.9, 2.5 Hz, 2H), 6.72 (d, *J* = 8.5 Hz, 4H), 4.17 (t, *J* = 5.6 Hz, 4H), 3.87 (t, *J* = 5.2 Hz, 4H), 3.78 – 3.70 (m, 4H), 3.70 – 3.52 (m,~25H), 3.03 (s, 12H). ^{13}C NMR ($CDCl_3$, 100.6 MHz) δ (ppm): 166.5, 156.2, 151.9, 149.0, 135.7, 128.5, 122.6, 121.6, 115.4, 111.7, 105.4, 70.8, 70.6, 70.5, 69.7, 68.1, 40.1. IR (ATR, cm^{-1}): 2873, 1607, 1560, 1532, 1492, 1448, 1364, 1314, 1286, 1225, 1190, 1169, 1121, 1068, 1007, 943, 819, 688, 518.

DimerPEG1000 (Compound 25)

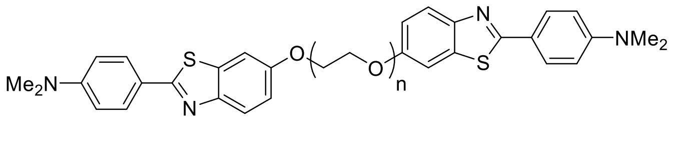

56 %. ^{1}H NMR ($CDCl_3$, 400 MHz) δ (ppm): 7.89 (d, *J* = 8.9 Hz, 4H), 7.84 (d, *J* = 8.9 Hz, 2H), 7.33 (d, *J* = 2.6 Hz, 2H), 7.05 (dd, *J* = 8.9, 2.5 Hz, 2H), 6.73 (d, *J* = 9.0 Hz, 4H), 4.26 – 4.12 (m, 4H), 3.89 (dd, *J* = 5.6, 4.0 Hz, 4H), 3.74 (dd, *J* = 5.8, 3.2 Hz, 4H), 3.64 (d, *J* = 9.7 Hz, 67H), 3.04 (s, 12H). ^{13}C NMR ($CDCl_3$, 100.6 MHz) δ (ppm): 166.6, 156.4, 152.0, 149.2, 135.8, 128.6, 122.8, 121.7, 115.6, 111.8, 105.5, 71.0, 70.7, 70.7, 69.9, 68.2, 40.3. IR (ATR, cm^{-1}): 2872, 1606, 1492, 1451, 1350, 1291, 1255, 1173, 1104, 1018, 972, 919, 848, 808, 799, 530, 515, 473, 453.

DimerPEG1500 (Compound 26)

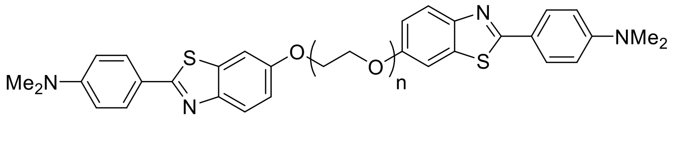

57%. ^{1}H NMR ($CDCl_3$, 400 MHz) δ (ppm): 7.90 (d, *J* = 8.9 Hz, 4H), 7.84 (d, *J* = 8.9 Hz, 2H), 7.34 (d, *J* = 2.6 Hz, 2H), 7.05 (dd, *J* = 8.9, 2.5 Hz, 2H), 6.74 (d, *J* = 9.0 Hz, 4H), 4.26 – 4.14 (m, 4H), 3.96 – 3.85 (m, 4H), 3.44 (m, 104H), 3.05 (s, 12H). ^{13}C NMR ($CDCl_3$, 100.6 MHz) δ (ppm): 166.5, 156.2, 151.9, 149.0, 135.7, 128.5, 122.7, 121.6, 115.4, 111.7, 105.4, 70.8, 70.6, 70.6, 70.5, 70.5, 69.7, 68.1, 40.2. IR (ATR, cm^{-1}): 2924, 2869, 1606, 1559, 1531, 1491, 1447, 1364, 1313, 1285, 1263, 1223, 1190, 1170, 1127, 1067, 942, 819, 687, 589.

DimerPEG2000 (Compound 27)

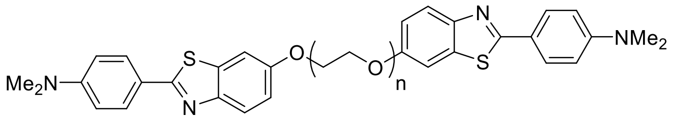

59%. ^{1}H NMR ($CDCl_3$, 400 MHz) δ (ppm): 7.90 (d, *J* = 8.9 Hz, 4H), 7.84 (d, *J* = 8.9 Hz, 2H), 7.34 (d, *J* = 2.6 Hz, 2H), 7.05 (dd, *J* = 8.9, 2.5 Hz, 2H), 6.74 (d, *J* = 9.0 Hz, 4H), 4.26 – 4.14 (m, 4H), 3.96 – 3.85 (m, 4H), 3.44 (m, 104H), 3.05 (s, 12H). ^{13}C NMR ($CDCl_3$, 100.6 MHz) δ (ppm): 166.7, 156.4, 152.0, 149.2, 135.8, 128.6, 122.8, 121.7, 115.6, 111.8, 105.5, 71.0, 70.7, 70.7, 70.6, 69.9, 68.3, 40.3. IR (ATR, cm^{-1}): 2933, 1646, 1494, 1438, 1412, 1387, 1255, 1096, 1062, 723, 659, 577, 556, 545, 495, 483, 465, 407.

2-(2-(2-methoxyethoxy)ethoxy)ethyl methanesulfonate (28)

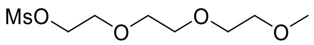

0.151 mL (1.949 mmol) of methanesulfonyl chloride were added dropwise at 0 °C to a solution of 0.191 g (1.218 mmol) of triethylene glycol monomethyl ether and 0.319 mL (1.827 mmol) of DIPEA in 20 mL of anhydrous CH_2Cl_2. The resulting solution was stirred at room temperature 4 hours. After concentration in vacuo, the residue was partitioned between CH_2Cl_2 (10 mL) and H_2O (10 mL) and separated. The aqueous layer was re-extracted with CH_2Cl_2 (4 x 10 mL). The combined organic fractions, were dried over anhydrous Na_2SO_4, filtered, and concentrated. The residue was purified by flash column chromatography (silica, CH_2Cl_2:MeOH (0-10 %). The product was obtained as slightly yellow oil (0.235 g, 80 % yield).

^{1}H NMR ($CDCl_3$, 400 MHz) δ (ppm): 4.40 – 4.33 (m, 2H), 3.78 – 3.73 (m, 2H), 3.70 – 3.59 (m, 6H), 3.55 – 3.50 (m, 2H), 3.36 (s, 3H), 3.06 (s, 3H). ^{13}C NMR ($CDCl_3$, 100.6 MHz) δ (ppm): 72.0, 70.7, 70.6, 70.5, 69.4, 69.1, 59.1, 37.8. ESI-HRMS(+) m/z (%): calc. $C_8H_{18}O_6S$ 242.0722; exp. 242.0714 $[M+Na]^+$. IR (ATR, cm^{-1}): 2875, 1454, 1348, 1247, 1199, 1172, 1101, 1016, 973, 918, 848, 800, 732, 527, 455.

4-(6-(2-(2-(2-methoxyethoxy)ethoxy)ethoxy)benzo[d]thiazol-2-yl)-N,N-dimethylaniline (29)

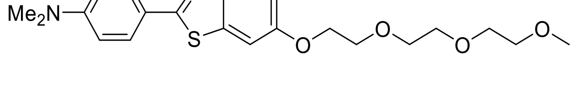

0.050 g (0.206 mmol) of **28** in 2 mL of DMF_{anh} were added to a solution of 0.084 g (0.31 mmol) of **1** and 0.074 g (3.1 mmol) of NaH in 4 mL of anhydrous DMF. The resulting solution was stirred overnight at 90 ºC. Then, the solution was let to cool at room temperature, and 10 mL of aqueous NH_4Cl conc. were added and the solution was stirred 5 minutes. The solution was extracted with CH_2Cl_2 (4 × 5 mL) until no color is observed in the organic layer. The combined organic fractions were dried over anhydrous Na_2SO_4, filtered, and concentrated. The residue was purified by flash column chromatography (silica, CH_2Cl_2:AcOEt (0-100 %). The product was obtained as white solid (0.052 g, 61 % yield).

^{1}H NMR ($CDCl_3$, 400 MHz) δ (ppm): 7.89 (d, *J* = 8.9 Hz, 2H), 7.84 (d, *J* = 8.9 Hz, 1H), 7.32 (d, *J* = 2.5 Hz, 1H), 7.04 (dd, *J* = 8.9, 1H), 6.71 (d, *J* = 9.0 Hz, 2H), 4.21 – 4.13 (m, 2H), 3.91 – 3.83 (m, 2H), 3.78 – 3.61 (m, 6H), 3.57 – 3.50 (m, 2H), 3.37 (s, 3H), 3.02 (s, 6H). ^{13}C NMR ($CDCl_3$, 100.6 MHz) δ (ppm): 166.6, 156.3, 151.9, 149.1, 135.7, 128.5, 122.7, 121.6, 115.5, 111.7, 105.4, 72.0, 70.9, 70.7, 70.6, 69.8, 68.2, 59.1, 40.2. ESI-HRMS(+) m/z (%): calc. $C_{22}H_{28}N_2O_4S$ 417.1848; exp. 417.1837 $[M+H]^+$. IR (ATR, cm^{-1}): 2922, 2884, 1606, 1559, 1492, 1449, 1365, 1349, 1285, 1261, 1223, 1188, 1138, 1126, 1101, 1068, 1043, 955, 942, 819.

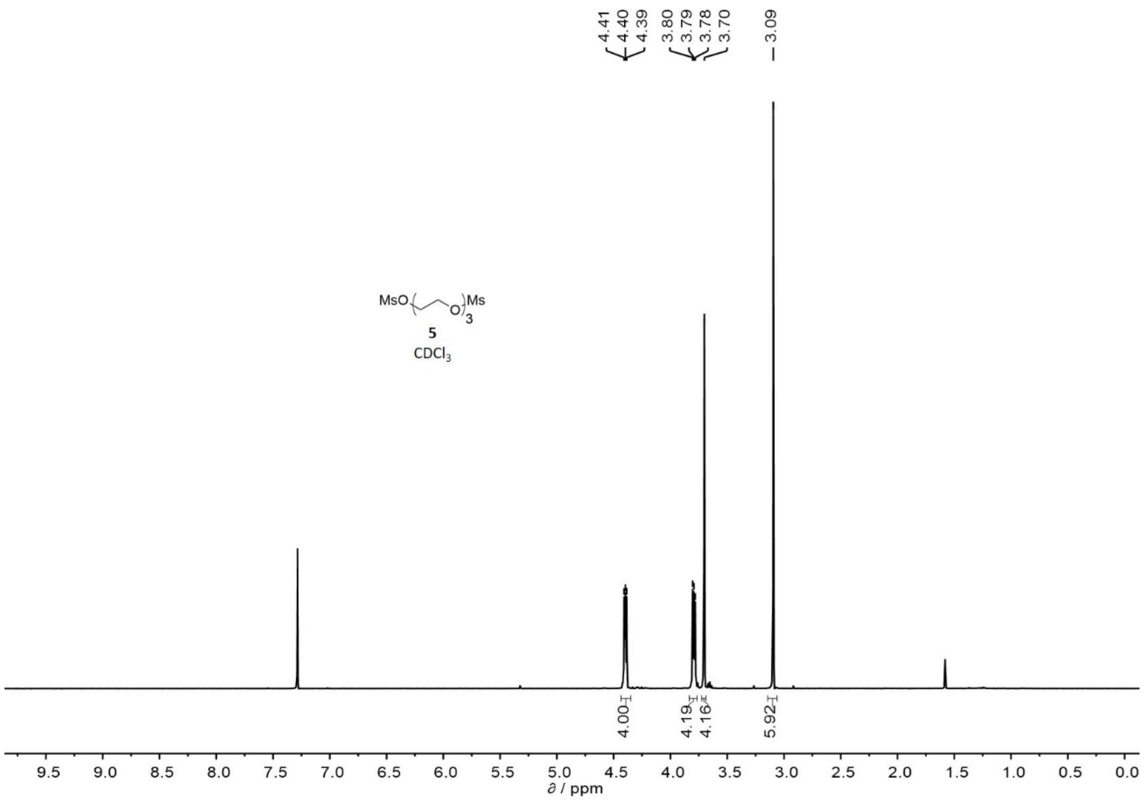
4.41
4.40
4.39
3.80
3.79
3.78
3.70
3.09
MsO O Ms 3
5
CDCl3
4.00
4.19
4.16
5.92
9.5 9.0 8.5 8.0 7.5 7.0 6.5 6.0 5.5 5.0 4.5 4.0 3.5 3.0 2.5 2.0 1.5 1.0 0.5 0.0
∂ / ppm

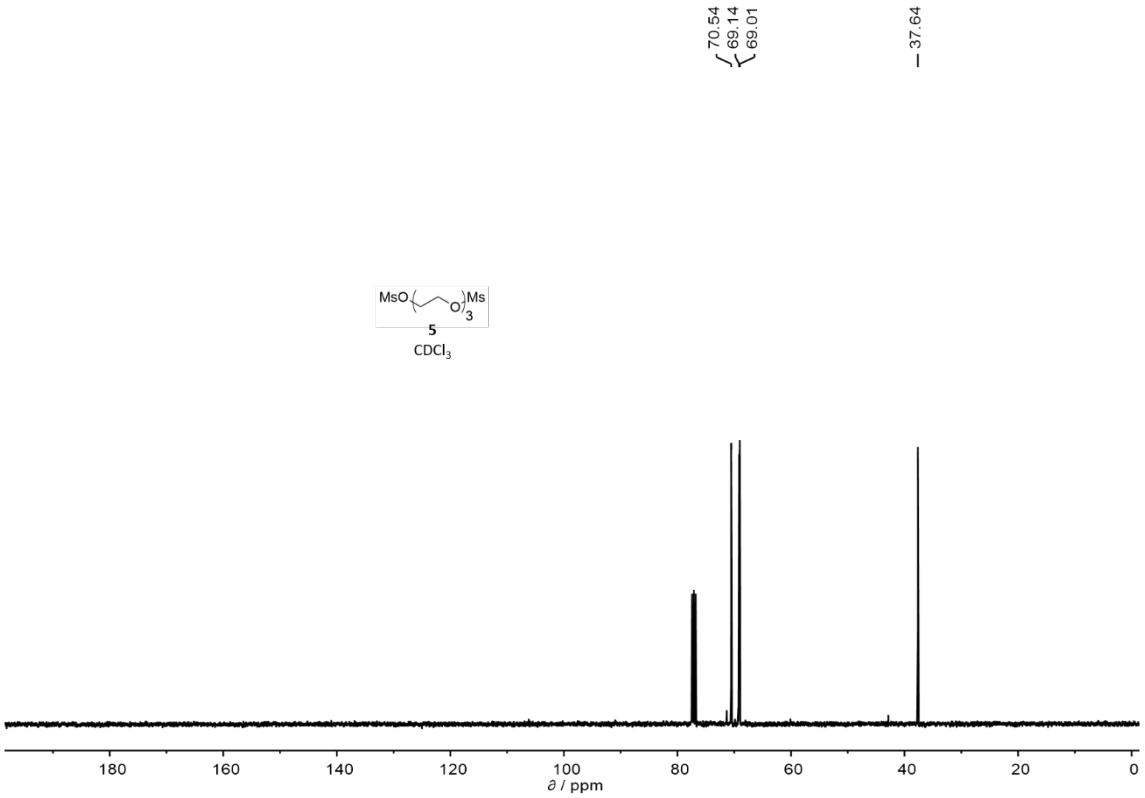
70.54
69.14
69.01
37.64
MsO O Ms 3
5
CDCl3
180 160 140 120 100 80 60 40 20 0
∂ / ppm

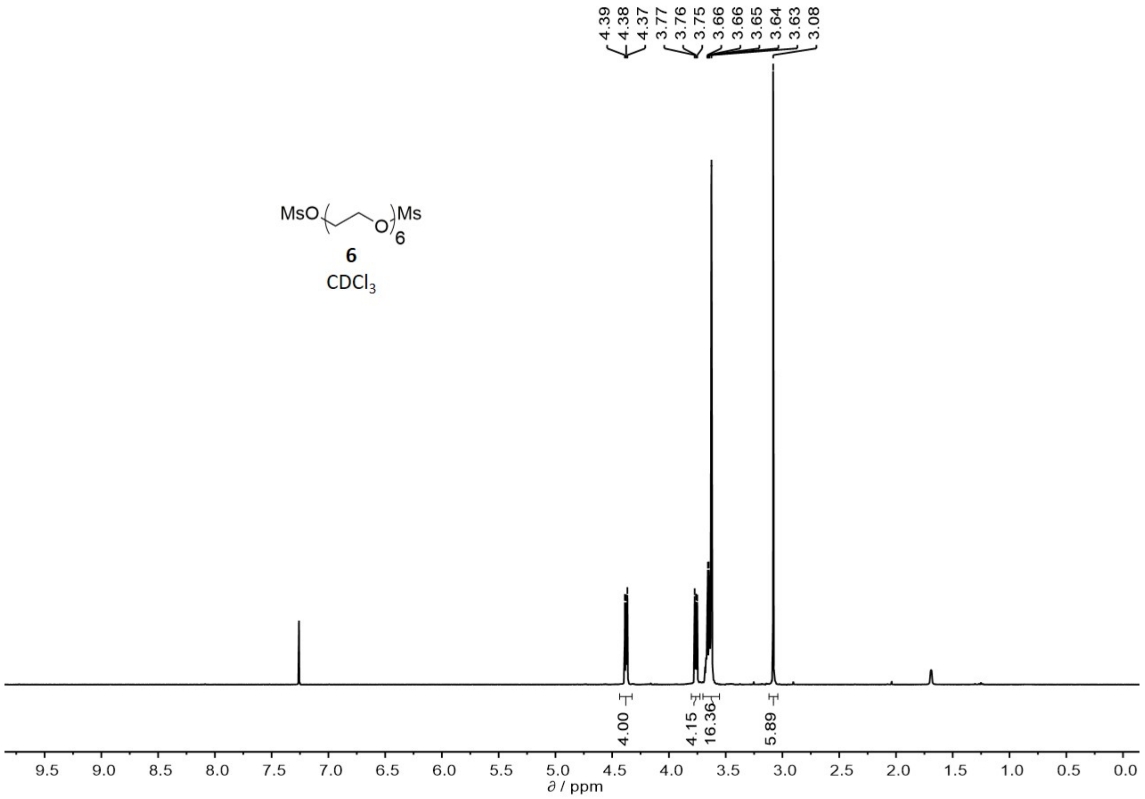
4.39
4.38
4.37
3.77
3.76
3.75
3.66
3.66
3.65
3.64
3.63
3.08
MsO
O
Ms
6
6
CDCl3
4.00
4.15
16.36
5.89
9.5 9.0 8.5 8.0 7.5 7.0 6.5 6.0 5.5 5.0 4.5 4.0 3.5 3.0 2.5 2.0 1.5 1.0 0.5 0.0
∂ / ppm

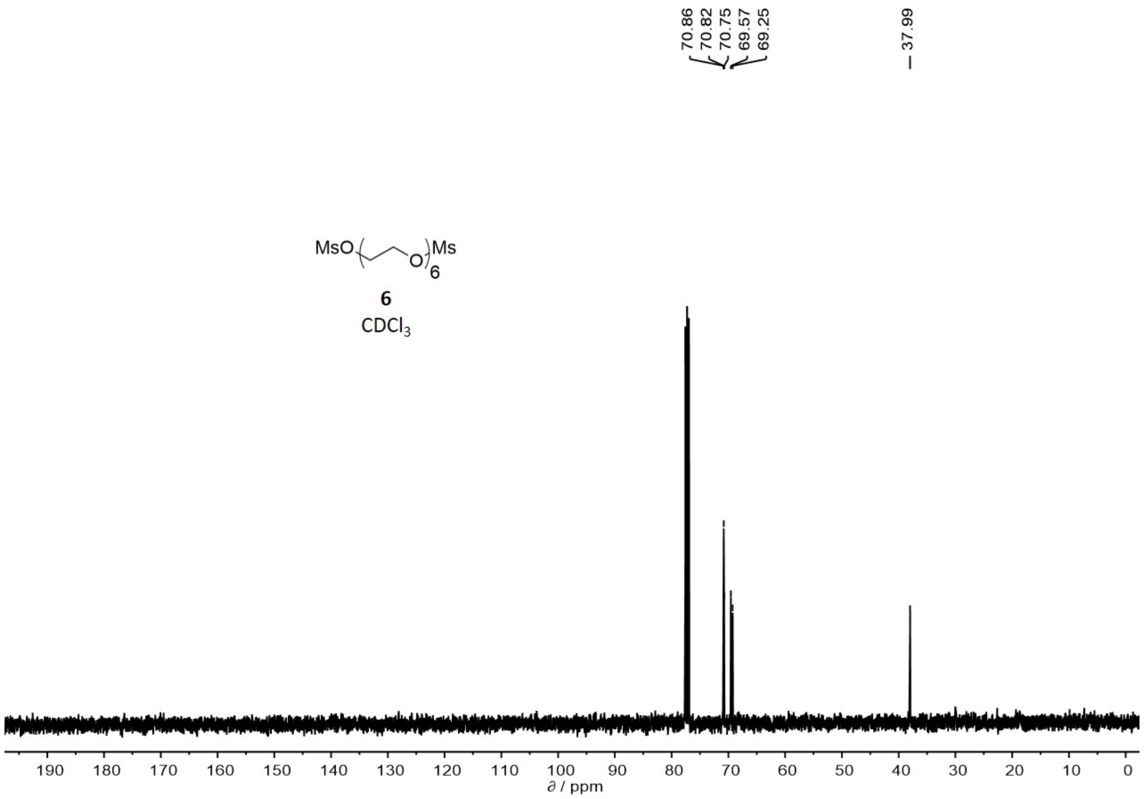
70.86
70.82
70.75
69.57
69.25
37.99
MsO
O
Ms
6
6
CDCl3
190 180 170 160 150 140 130 120 110 100 90 80 70 60 50 40 30 20 10 0
∂ / ppm

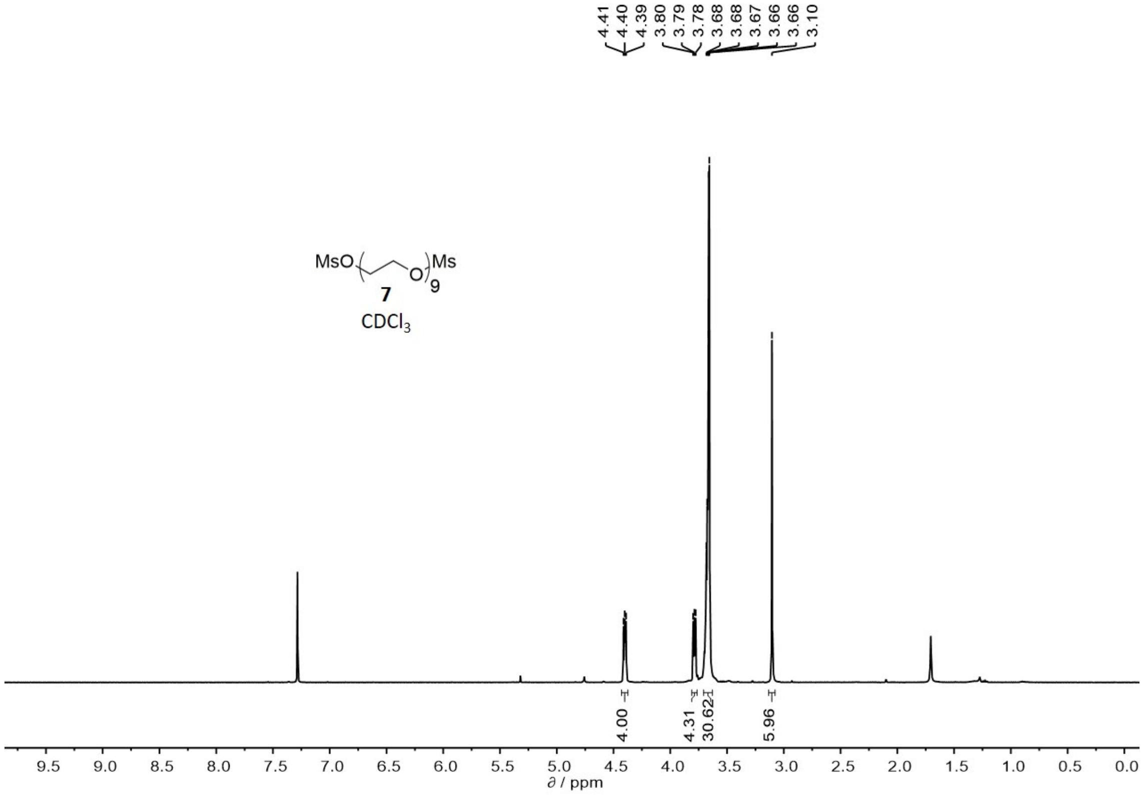
4.41
4.40
4.39
3.80
3.79
3.78
3.68
3.68
3.67
3.66
3.66
3.10
MsO
Ms
9
7
CDCl3
4.00
4.31
30.62
5.96
9.5 9.0 8.5 8.0 7.5 7.0 6.5 6.0 5.5 5.0 4.5 4.0 3.5 3.0 2.5 2.0 1.5 1.0 0.5 0.0
∂ / ppm

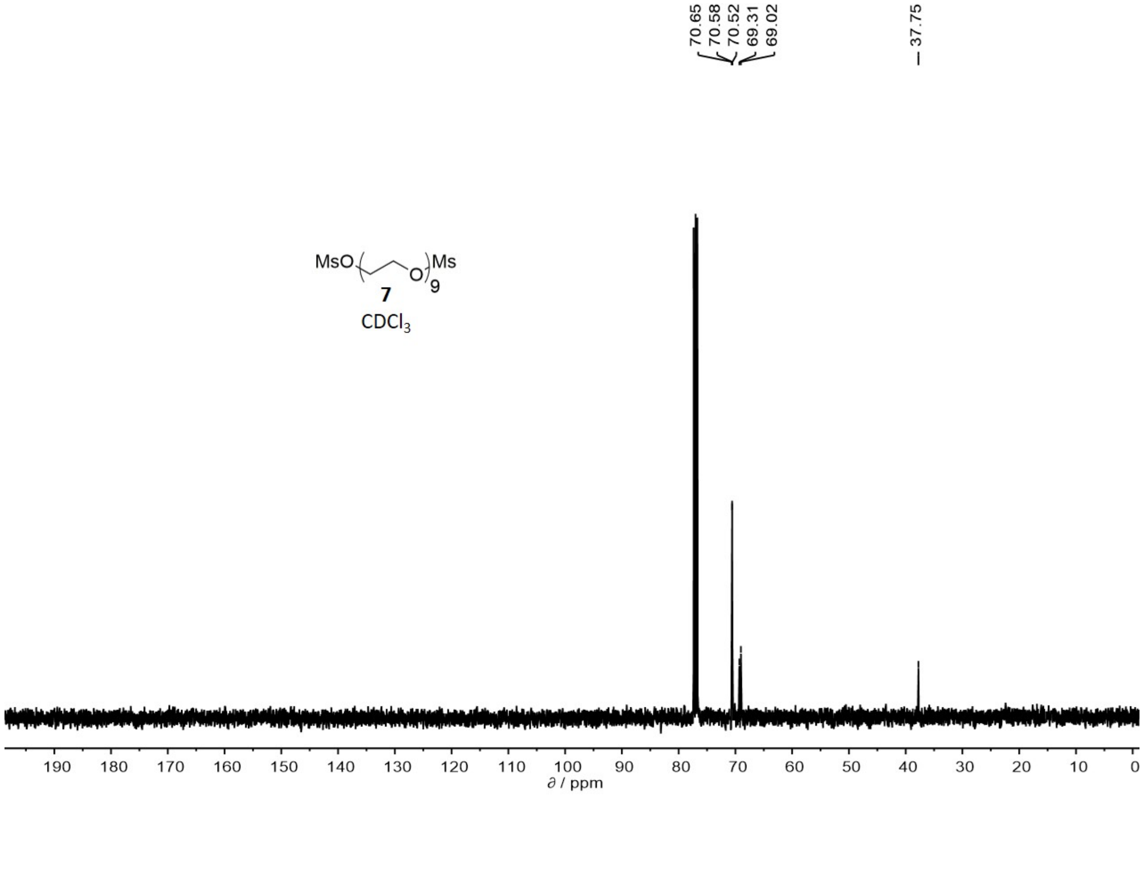
70.65
70.58
70.52
69.31
69.02
37.75
MsO
Ms
9
7
CDCl3
190 180 170 160 150 140 130 120 110 100 90 80 70 60 50 40 30 20 10 0
∂ / ppm

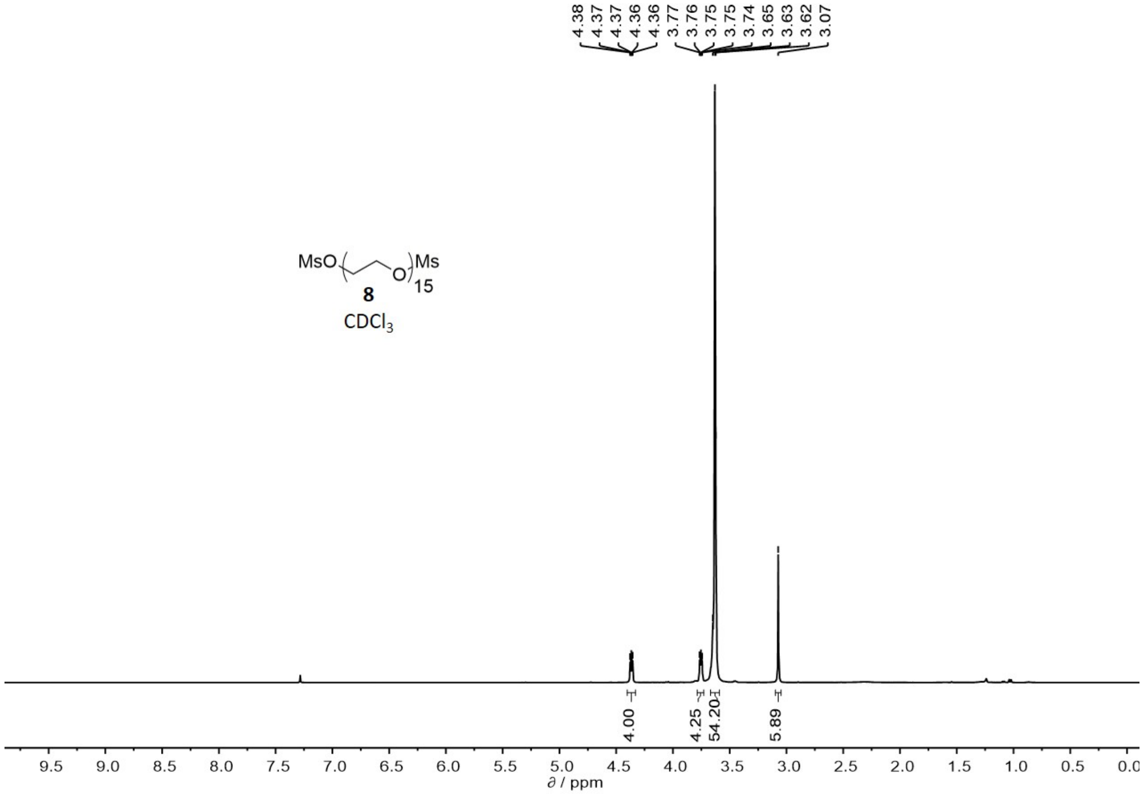
4.38
4.37
4.37
4.36
4.36
3.77
3.76
3.75
3.75
3.74
3.65
3.63
3.62
3.07
MsO
O
Ms
8
15
CDCl3
4.00
4.25
54.20
5.89
9.5
9.0
8.5
8.0
7.5
7.0
6.5
6.0
5.5
5.0
4.5
4.0
3.5
3.0
2.5
2.0
1.5
1.0
0.5
0.0
∂ / ppm

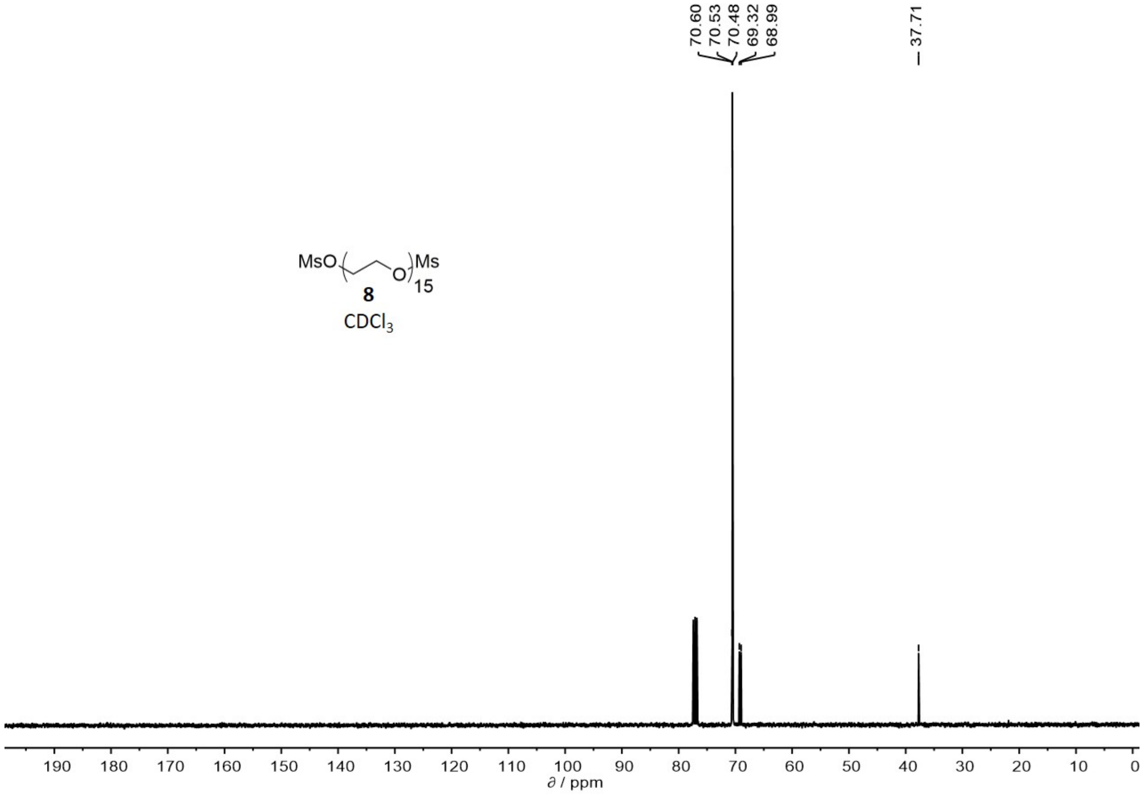
70.60
70.53
70.48
69.32
68.99
37.71
MsO
O
Ms
8
15
CDCl3
190
180
170
160
150
140
130
120
110
100
90
80
70
60
50
40
30
20
10
0
∂ / ppm

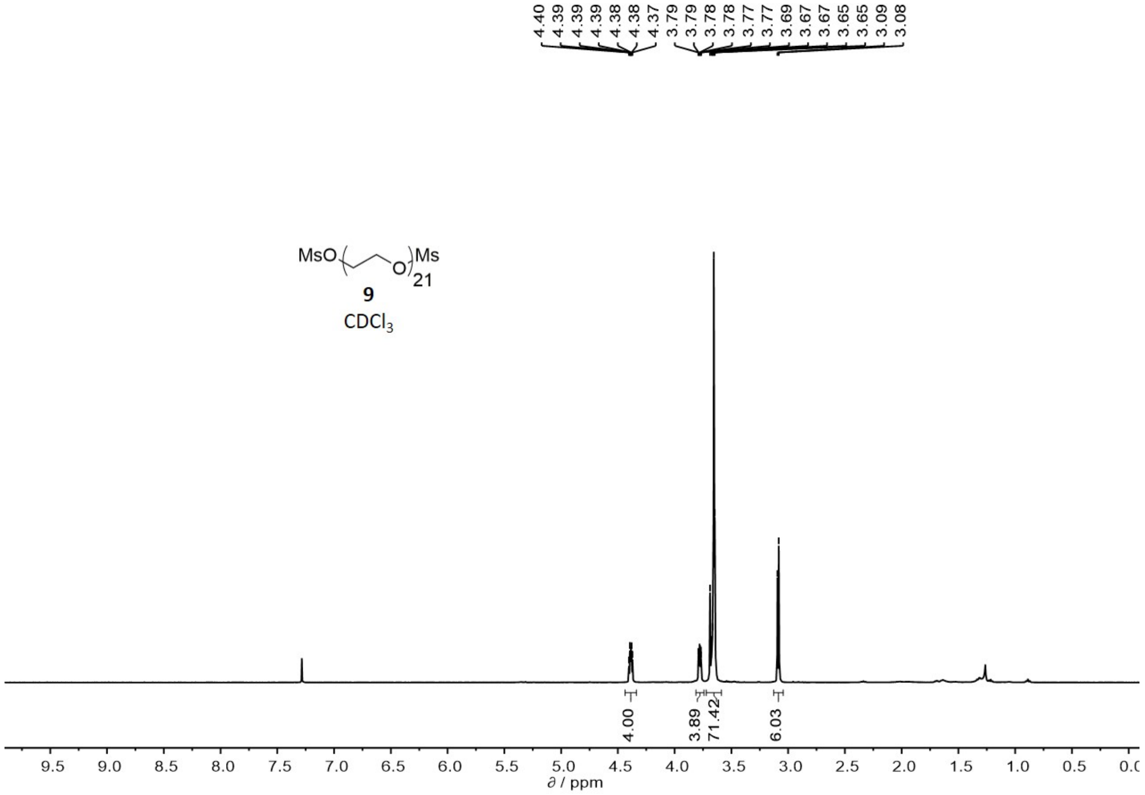
4.40 4.39 4.39 4.39 4.38 4.38 4.37 3.79 3.79 3.78 3.78 3.77 3.77 3.69 3.67 3.67 3.65 3.09 3.08
MsO O Ms 21
9
CDCl3
4.00
3.89
71.42
6.03
9.5 9.0 8.5 8.0 7.5 7.0 6.5 6.0 5.5 5.0 4.5 4.0 3.5 3.0 2.5 2.0 1.5 1.0 0.5 0.0
∂ / ppm

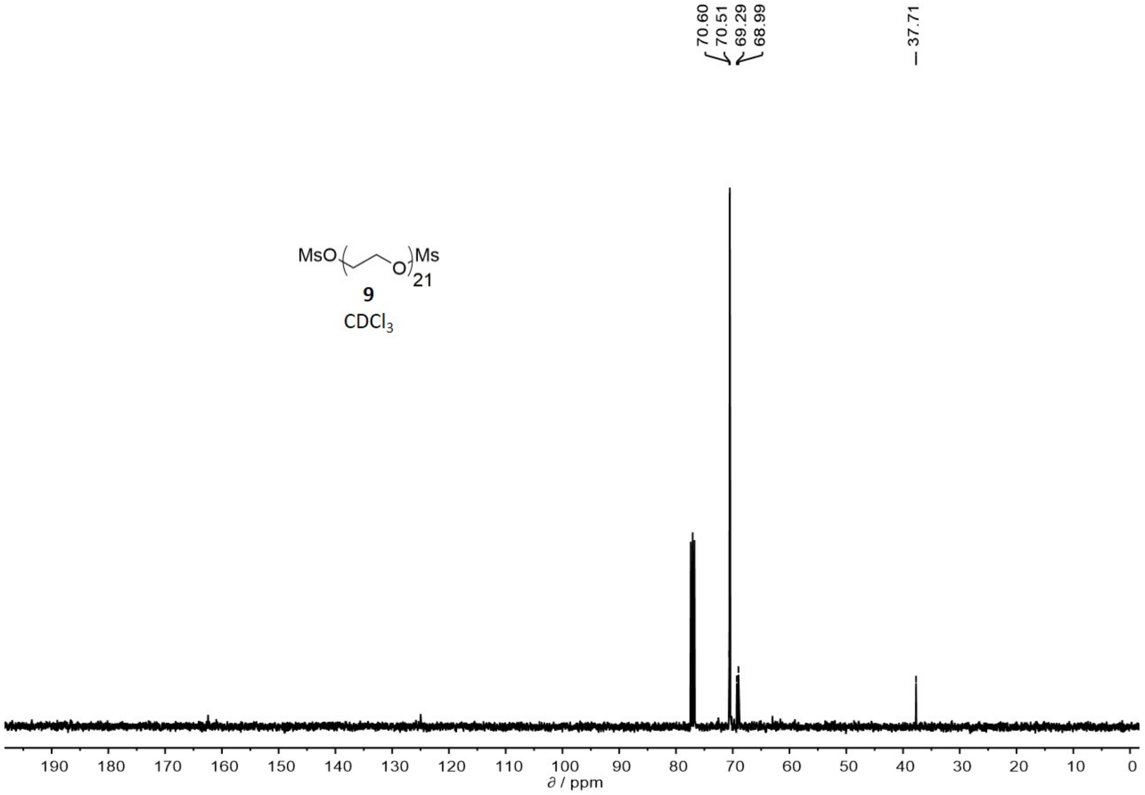
70.60 70.51 69.29 68.99
37.71
MsO O Ms 21
9
CDCl3
190 180 170 160 150 140 130 120 110 100 90 80 70 60 50 40 30 20 10 0
∂ / ppm

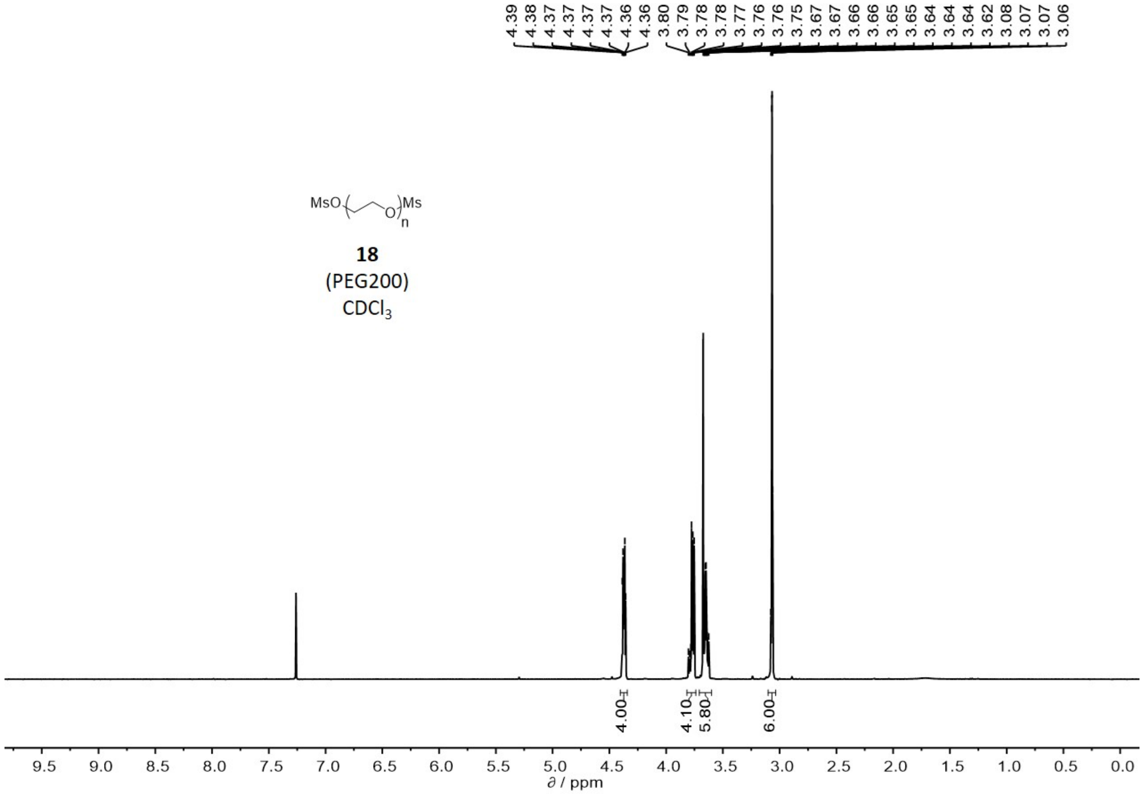
4.39 4.38 4.37 4.37 4.37 4.37 4.36 4.36 3.80 3.79 3.78 3.78 3.77 3.76 3.76 3.75 3.67 3.67 3.66 3.66 3.65 3.65 3.64 3.64 3.64 3.62 3.08 3.07 3.07 3.06
MsO (O) Ms n
18
(PEG200)
$CDCl_3$
4.00
4.10
5.80
6.00
9.5 9.0 8.5 8.0 7.5 7.0 6.5 6.0 5.5 5.0 4.5 4.0 3.5 3.0 2.5 2.0 1.5 1.0 0.5 0.0
∂ / ppm

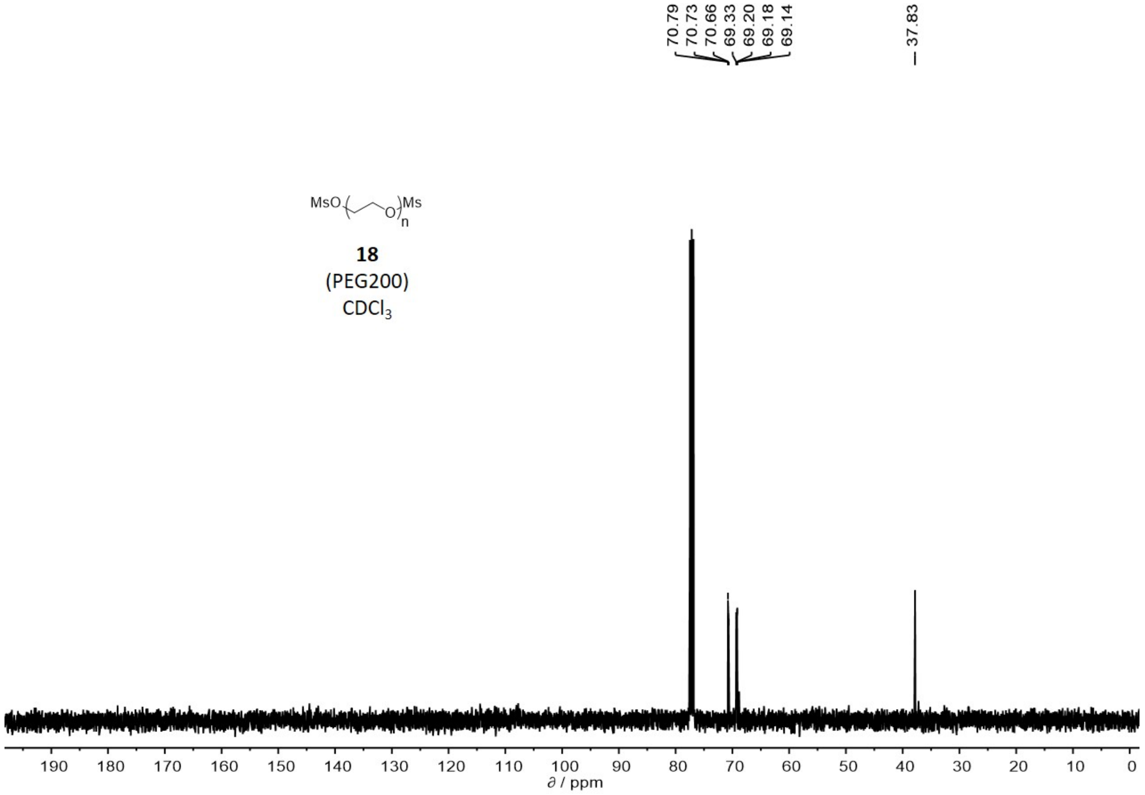
70.79 70.73 70.66 69.33 69.20 69.18 69.14
37.83
MsO (O) Ms n
18
(PEG200)
$CDCl_3$
190 180 170 160 150 140 130 120 110 100 90 80 70 60 50 40 30 20 10 0
∂ / ppm

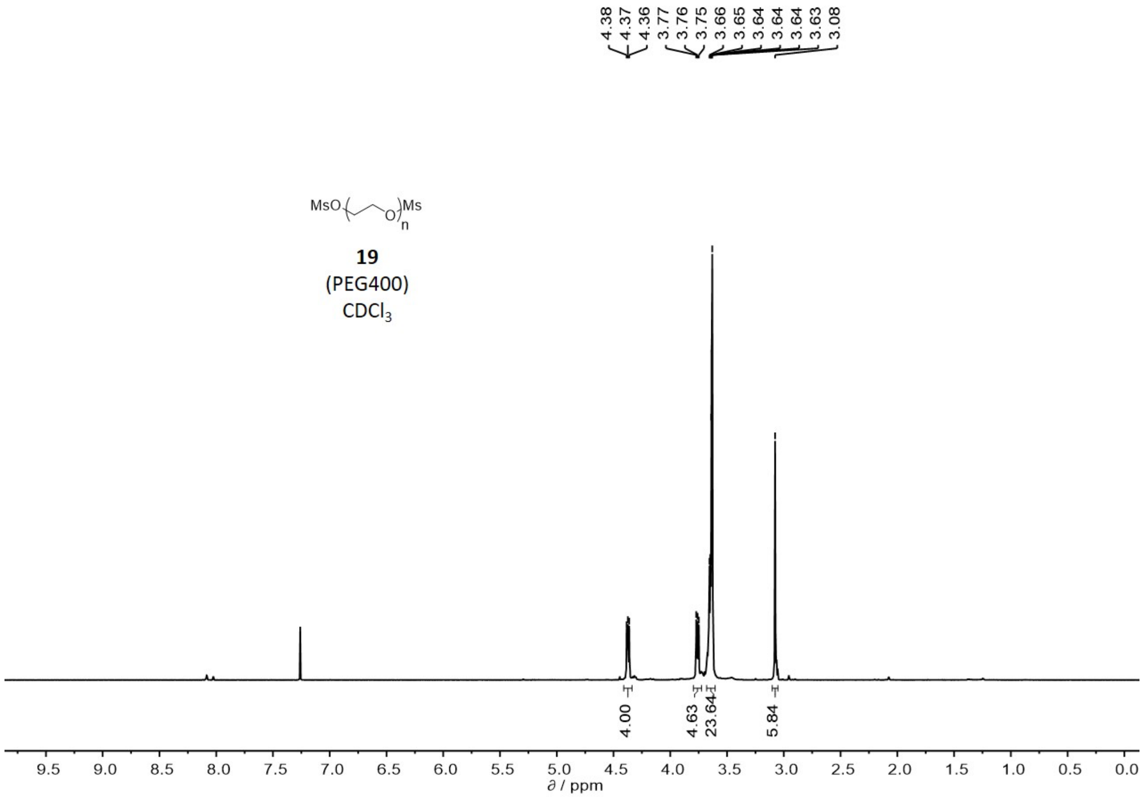
4.38
4.37
4.36
3.77
3.76
3.75
3.66
3.65
3.64
3.64
3.64
3.63
3.08
MsO
O
Ms
n
19
(PEG400)
CDCl3
4.00
4.63
23.64
5.84
9.5 9.0 8.5 8.0 7.5 7.0 6.5 6.0 5.5 5.0 4.5 4.0 3.5 3.0 2.5 2.0 1.5 1.0 0.5 0.0
∂ / ppm

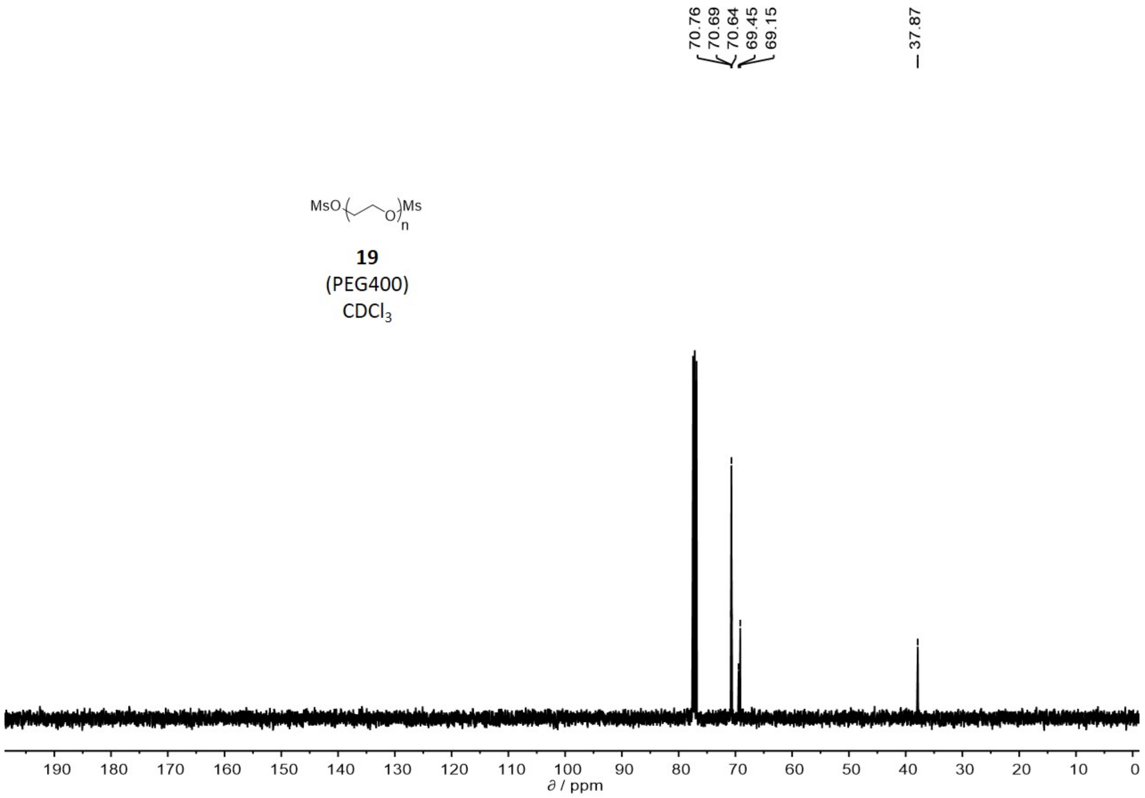
70.76
70.69
70.64
69.45
69.15
37.87
MsO
O
Ms
n
19
(PEG400)
CDCl3
190 180 170 160 150 140 130 120 110 100 90 80 70 60 50 40 30 20 10 0
∂ / ppm

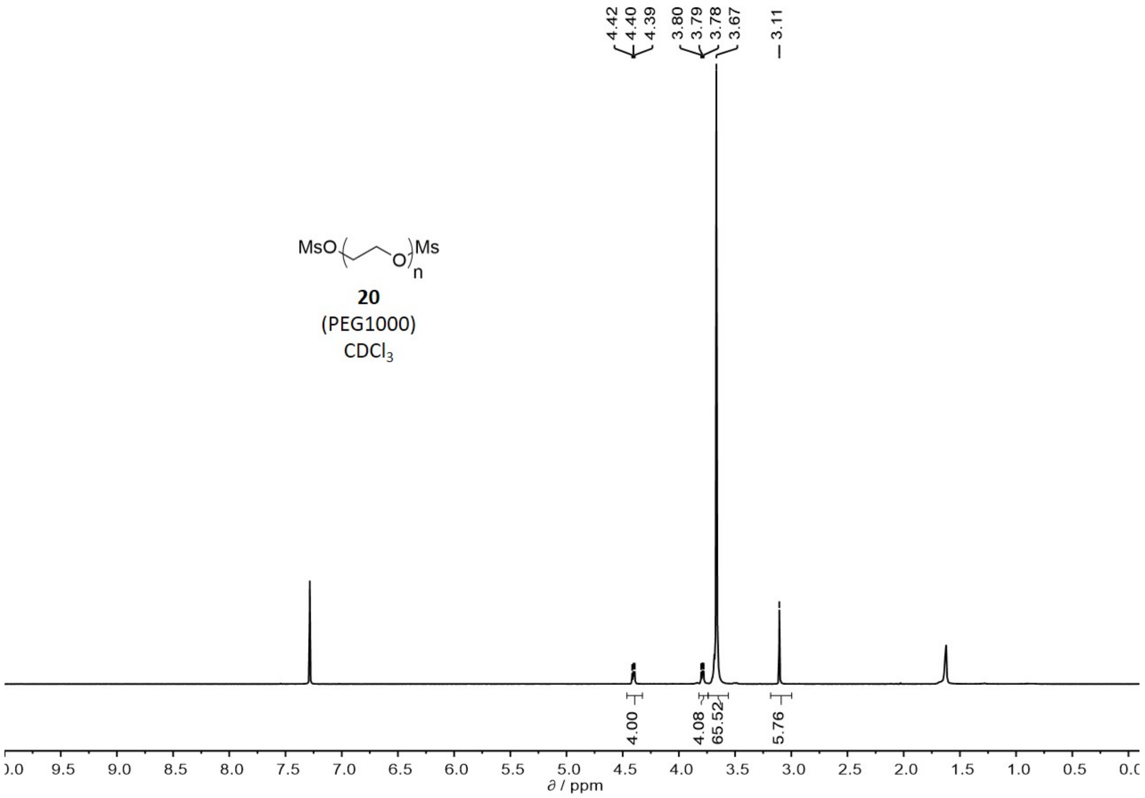
4.42
4.40
4.39
3.80
3.79
3.78
3.67
3.11
MsO O Ms n
20
(PEG1000)
CDCl3
4.00
4.08
65.52
5.76
10.0 9.5 9.0 8.5 8.0 7.5 7.0 6.5 6.0 5.5 5.0 4.5 4.0 3.5 3.0 2.5 2.0 1.5 1.0 0.5 0.0
∂ / ppm

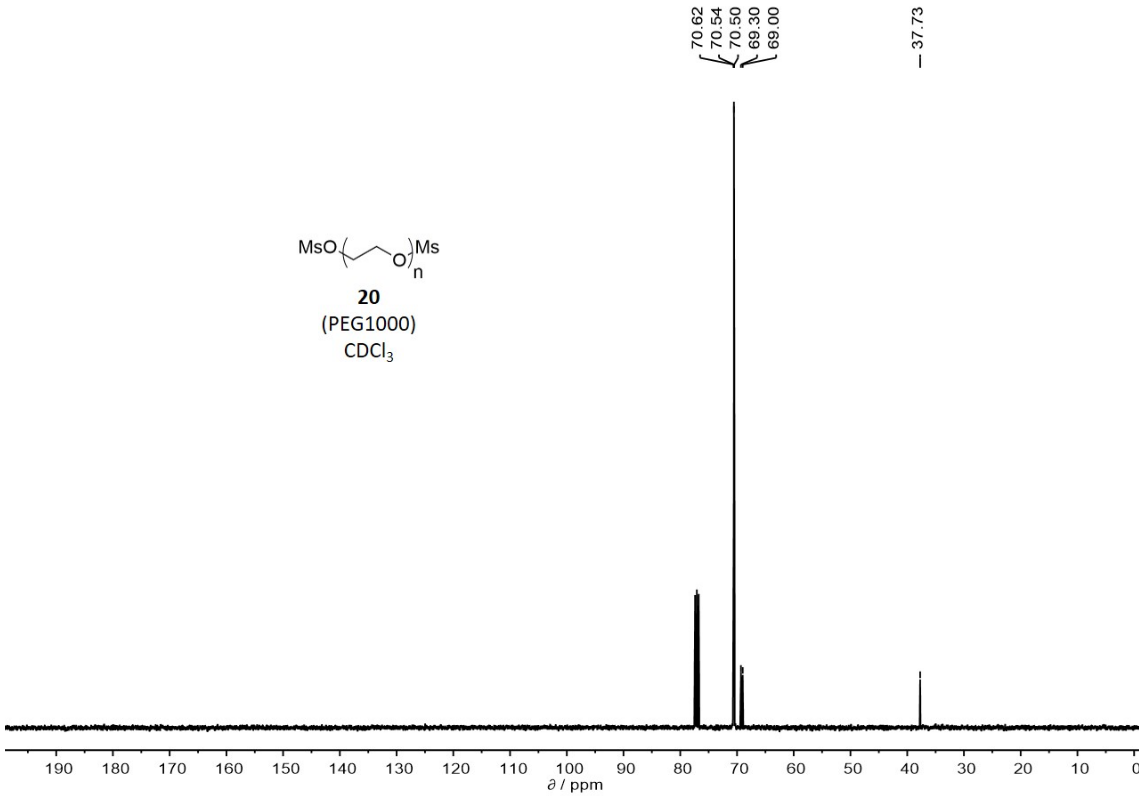
70.62
70.54
70.50
69.30
69.00
37.73
MsO O Ms n
20
(PEG1000)
CDCl3
190 180 170 160 150 140 130 120 110 100 90 80 70 60 50 40 30 20 10 0
∂ / ppm

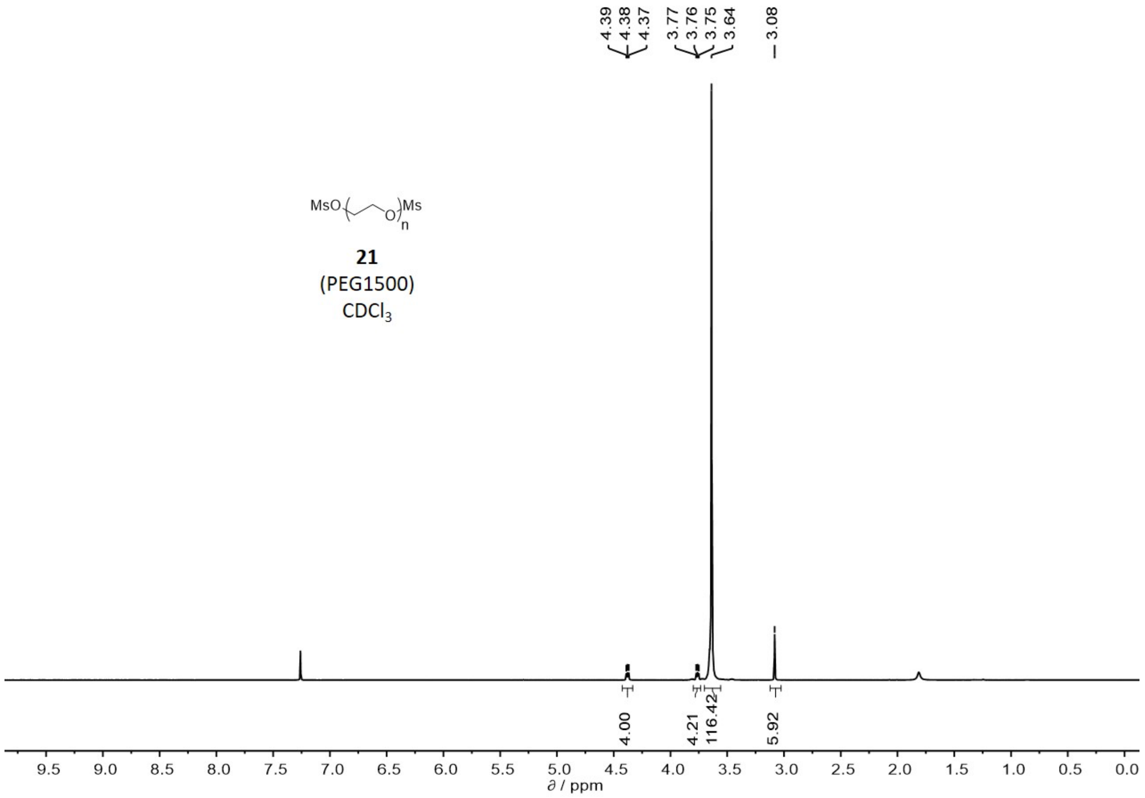
4.39
4.38
4.37
3.77
3.76
3.75
3.64
3.08
MsO
O
Ms
n
21
(PEG1500)
$CDCl_3$
4.00
4.21
116.42
5.92
9.5 9.0 8.5 8.0 7.5 7.0 6.5 6.0 5.5 5.0 4.5 4.0 3.5 3.0 2.5 2.0 1.5 1.0 0.5 0.0
∂ / ppm

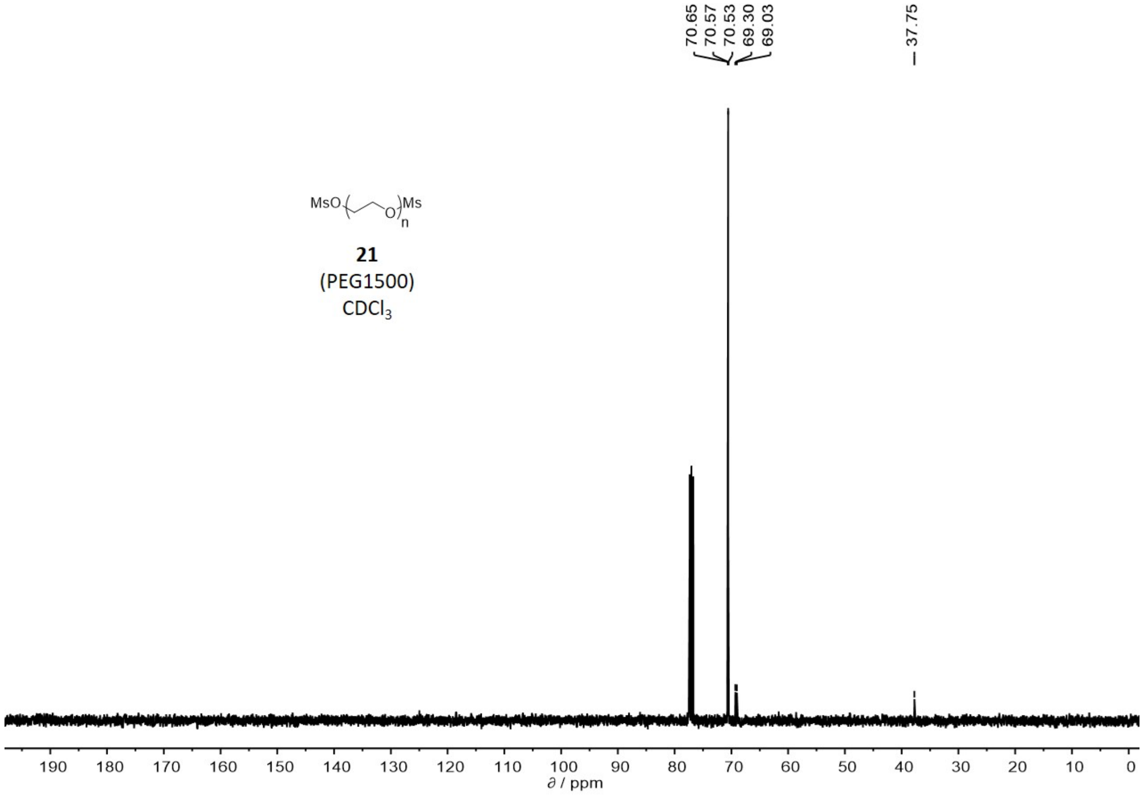
70.65
70.57
70.53
69.30
69.03
37.75
MsO
O
Ms
n
21
(PEG1500)
$CDCl_3$
190 180 170 160 150 140 130 120 110 100 90 80 70 60 50 40 30 20 10 0
∂ / ppm

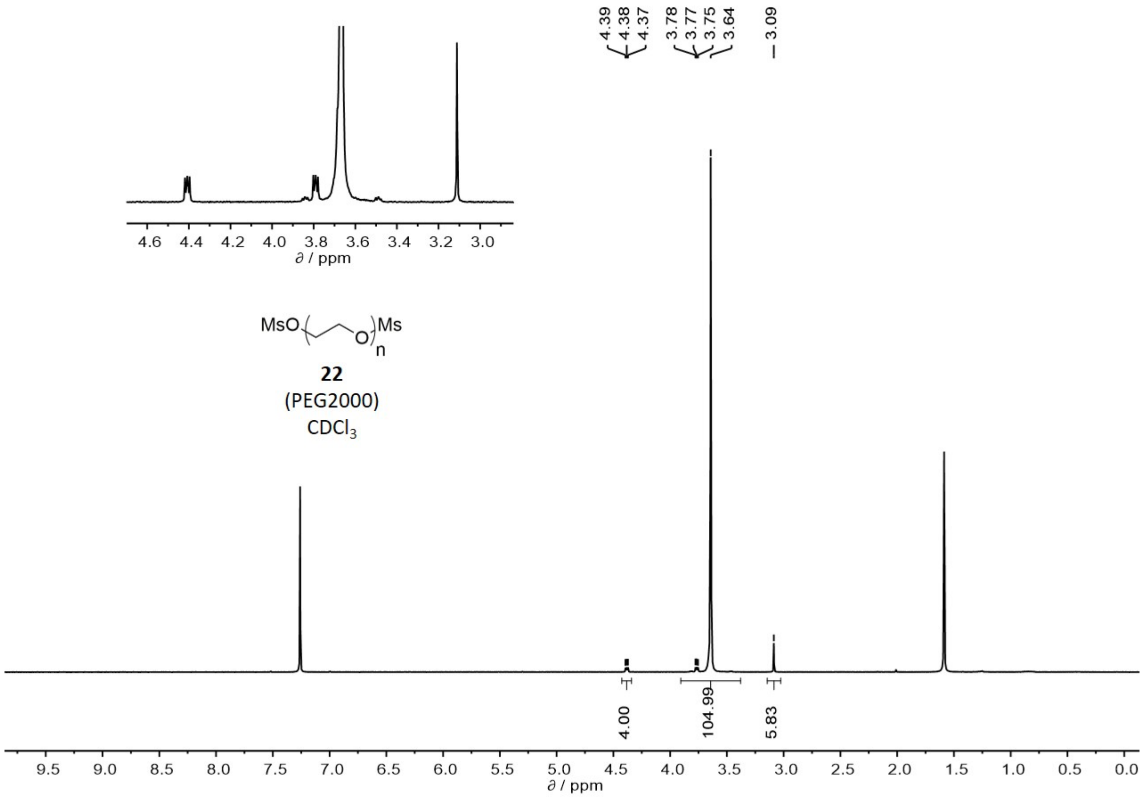
4.39
4.38
4.37
3.78
3.77
3.75
3.64
3.09
MsO(~~O)nMs
22
(PEG2000)
CDCl3
4.00
104.99
5.83
∂ / ppm

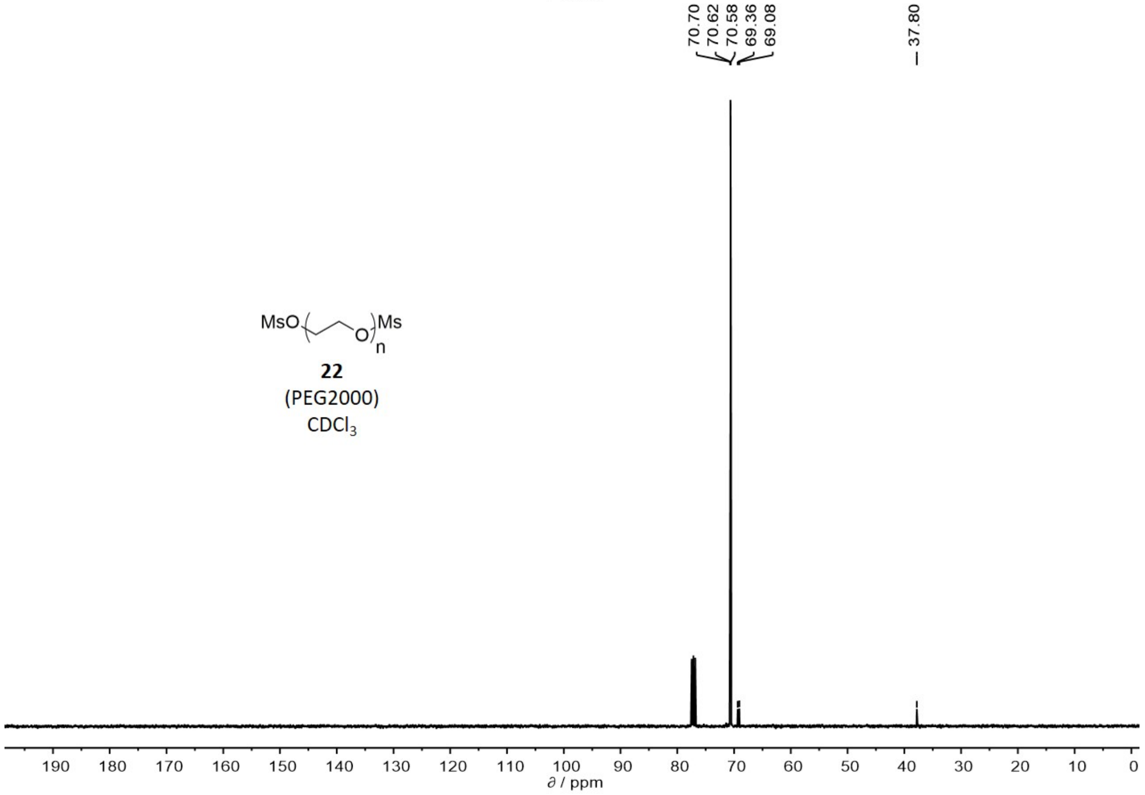
70.70
70.62
70.58
69.36
69.08
37.80
MsO(~~O)nMs
22
(PEG2000)
CDCl3
∂ / ppm

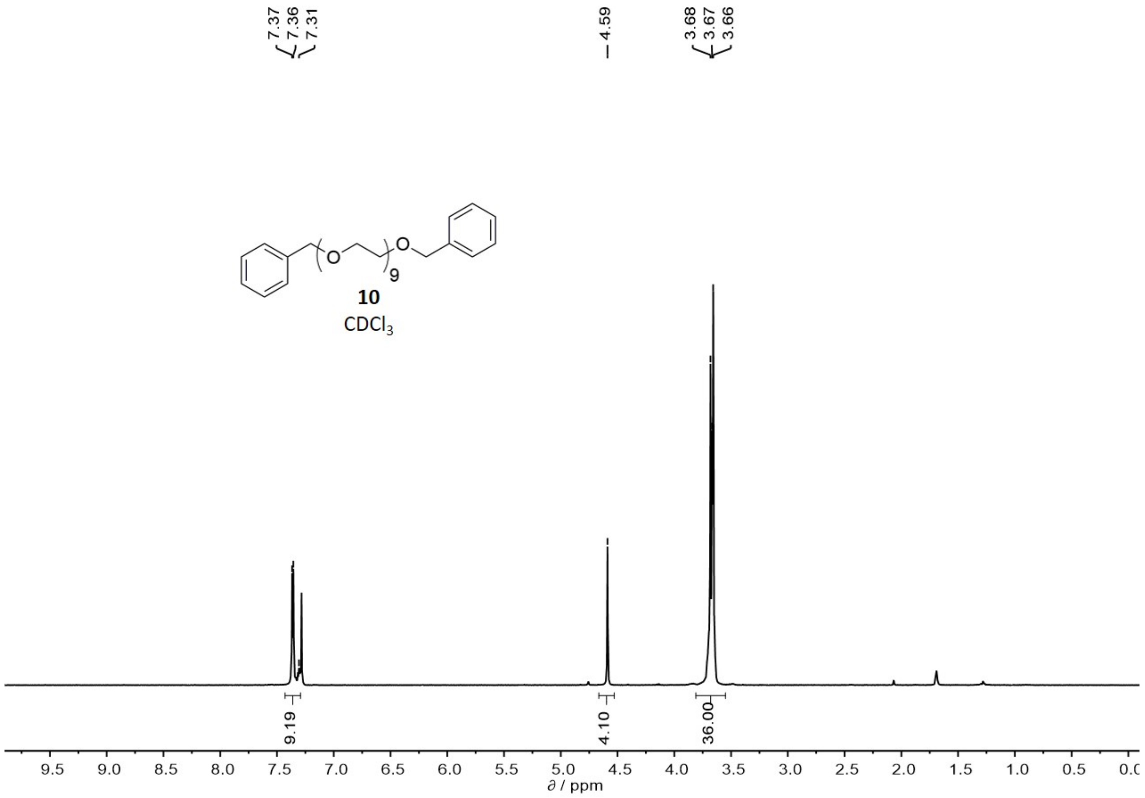
7.37
7.36
7.31
4.59
3.68
3.67
3.66
9
10
CDCl3
9.19
4.10
36.00
9.5 9.0 8.5 8.0 7.5 7.0 6.5 6.0 5.5 5.0 4.5 4.0 3.5 3.0 2.5 2.0 1.5 1.0 0.5
∂ / ppm

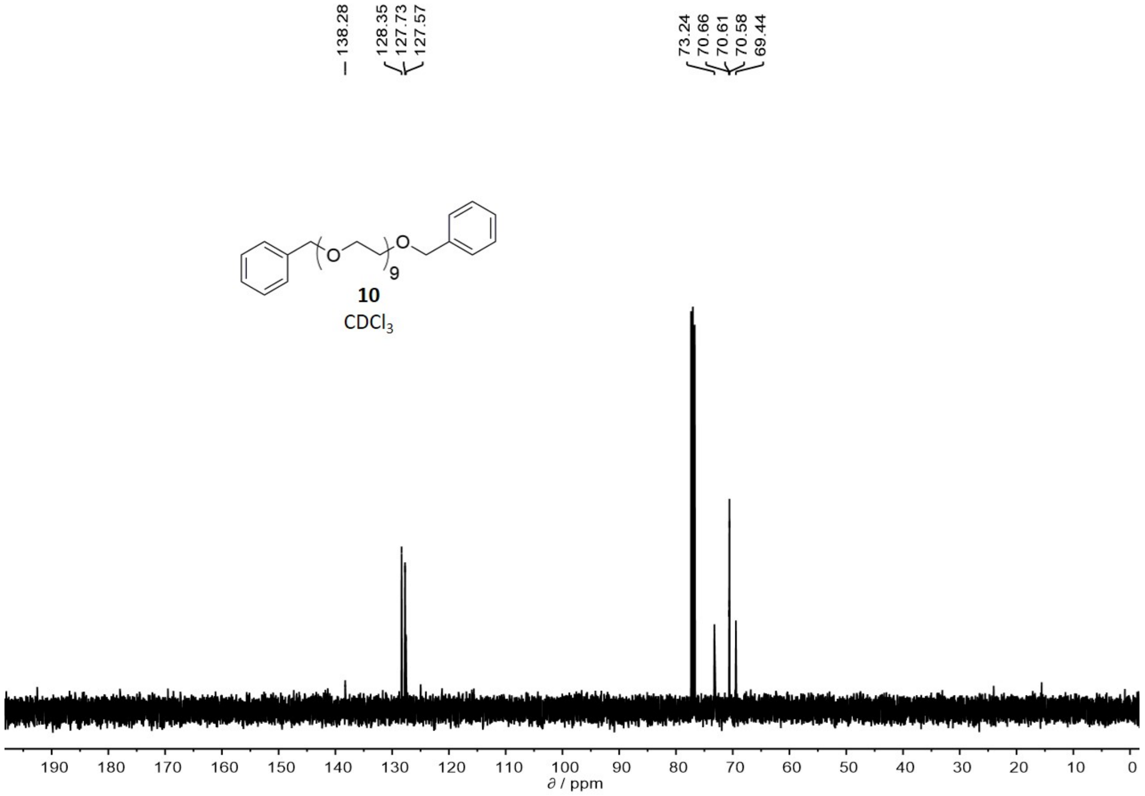
138.28
128.35
127.73
127.57
73.24
70.66
70.61
70.58
69.44
9
10
CDCl3
190 180 170 160 150 140 130 120 110 100 90 80 70 60 50 40 30 20 10 0
∂ / ppm

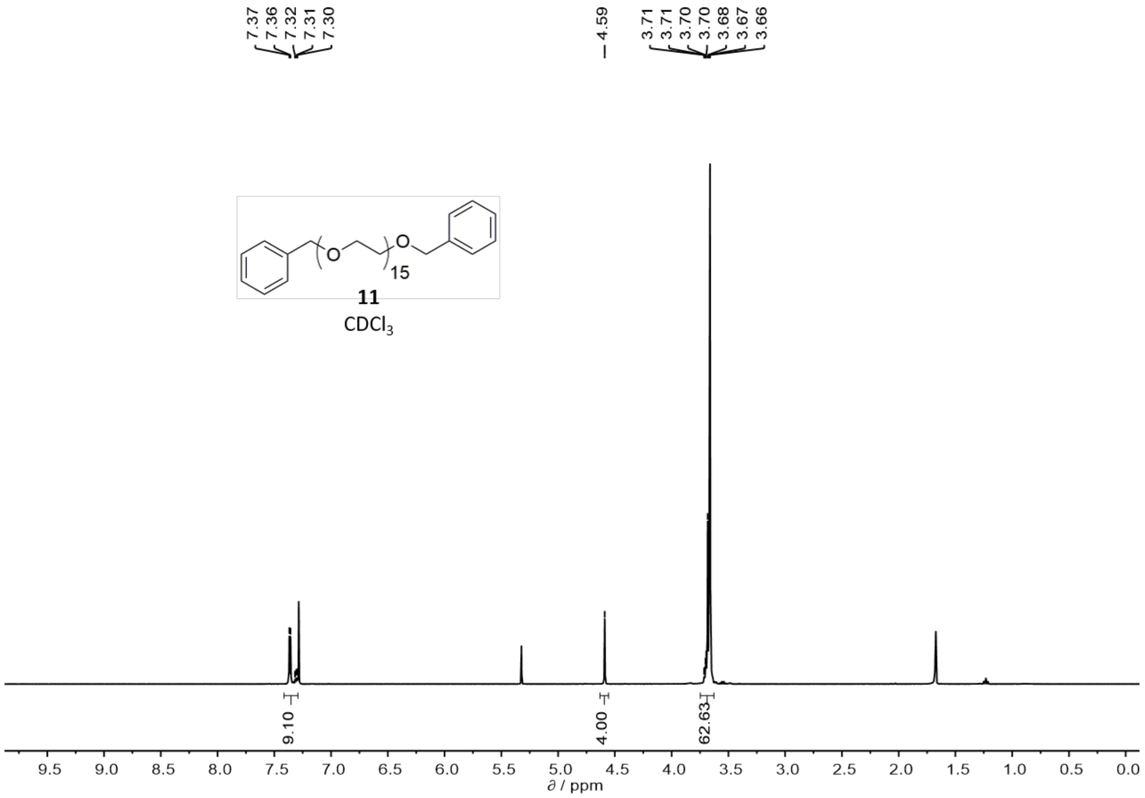
7.37
7.36
7.32
7.31
7.30
4.59
3.71
3.71
3.70
3.70
3.68
3.67
3.66
15
11
CDCl3
9.10
4.00
62.63
9.5 9.0 8.5 8.0 7.5 7.0 6.5 6.0 5.5 5.0 4.5 4.0 3.5 3.0 2.5 2.0 1.5 1.0 0.5 0.0
∂ / ppm

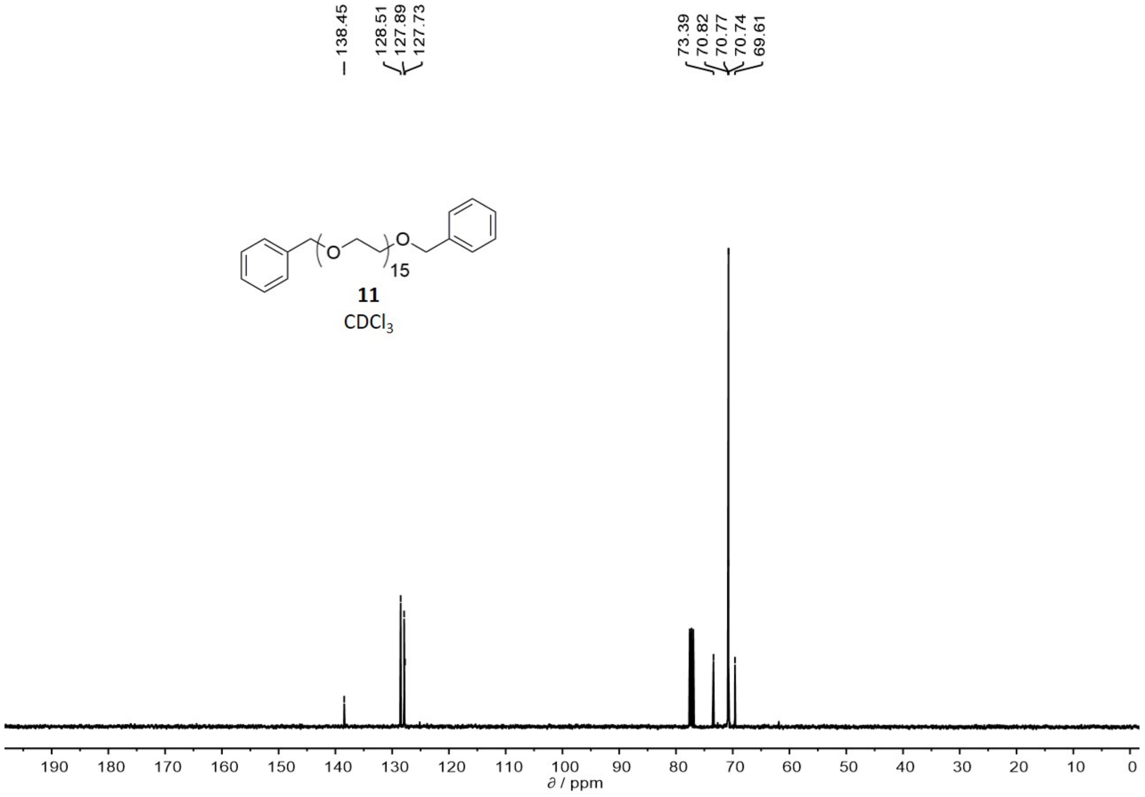
138.45
128.51
127.89
127.73
73.39
70.82
70.77
70.74
69.61
15
11
CDCl3
190 180 170 160 150 140 130 120 110 100 90 80 70 60 50 40 30 20 10 0
∂ / ppm

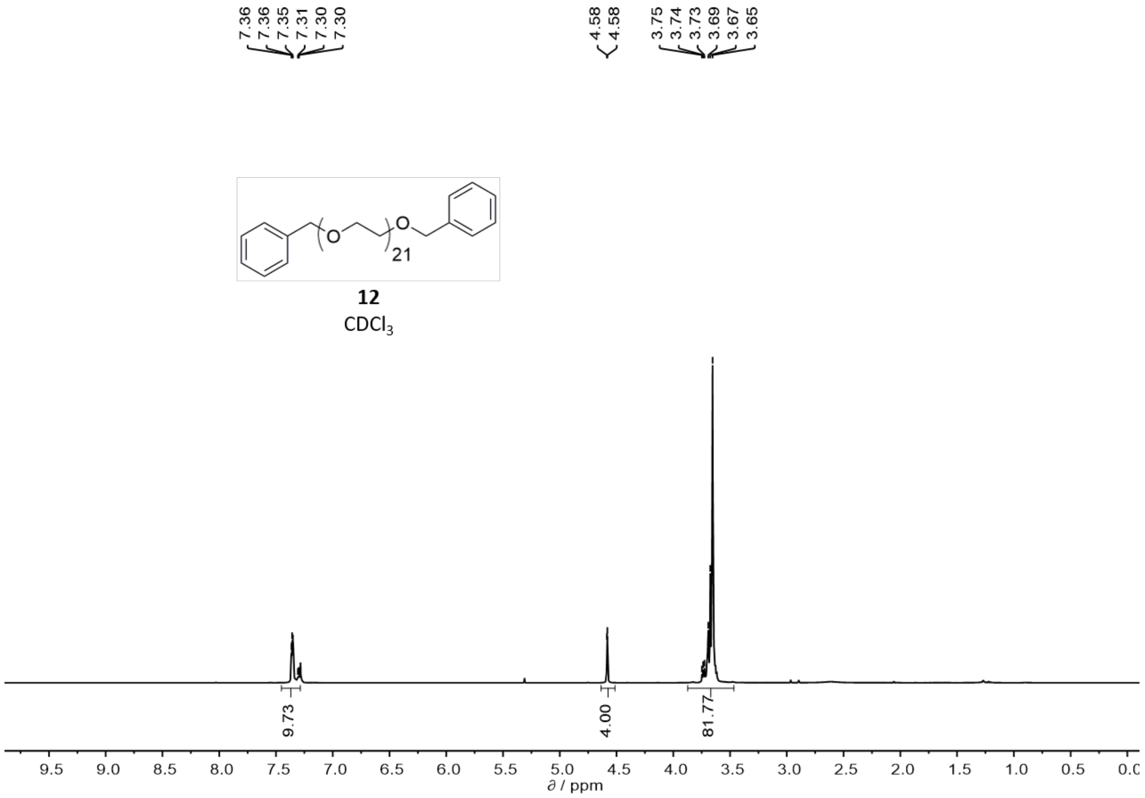
7.36
7.36
7.35
7.31
7.30
7.30
4.58
4.58
3.75
3.74
3.73
3.69
3.67
3.65
21
12
CDCl3
9.73
4.00
81.77
9.5 9.0 8.5 8.0 7.5 7.0 6.5 6.0 5.5 5.0 4.5 4.0 3.5 3.0 2.5 2.0 1.5 1.0 0.5 0.0
∂ / ppm

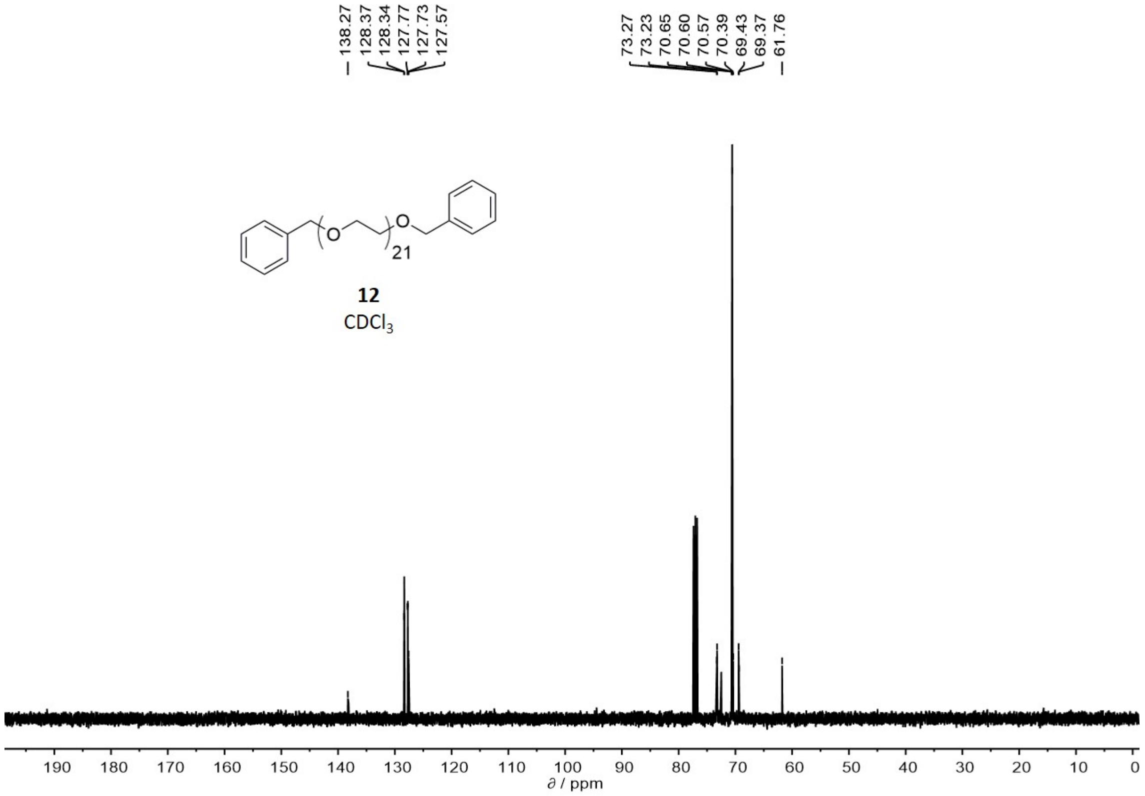
138.27
128.37
128.34
127.77
127.73
127.57
73.27
73.23
70.65
70.60
70.57
70.39
69.43
69.37
61.76
21
12
CDCl3
190 180 170 160 150 140 130 120 110 100 90 80 70 60 50 40 30 20 10 0
∂ / ppm

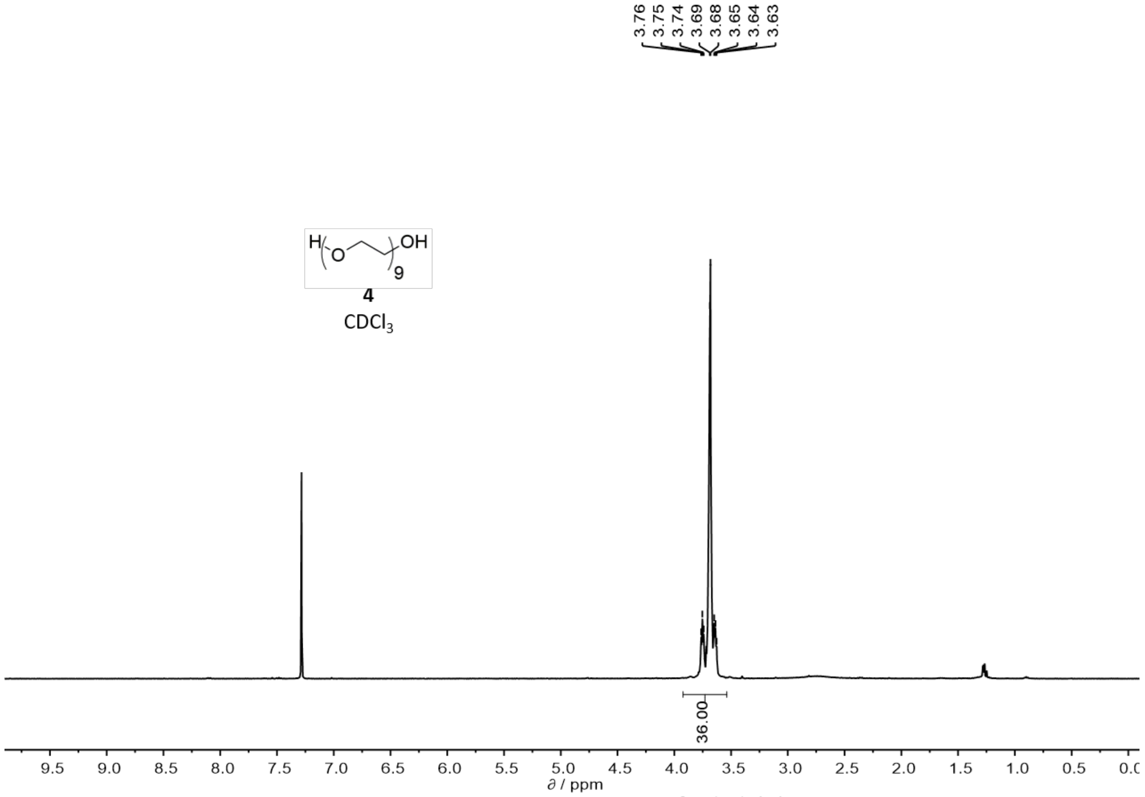
3.76
3.75
3.74
3.69
3.68
3.65
3.64
3.63
H O OH 9
4
$CDCl_3$
36.00
9.5 9.0 8.5 8.0 7.5 7.0 6.5 6.0 5.5 5.0 4.5 4.0 3.5 3.0 2.5 2.0 1.5 1.0 0.5 0.0
∂ / ppm

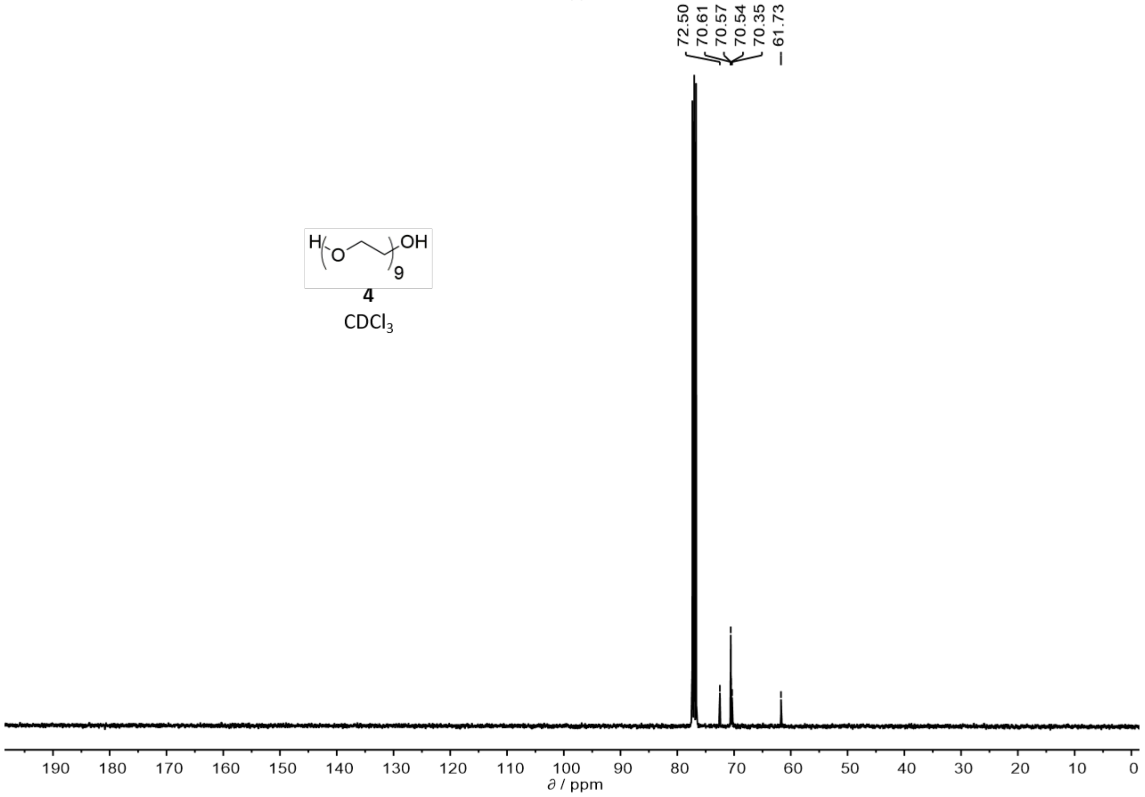
72.50
70.61
70.57
70.54
70.35
61.73
H O OH 9
4
$CDCl_3$
190 180 170 160 150 140 130 120 110 100 90 80 70 60 50 40 30 20 10 0
∂ / ppm

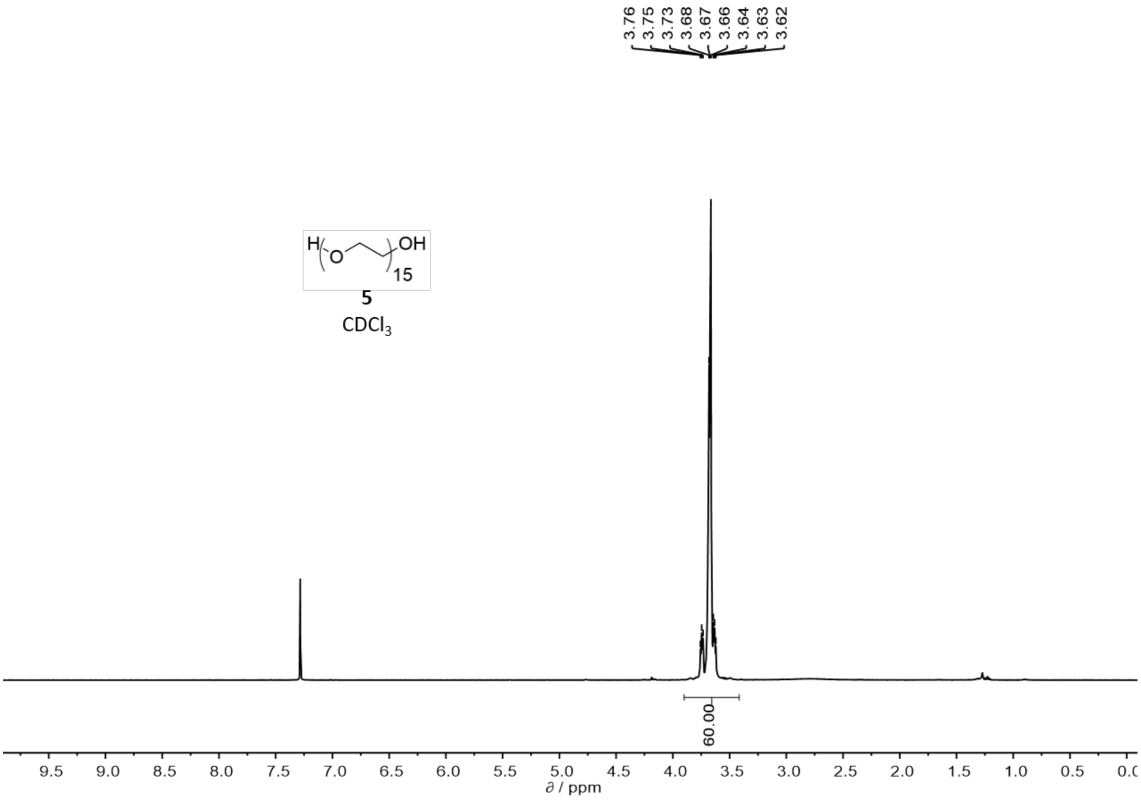
3.76
3.75
3.73
3.68
3.67
3.66
3.64
3.63
3.62
5
CDCl3
60.00
9.5 9.0 8.5 8.0 7.5 7.0 6.5 6.0 5.5 5.0 4.5 4.0 3.5 3.0 2.5 2.0 1.5 1.0 0.5
∂ / ppm

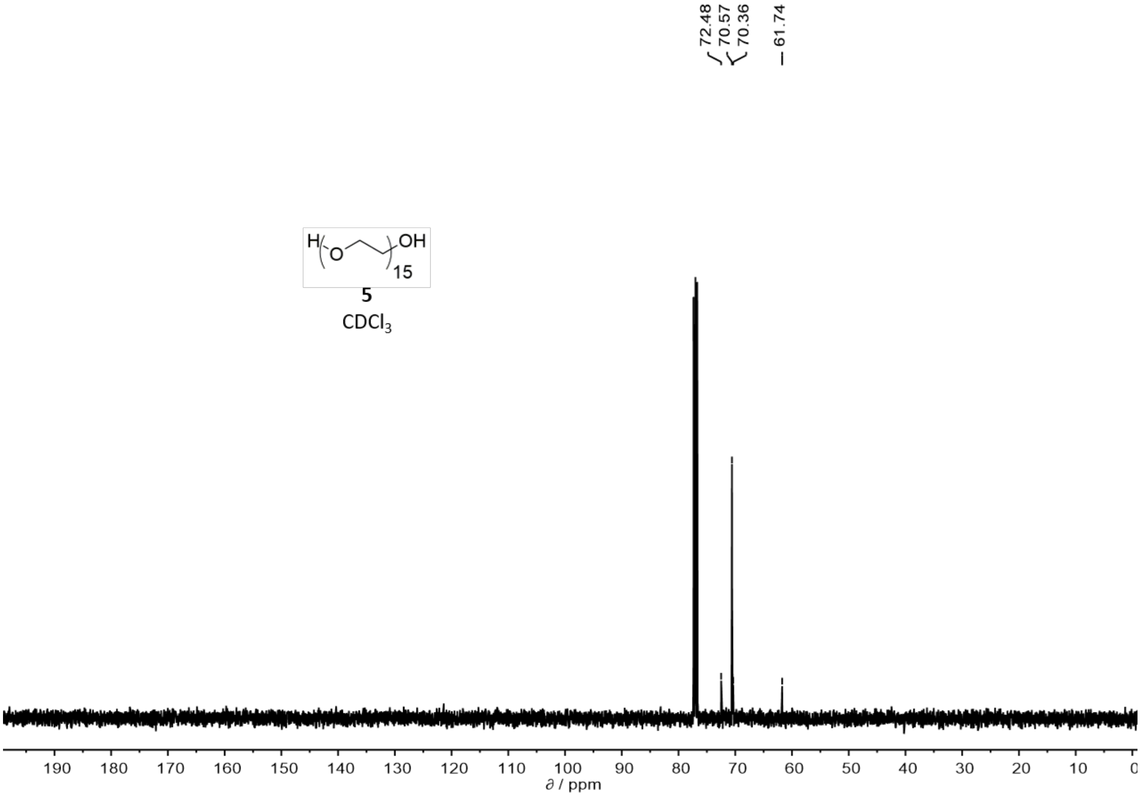
72.48
70.57
70.36
61.74
5
CDCl3
190 180 170 160 150 140 130 120 110 100 90 80 70 60 50 40 30 20 10
∂ / ppm

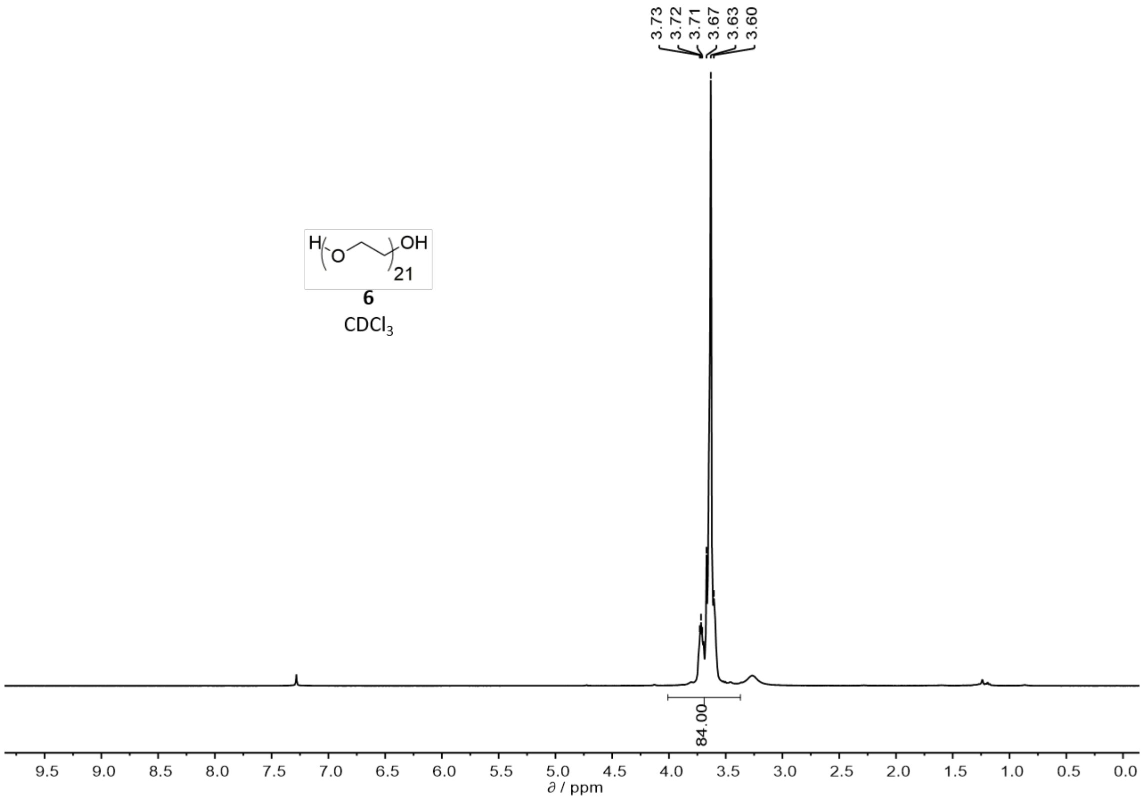
3.73
3.72
3.71
3.67
3.63
3.60
6
CDCl3
84.00
9.5 9.0 8.5 8.0 7.5 7.0 6.5 6.0 5.5 5.0 4.5 4.0 3.5 3.0 2.5 2.0 1.5 1.0 0.5 0.0
∂ / ppm

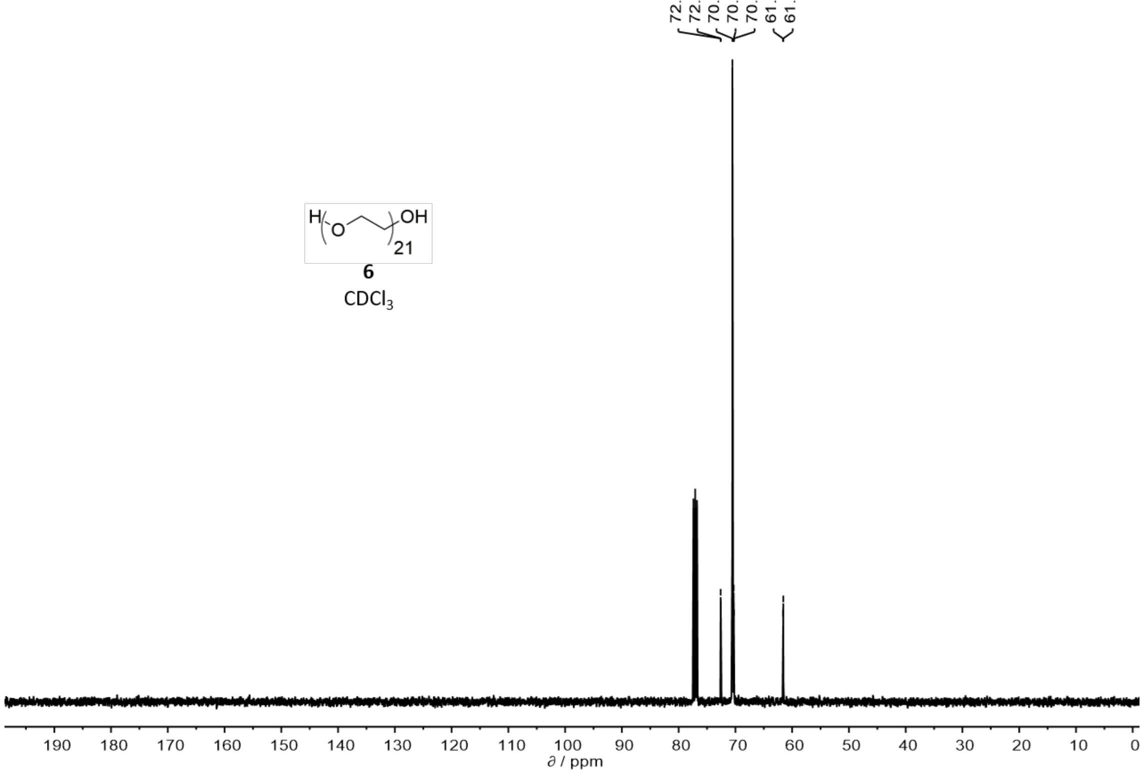
72.60
72.57
70.52
70.31
70.26
61.62
61.57
6
CDCl3
190 180 170 160 150 140 130 120 110 100 90 80 70 60 50 40 30 20 10 0
∂ / ppm

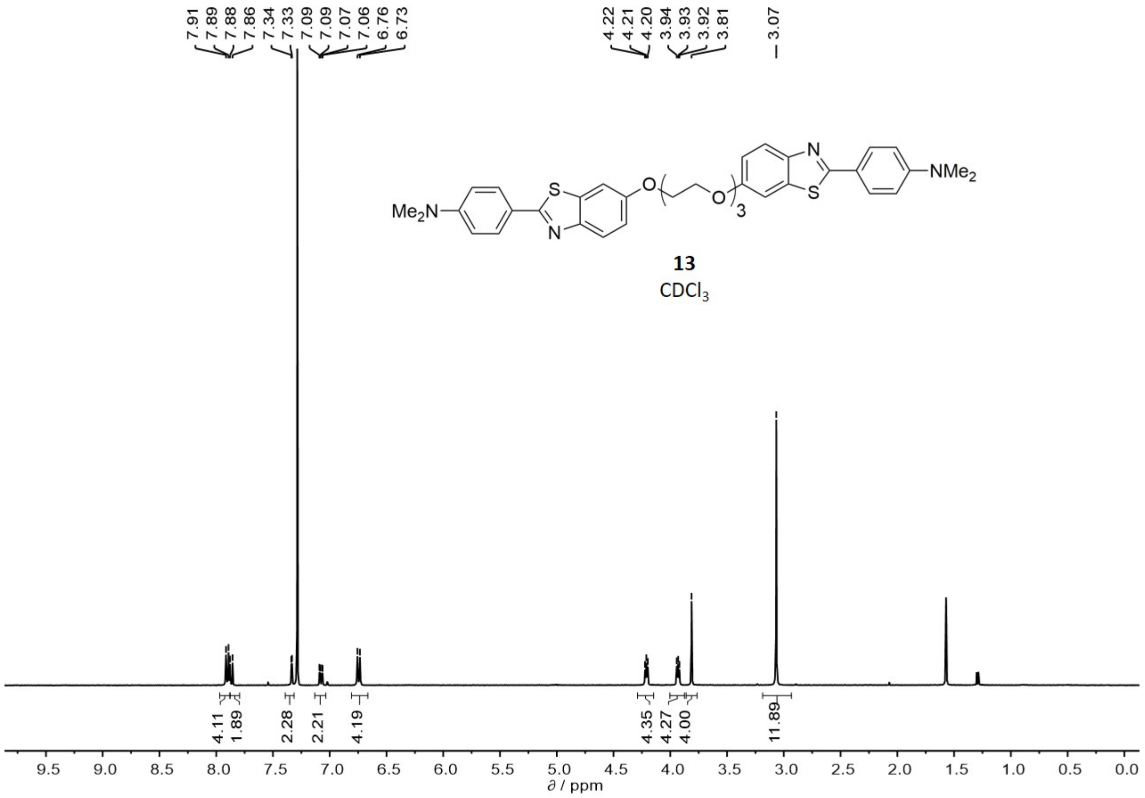
7.91 7.89 7.88 7.86 7.34 7.33 7.09 7.09 7.07 7.06 6.76 6.73
4.22 4.21 4.20 3.94 3.93 3.92 3.81
3.07
Me2N
NMe2
S
N
O
3
13
CDCl3
4.11 1.89 2.28 2.21 4.19
4.35 4.27 4.00
11.89
9.5 9.0 8.5 8.0 7.5 7.0 6.5 6.0 5.5 5.0 4.5 4.0 3.5 3.0 2.5 2.0 1.5 1.0 0.5 0.0
∂ / ppm

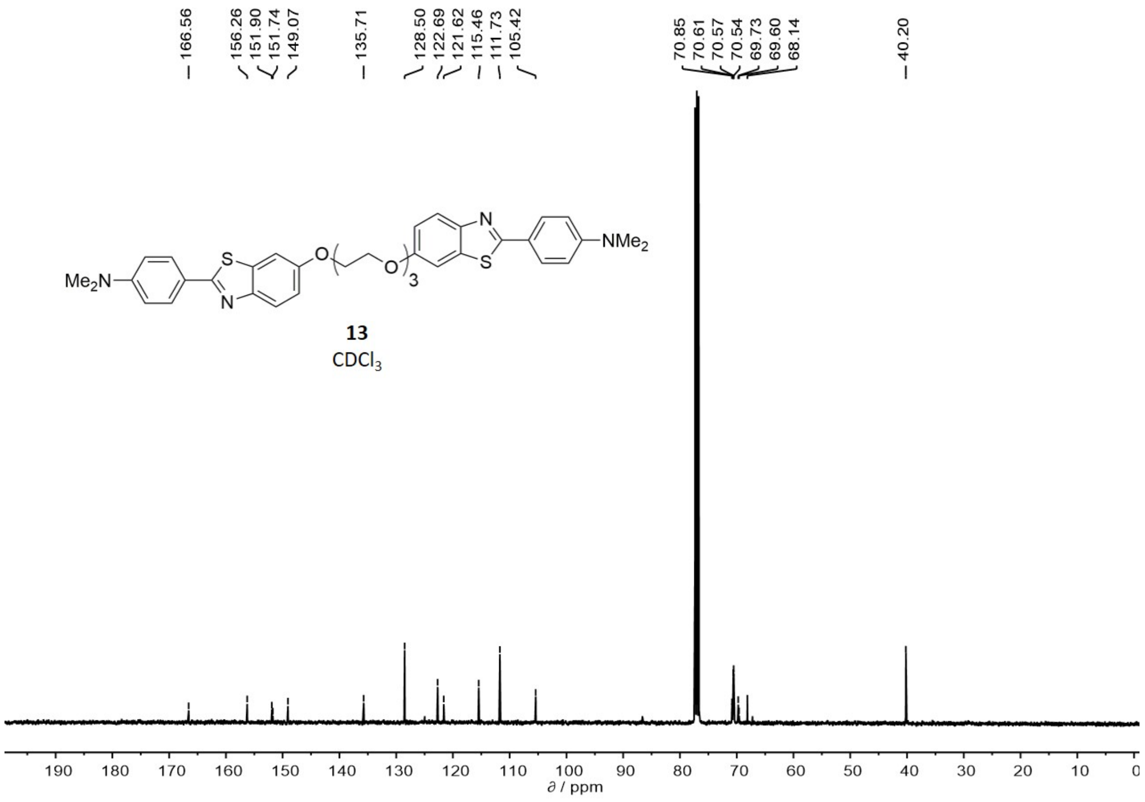
166.56
156.26 151.90 151.74 149.07
135.71
128.50 122.69 121.62 115.46 111.73 105.42
70.85 70.61 70.57 70.54 69.73 69.60 68.14
40.20
Me2N
NMe2
S
N
O
3
13
CDCl3
190 180 170 160 150 140 130 120 110 100 90 80 70 60 50 40 30 20 10 0
∂ / ppm

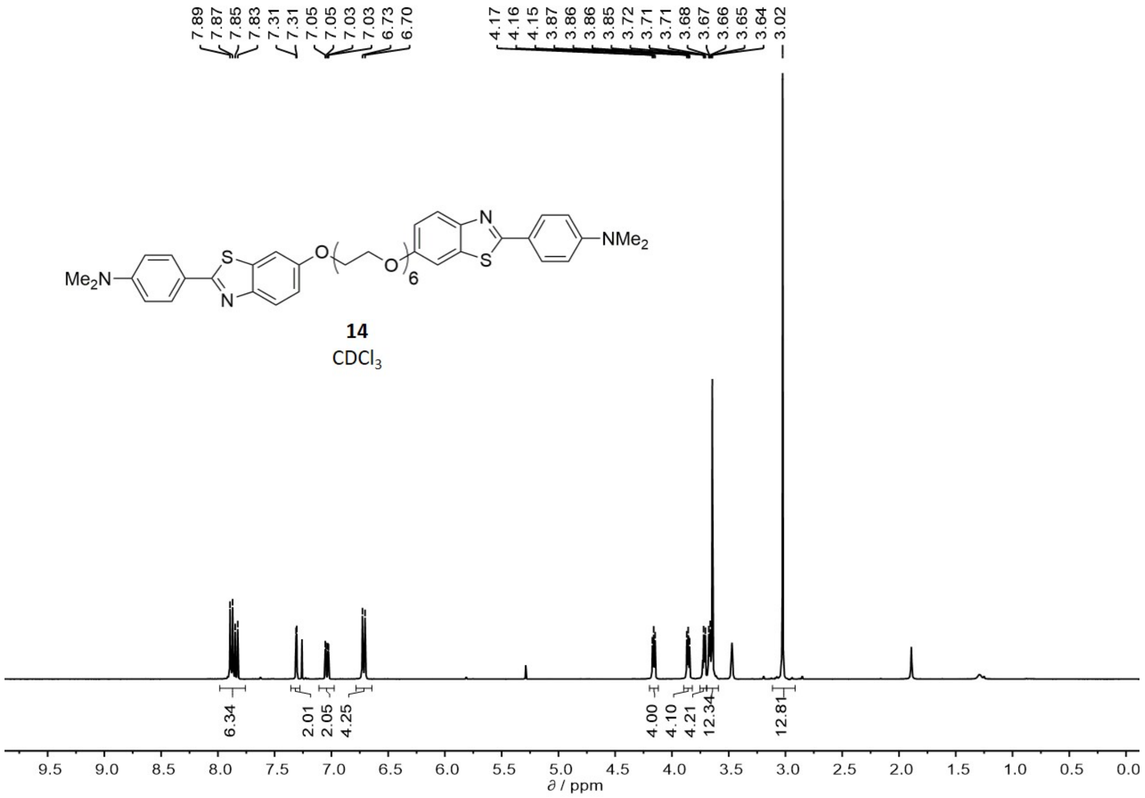
7.89 7.87 7.85 7.83 7.31 7.31 7.05 7.05 7.03 7.03 6.73 6.70
4.17 4.16 4.15 3.87 3.86 3.86 3.85 3.72 3.71 3.71 3.68 3.67 3.66 3.65 3.64 3.02
Me2N
S
N
O
O
6
NMe2
14
CDCl3
6.34
2.01
2.05
4.25
4.00
4.10
4.21
12.34
12.81
∂ / ppm

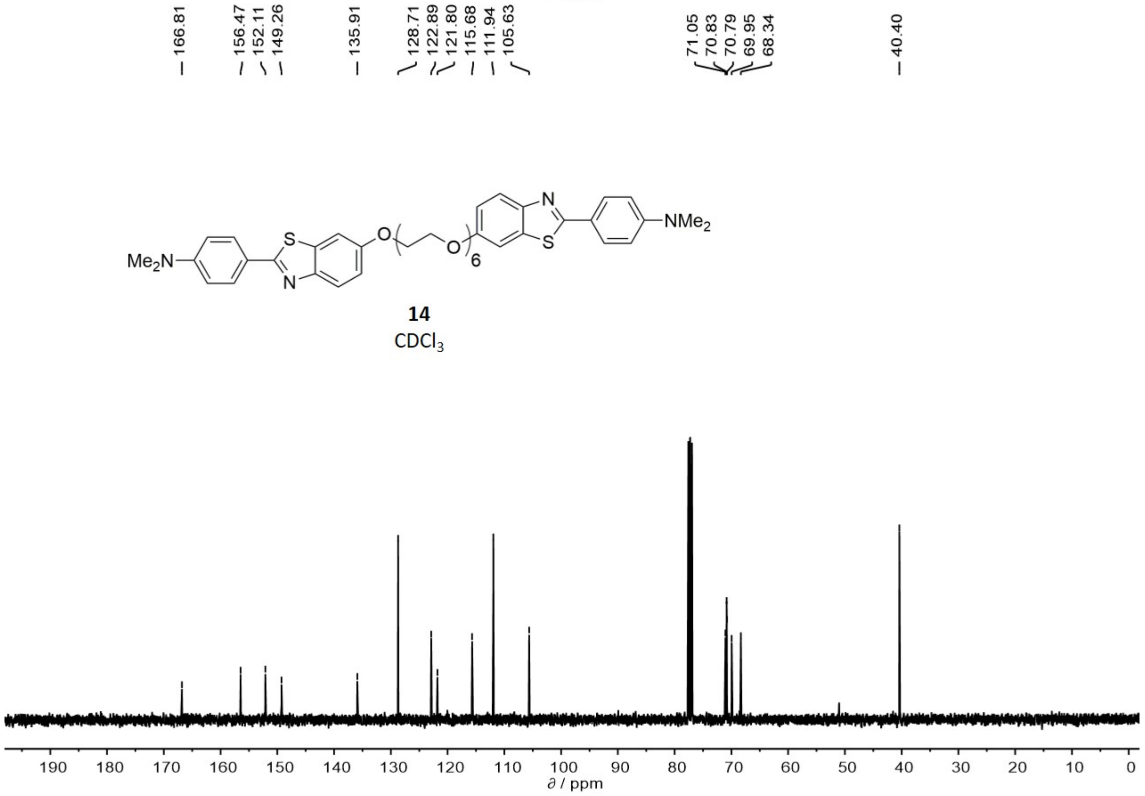
166.81
156.47 152.11 149.26
135.91
128.71 122.89 121.80 115.68 111.94 105.63
71.05 70.83 70.79 69.95 68.34
40.40
Me2N
S
N
O
O
6
NMe2
14
CDCl3
∂ / ppm

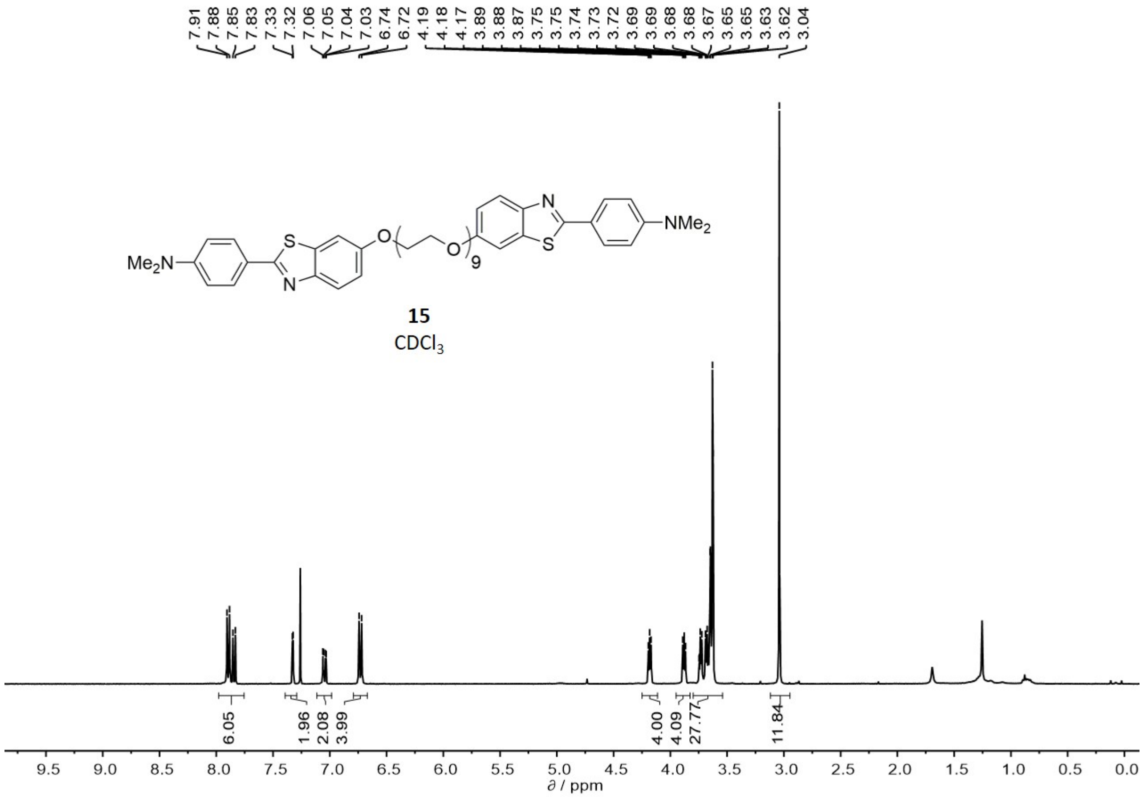
7.91 7.88 7.85 7.83 7.33 7.32 7.06 7.05 7.04 7.03 6.74 6.72 4.19 4.18 4.17 3.89 3.88 3.87 3.75 3.75 3.74 3.73 3.72 3.69 3.69 3.68 3.68 3.67 3.65 3.63 3.62 3.04
Me2N
NMe2
S
N
O
9
15
CDCl3
6.05
1.96
2.08
3.99
4.00
4.09
27.77
11.84
9.5 9.0 8.5 8.0 7.5 7.0 6.5 6.0 5.5 5.0 4.5 4.0 3.5 3.0 2.5 2.0 1.5 1.0 0.5 0.0
∂ / ppm

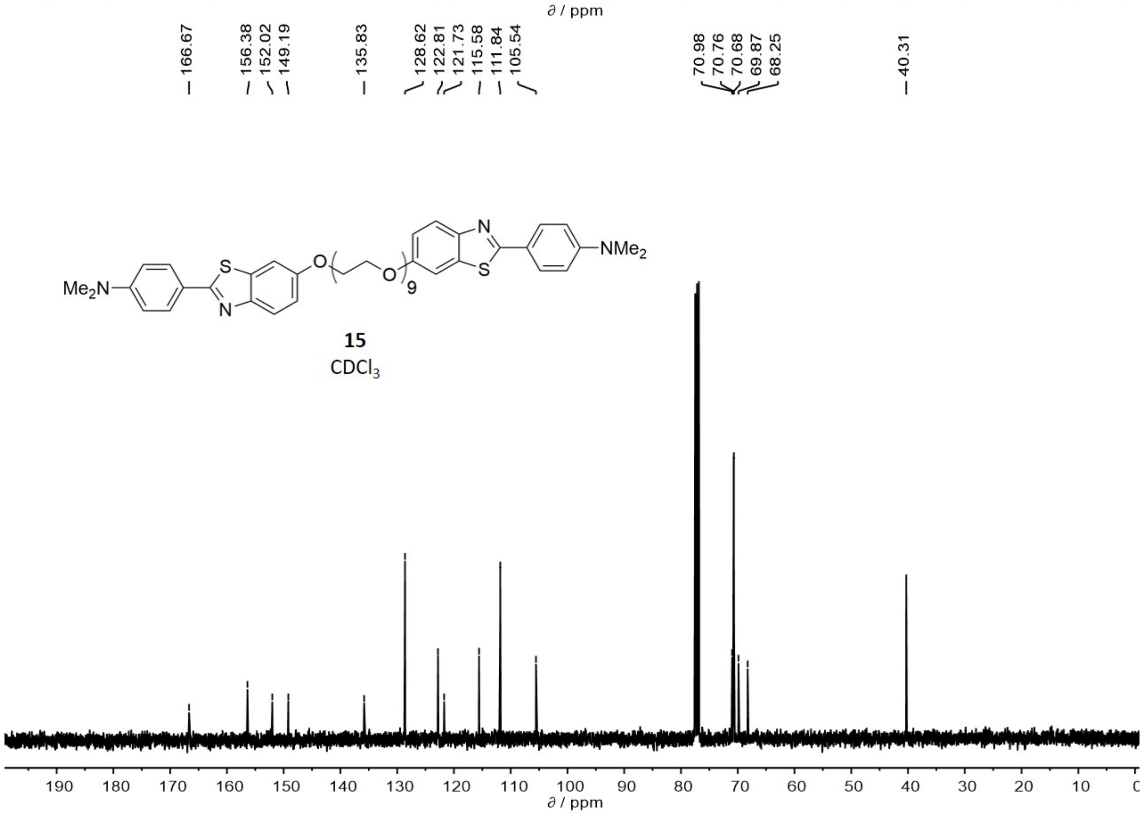
166.67 156.38 152.02 149.19 135.83 128.62 122.81 121.73 115.58 111.84 105.54 70.98 70.76 70.68 69.87 68.25 40.31
Me2N
NMe2
S
N
O
9
15
CDCl3
190 180 170 160 150 140 130 120 110 100 90 80 70 60 50 40 30 20 10 0
∂ / ppm

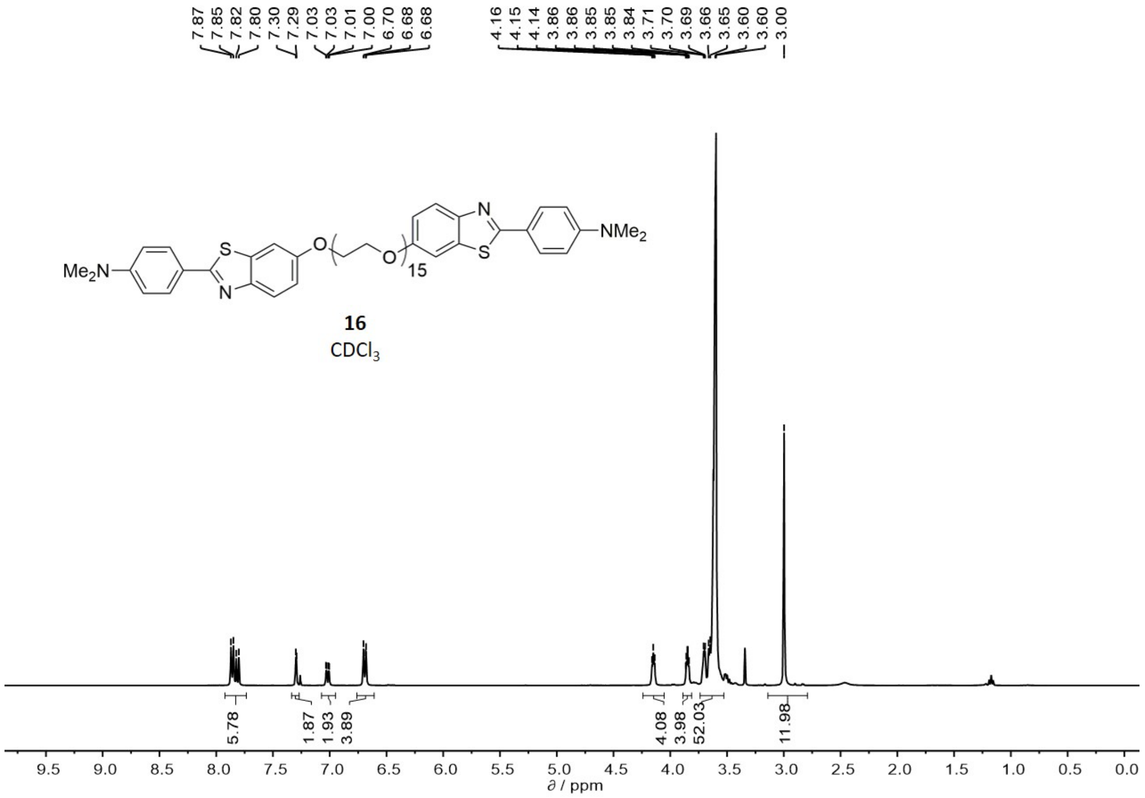

16
$CDCl_3$

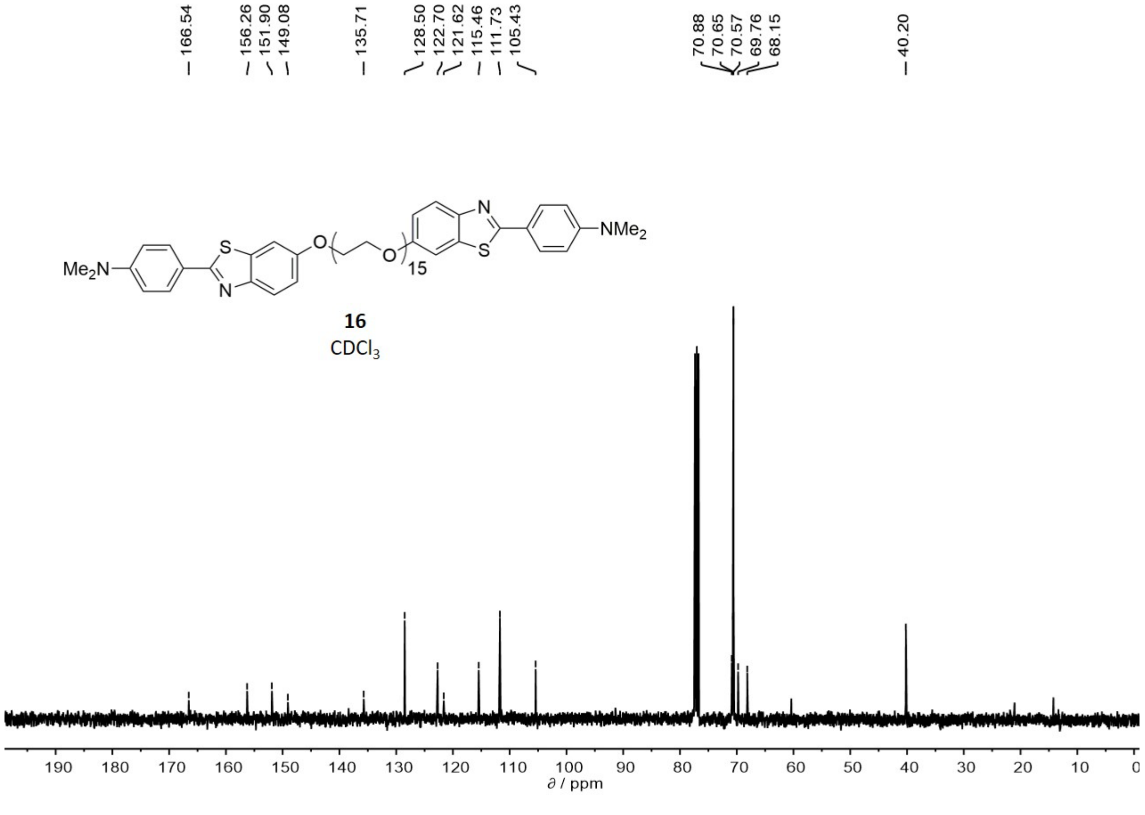

16
$CDCl_3$

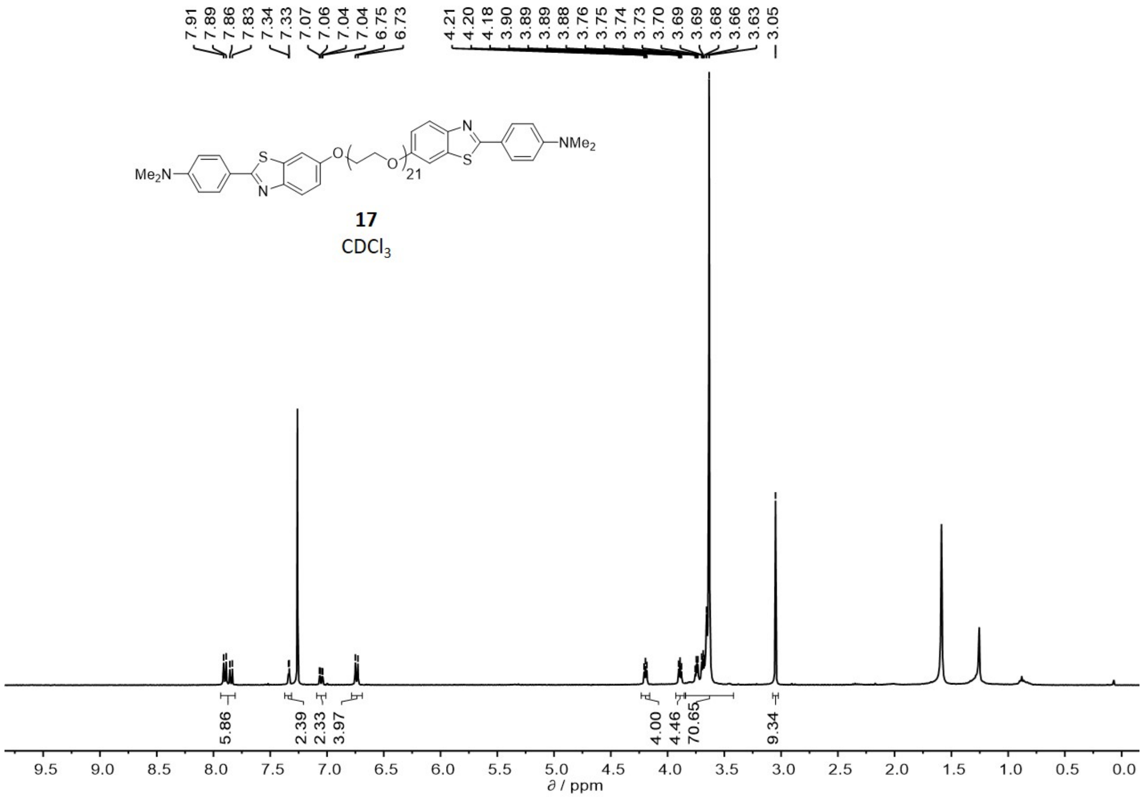
7.91 7.89 7.86 7.83 7.34 7.33 7.07 7.06 7.04 7.04 6.75 6.73
4.21 4.20 4.18 3.90 3.89 3.89 3.88 3.76 3.75 3.74 3.73 3.70 3.69 3.69 3.68 3.66 3.63 3.05
Me2N
NMe2
S
N
O
21
17
CDCl3
5.86
2.39
2.33
3.97
4.00
4.46
70.65
9.34
9.5 9.0 8.5 8.0 7.5 7.0 6.5 6.0 5.5 5.0 4.5 4.0 3.5 3.0 2.5 2.0 1.5 1.0 0.5 0.0
∂ / ppm

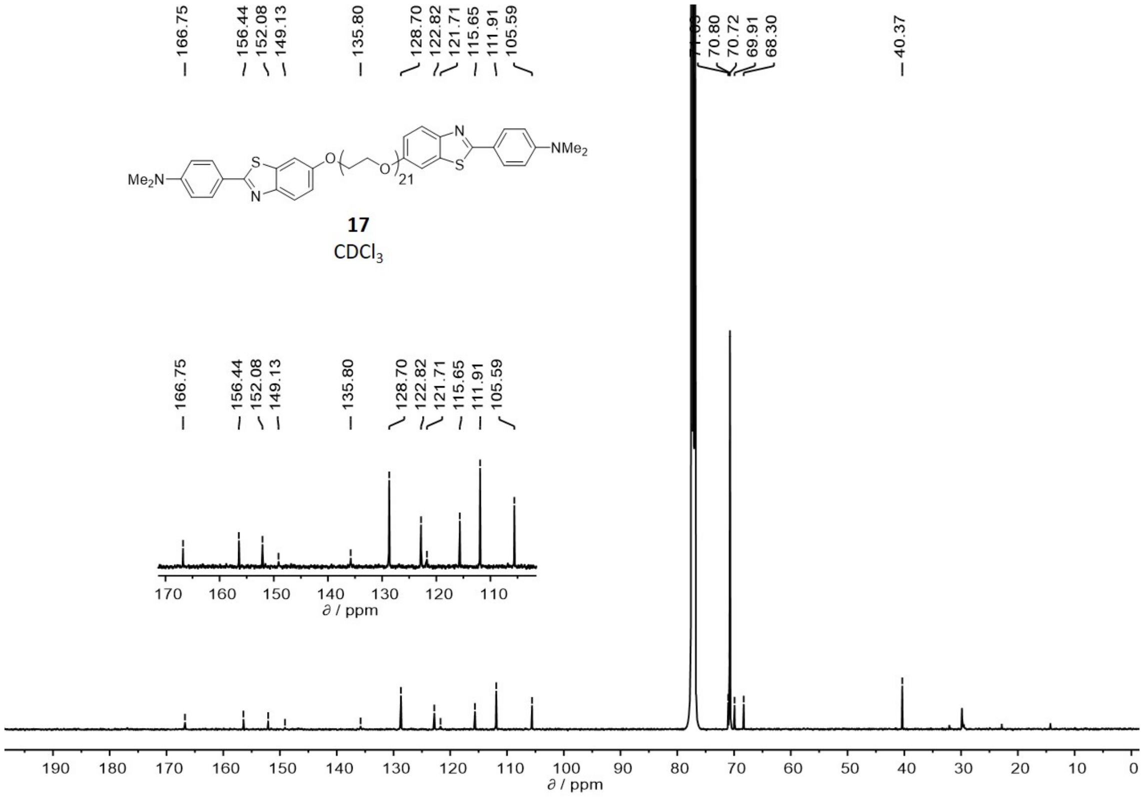
166.75 156.44 152.08 149.13 135.80 128.70 122.82 121.71 115.65 111.91 105.59
70.80 70.72 69.91 68.30
40.37
Me2N
NMe2
S
N
O
21
17
CDCl3
166.75 156.44 152.08 149.13 135.80 128.70 122.82 121.71 115.65 111.91 105.59
170 160 150 140 130 120 110
∂ / ppm
190 180 170 160 150 140 130 120 110 100 90 80 70 60 50 40 30 20 10 0
∂ / ppm

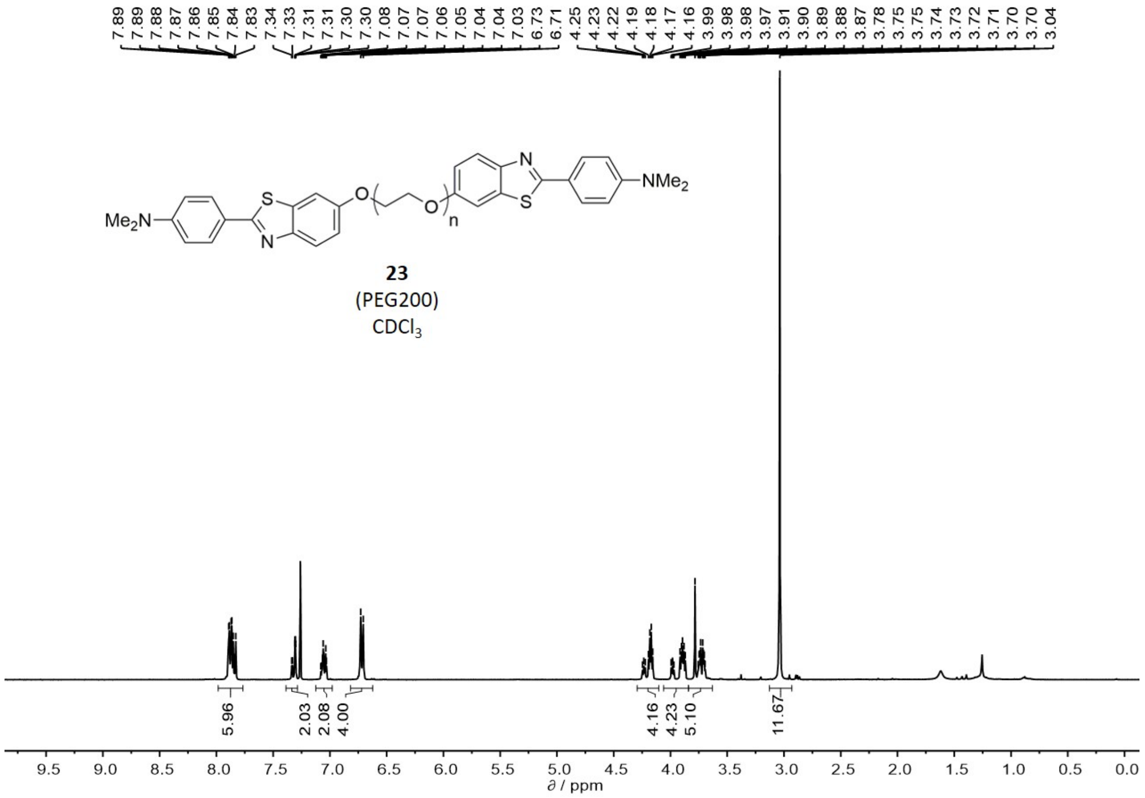
7.89 7.89 7.88 7.87 7.86 7.85 7.84 7.83 7.34 7.33 7.31 7.31 7.30 7.30 7.08 7.07 7.07 7.06 7.05 7.04 7.04 7.03 6.73 6.71 4.25 4.23 4.22 4.19 4.18 4.17 4.16 3.99 3.98 3.98 3.97 3.91 3.90 3.89 3.88 3.87 3.78 3.75 3.75 3.74 3.73 3.72 3.71 3.70 3.70 3.04
Me2N
S
N
O
O
n
NMe2
23
(PEG200)
CDCl3
5.96
2.03
2.08
4.00
4.16
4.23
5.10
11.67
∂ / ppm

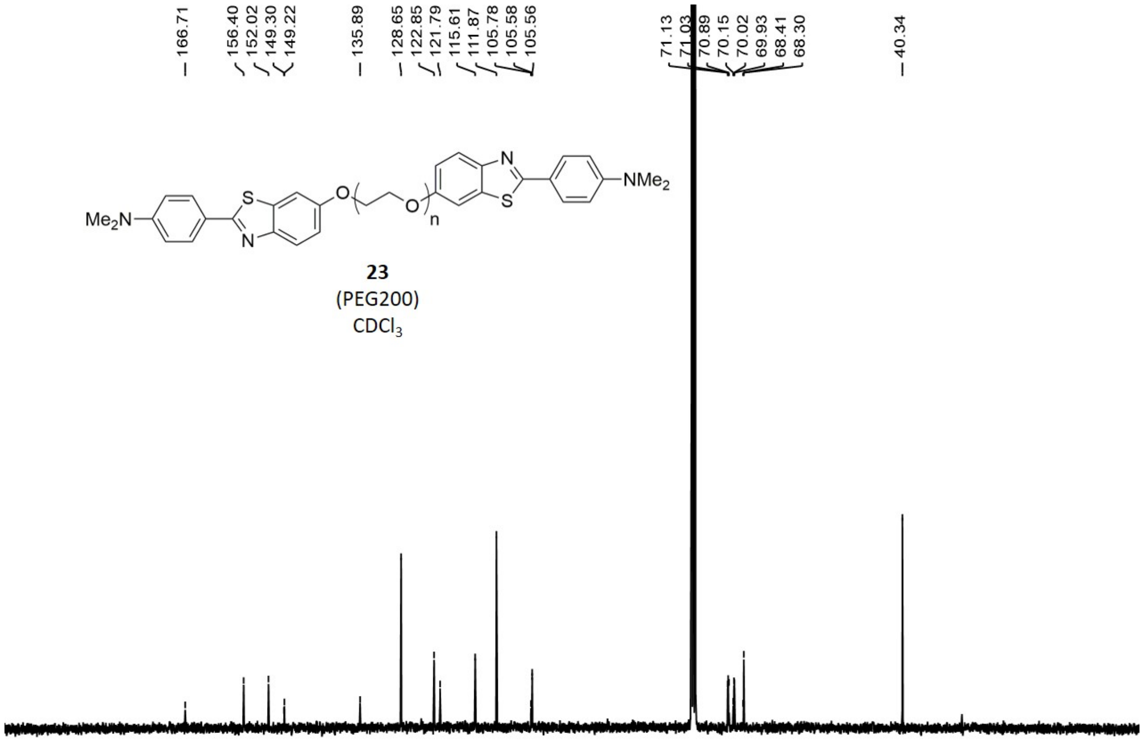
166.71
156.40 152.02 149.30 149.22
135.89
128.65 122.85 121.79 115.61 111.87 105.78 105.58 105.56
71.13 71.03 70.89 70.15 70.02 69.93 68.41 68.30
40.34
Me2N
S
N
O
O
n
NMe2
23
(PEG200)
CDCl3
∂ / ppm

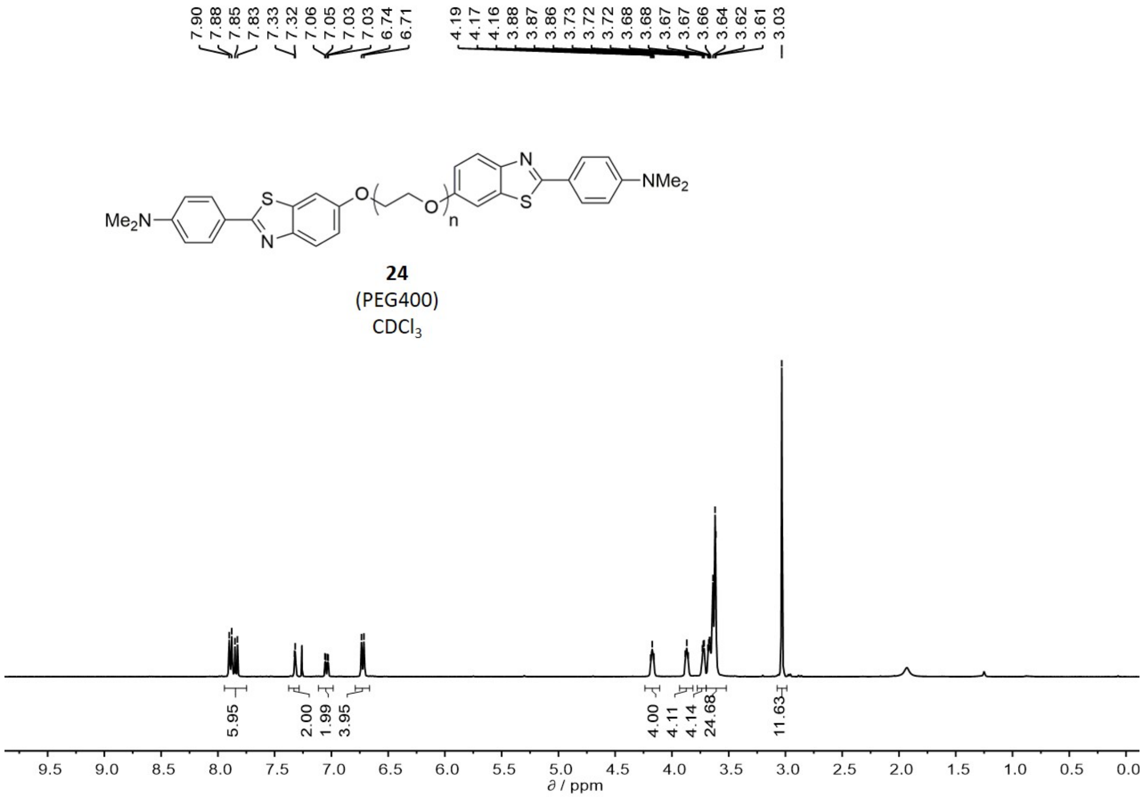
7.90
7.88
7.85
7.83
7.33
7.32
7.06
7.05
7.03
7.03
6.74
6.71
4.19
4.17
4.16
3.88
3.87
3.86
3.73
3.72
3.72
3.68
3.68
3.67
3.67
3.66
3.64
3.62
3.61
3.03
Me2N
S
N
O
O
n
N
S
NMe2
24
(PEG400)
CDCl3
5.95
2.00
1.99
3.95
4.00
4.11
4.14
24.68
11.63
9.5
9.0
8.5
8.0
7.5
7.0
6.5
6.0
5.5
5.0
4.5
4.0
3.5
3.0
2.5
2.0
1.5
1.0
0.5
0.0
∂ / ppm

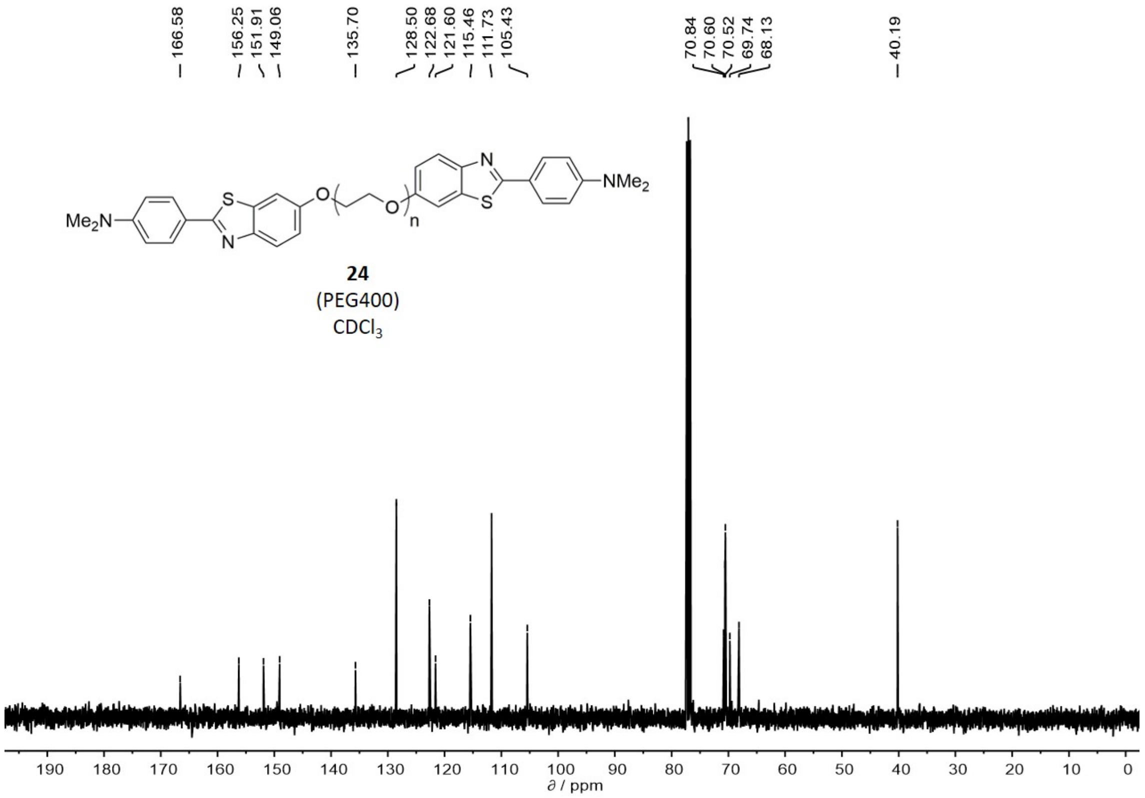
166.58
156.25
151.91
149.06
135.70
128.50
122.68
121.60
115.46
111.73
105.43
70.84
70.60
70.52
69.74
68.13
40.19
Me2N
S
N
O
O
n
N
S
NMe2
24
(PEG400)
CDCl3
190
180
170
160
150
140
130
120
110
100
90
80
70
60
50
40
30
20
10
0
∂ / ppm

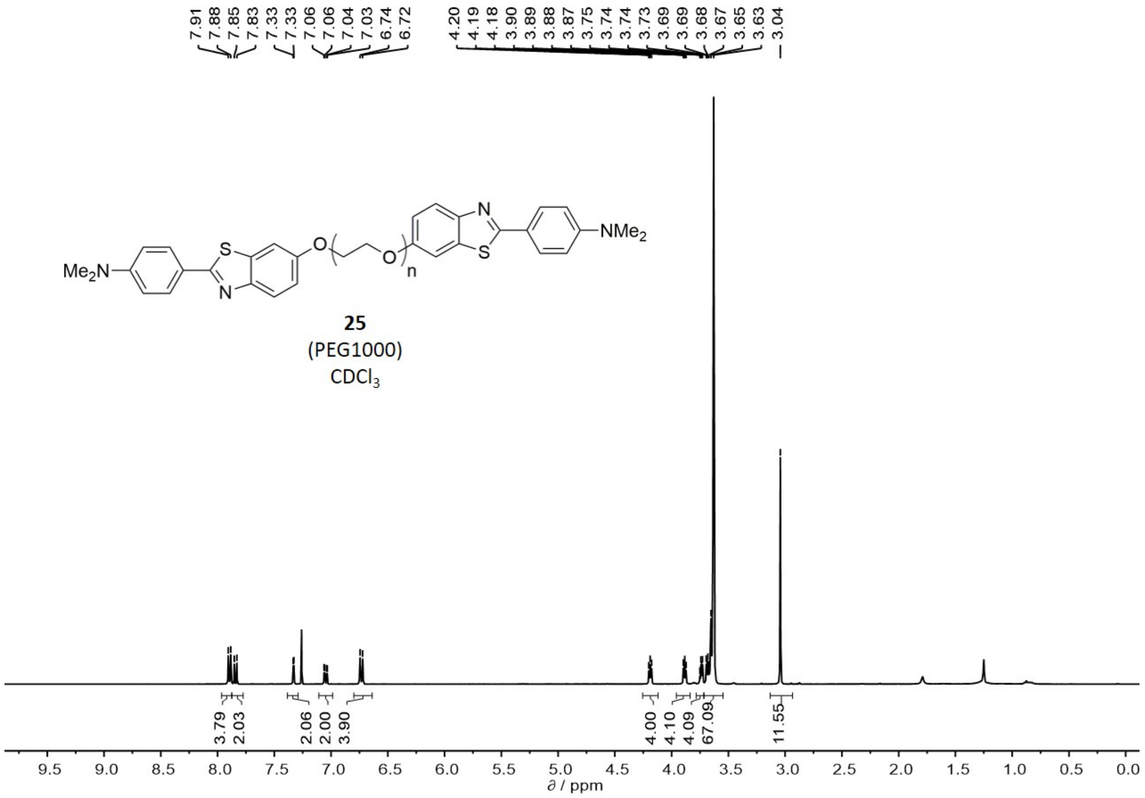
7.91
7.88
7.85
7.83
7.33
7.33
7.06
7.06
7.04
7.03
6.74
6.72
4.20
4.19
4.18
3.90
3.89
3.88
3.87
3.75
3.74
3.74
3.73
3.69
3.69
3.68
3.67
3.65
3.63
3.04
Me2N
S
N
O
O
n
N
S
NMe2
25
(PEG1000)
CDCl3
3.79
2.03
2.06
2.00
3.90
4.00
4.10
4.09
67.09
11.55
∂ / ppm

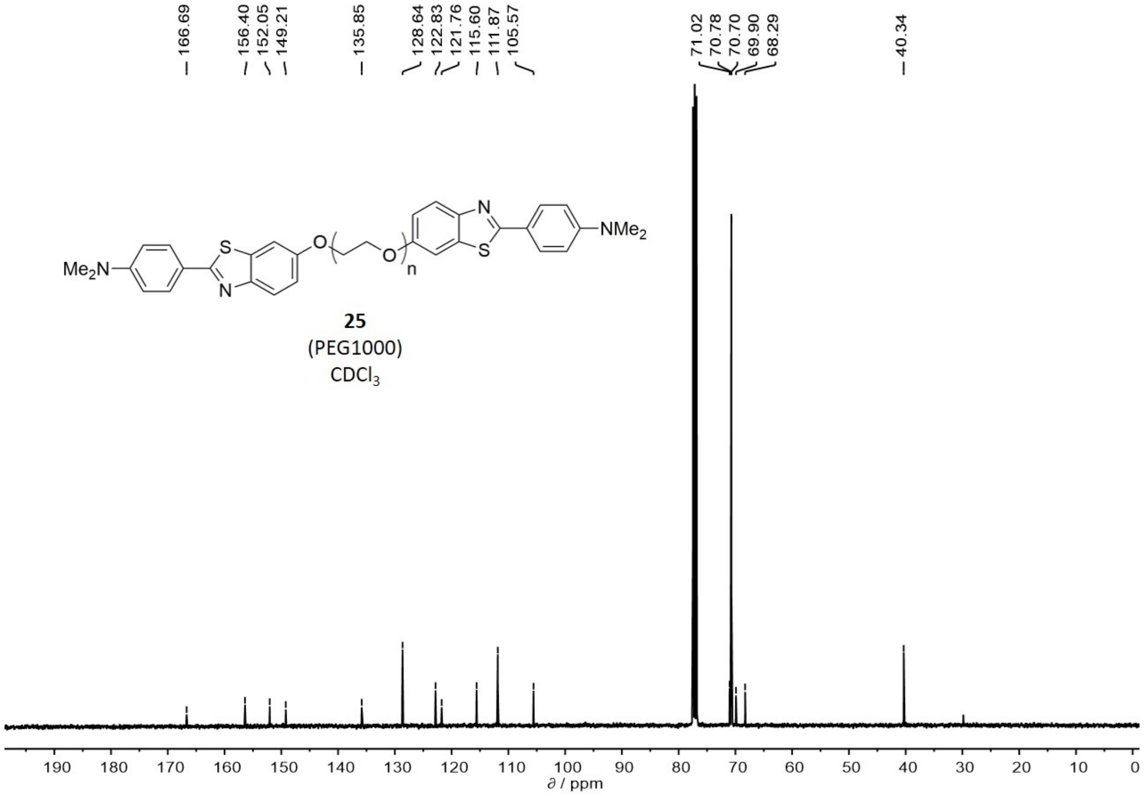
166.69
156.40
152.05
149.21
135.85
128.64
122.83
121.76
115.60
111.87
105.57
71.02
70.78
70.70
69.90
68.29
40.34
Me2N
S
N
O
O
n
N
S
NMe2
25
(PEG1000)
CDCl3
∂ / ppm

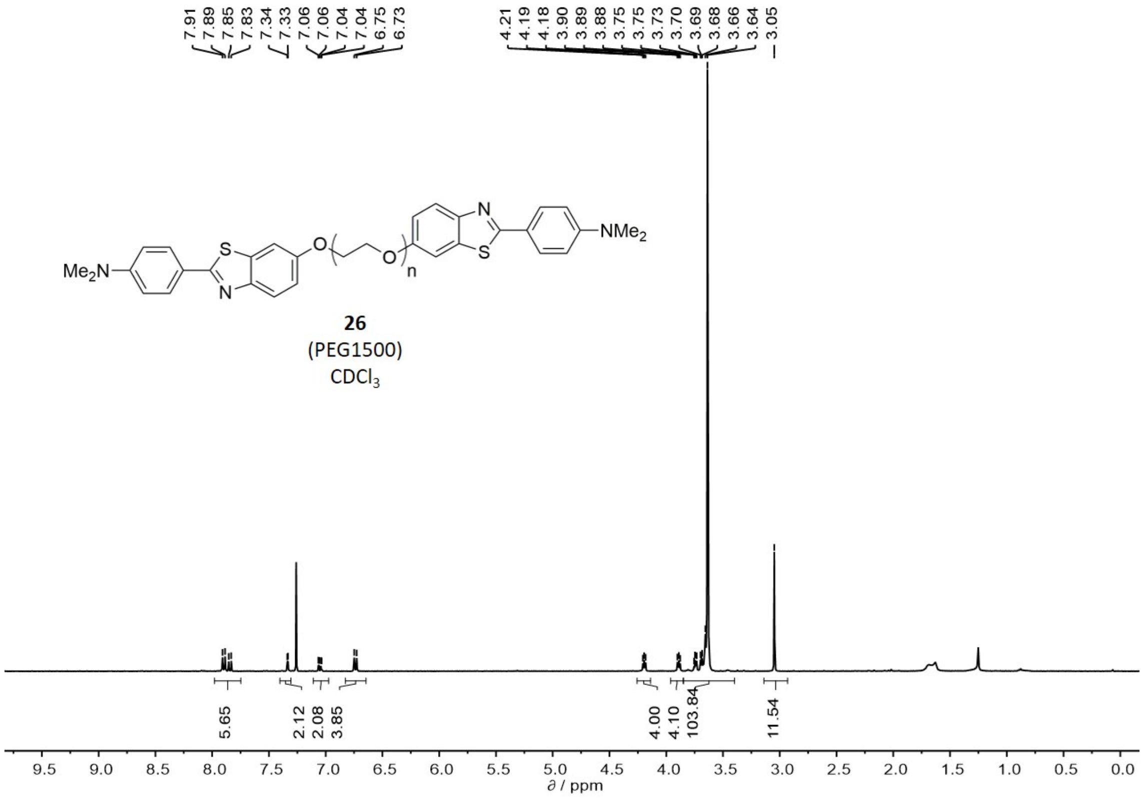
7.91 7.89 7.85 7.83 7.34 7.33 7.06 7.06 7.04 7.04 6.75 6.73
4.21 4.19 4.18 3.90 3.89 3.88 3.75 3.75 3.73 3.70 3.69 3.68 3.66 3.64 3.05
Me2N
NMe2
26
(PEG1500)
CDCl3
5.65
2.12 2.08 3.85
4.00 4.10 103.84
11.54
9.5 9.0 8.5 8.0 7.5 7.0 6.5 6.0 5.5 5.0 4.5 4.0 3.5 3.0 2.5 2.0 1.5 1.0 0.5 0.0
∂ / ppm

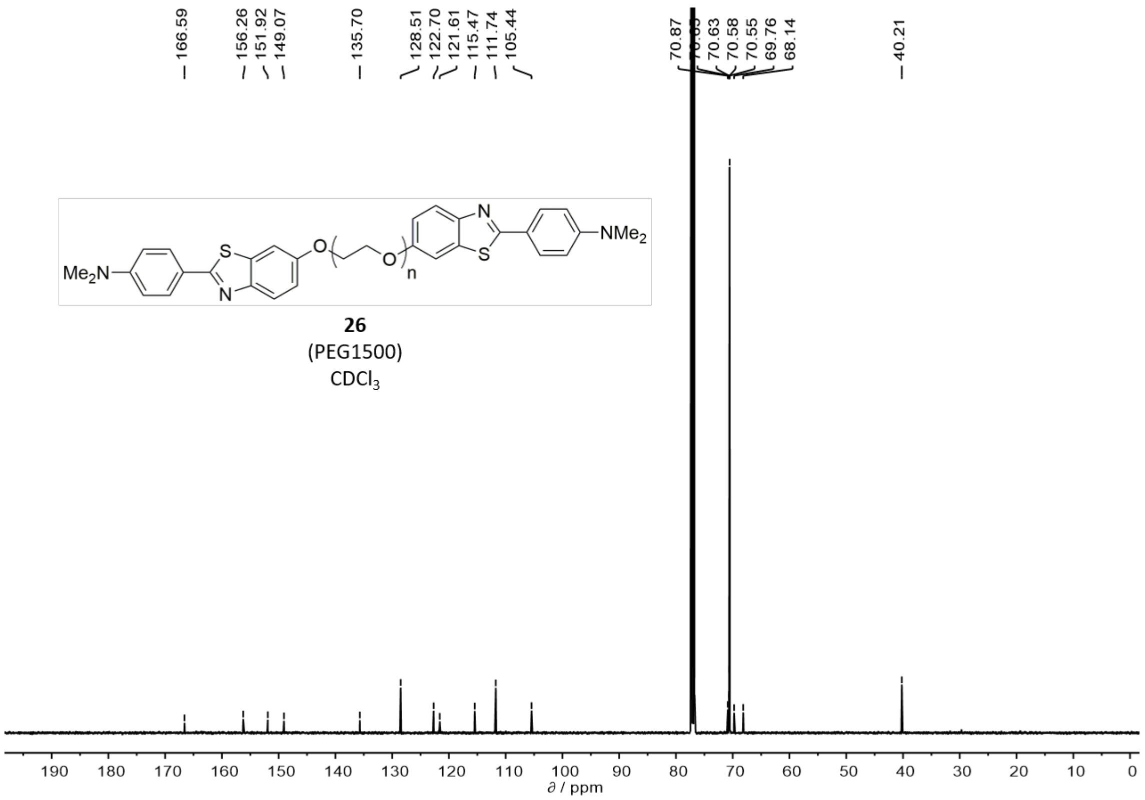
166.59
156.26 151.92 149.07
135.70
128.51 122.70 121.61 115.47 111.74 105.44
70.87 70.63 70.58 70.55 69.76 68.14
40.21
Me2N
NMe2
26
(PEG1500)
CDCl3
190 180 170 160 150 140 130 120 110 100 90 80 70 60 50 40 30 20 10 0
∂ / ppm

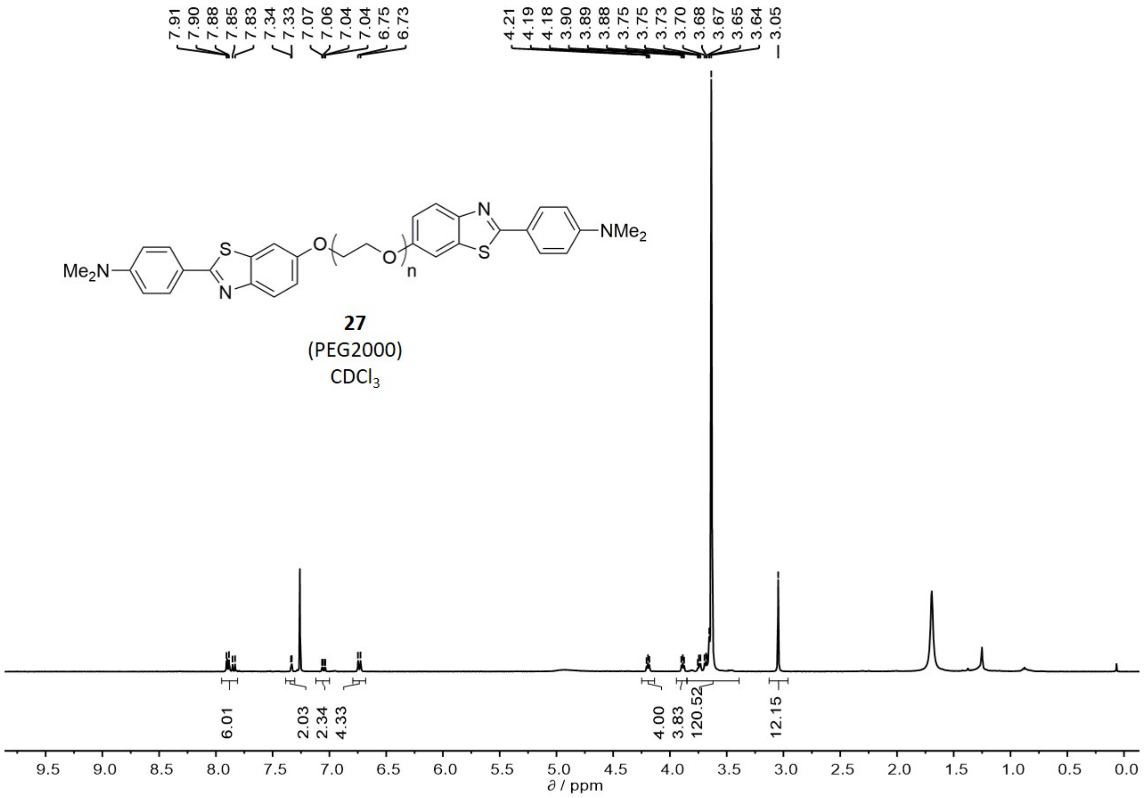
7.91
7.90
7.88
7.85
7.83
7.34
7.33
7.07
7.06
7.04
7.04
6.75
6.73
4.21
4.19
4.18
3.90
3.89
3.88
3.75
3.75
3.73
3.70
3.68
3.67
3.65
3.64
3.05
Me2N
S
N
O
O
n
N
S
NMe2
27
(PEG2000)
CDCl3
6.01
2.03
2.34
4.33
4.00
3.83
120.52
12.15
9.5 9.0 8.5 8.0 7.5 7.0 6.5 6.0 5.5 5.0 4.5 4.0 3.5 3.0 2.5 2.0 1.5 1.0 0.5 0.0
∂ / ppm

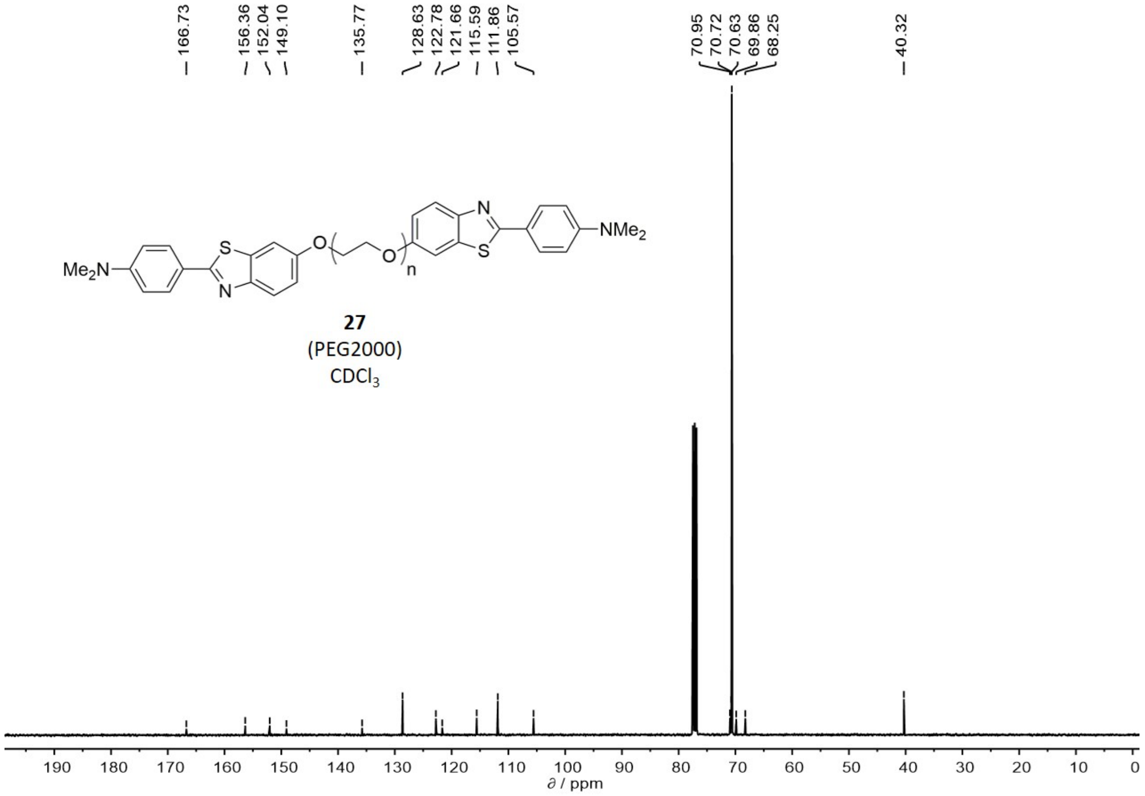
166.73
156.36
152.04
149.10
135.77
128.63
122.78
121.66
115.59
111.86
105.57
70.95
70.72
70.63
69.86
68.25
40.32
Me2N
S
N
O
O
n
N
S
NMe2
27
(PEG2000)
CDCl3
190 180 170 160 150 140 130 120 110 100 90 80 70 60 50 40 30 20 10 0
∂ / ppm

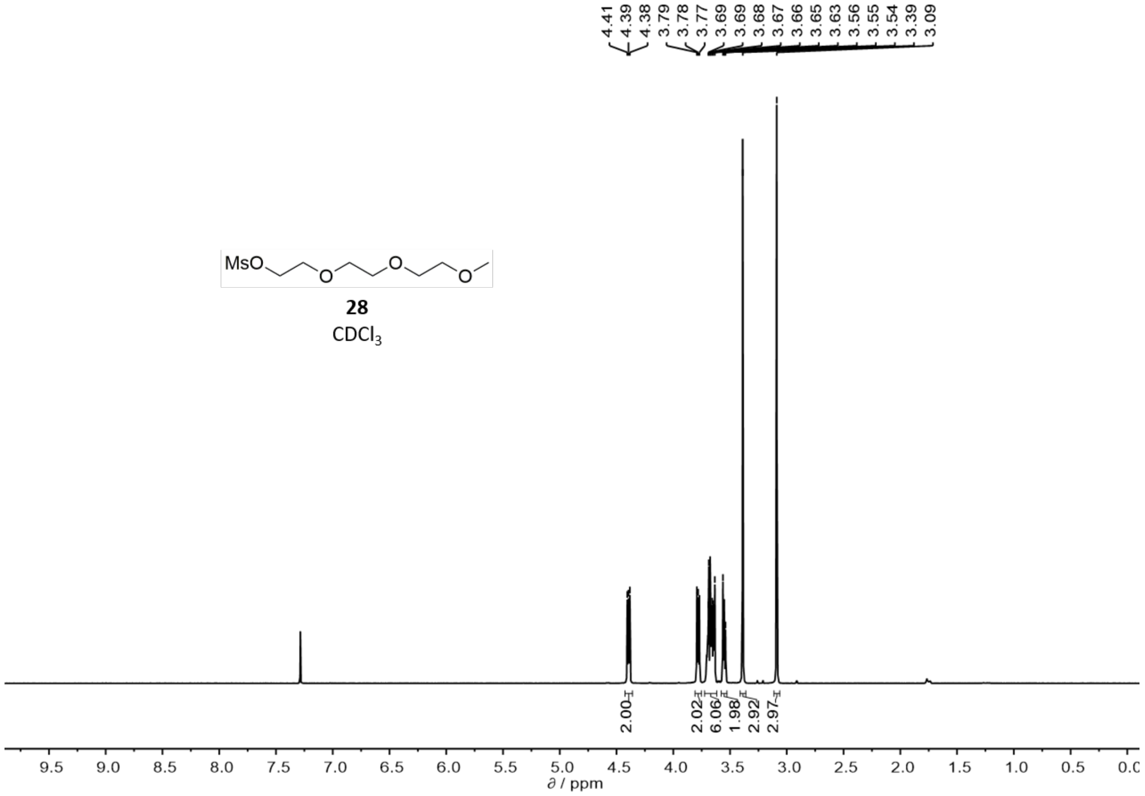

28
$CDCl_3$

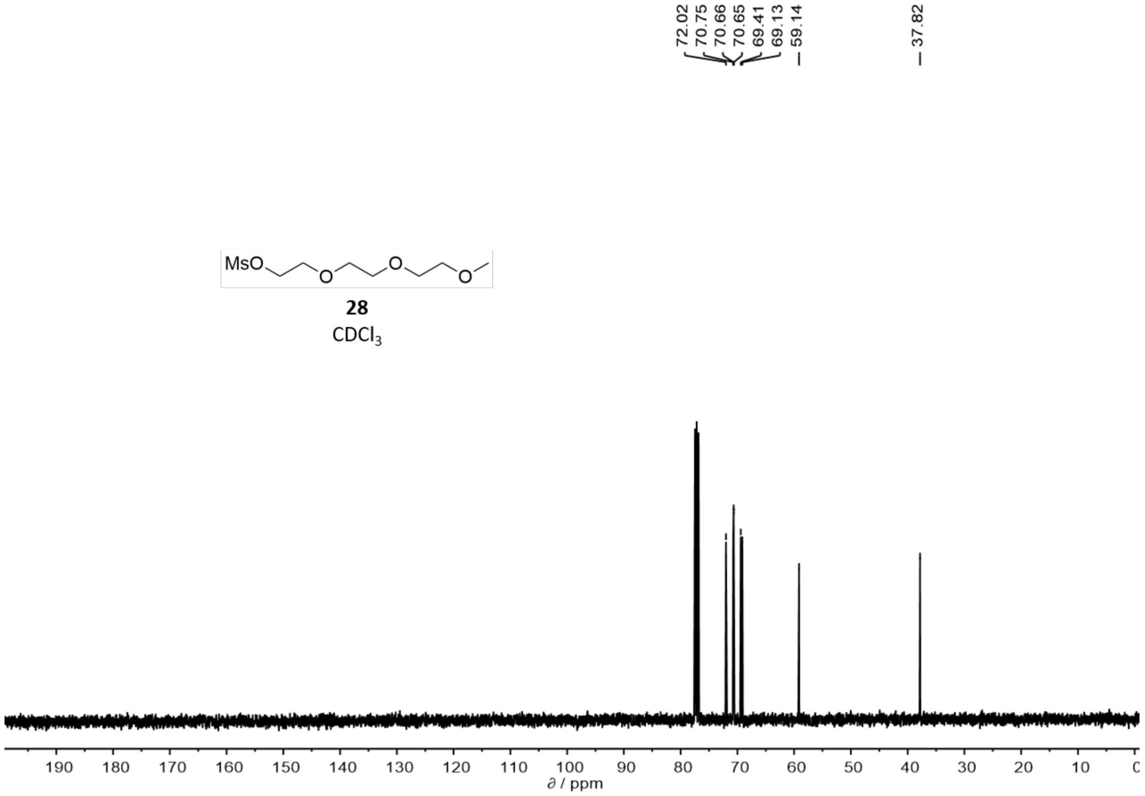

28
$CDCl_3$

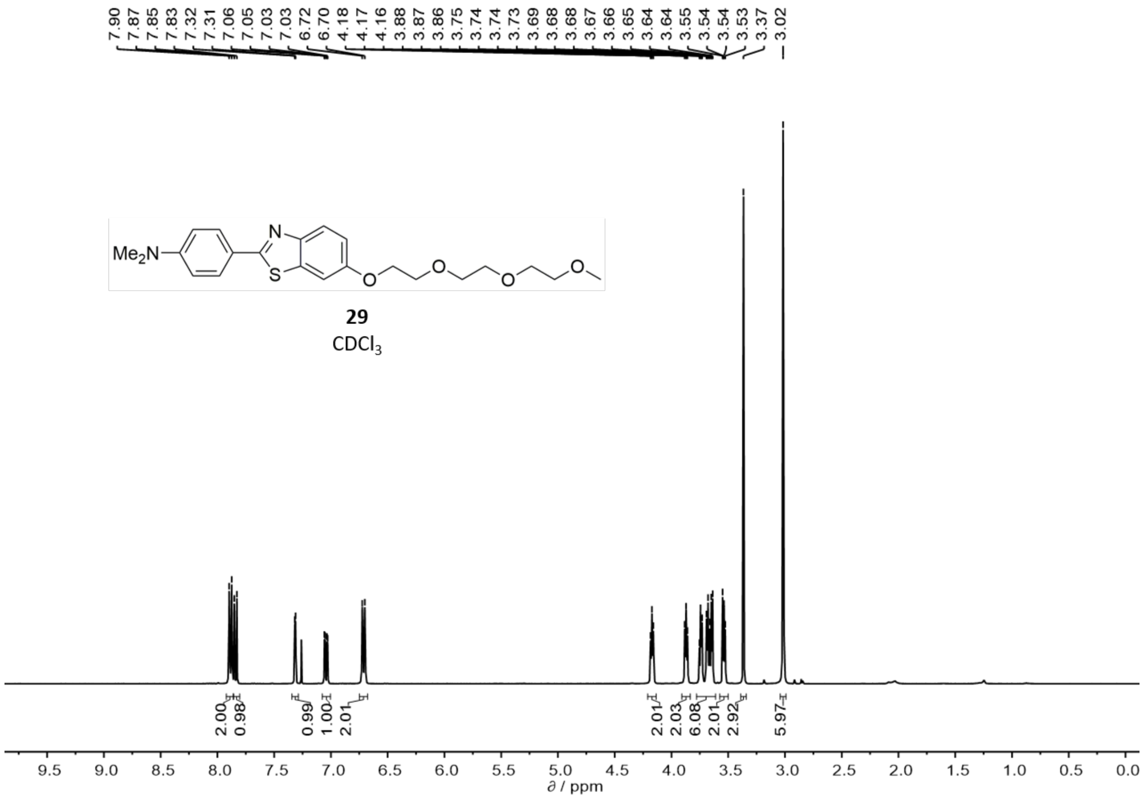

29
$CDCl_3$

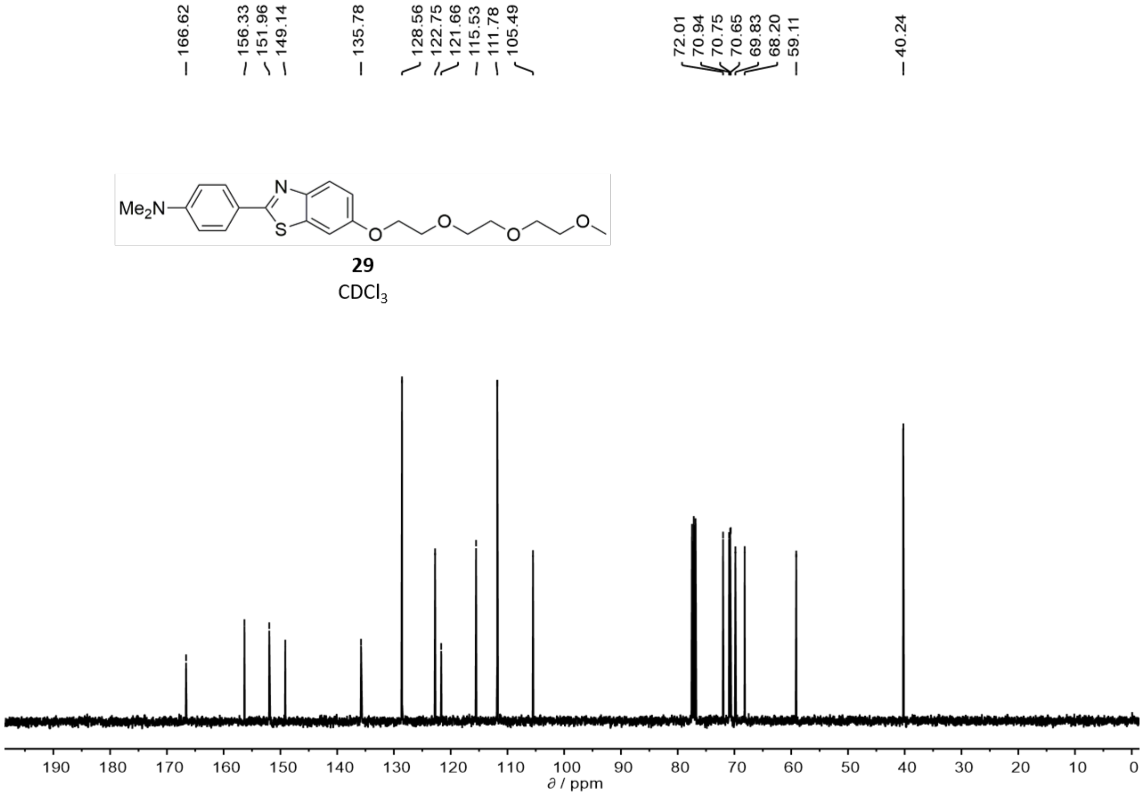

29
$CDCl_3$

3. LCMS analysis

All the experiments were carried out on an LCMS Agilent HP1200 system equipped with 321 pump ion trap and, diode array detector (DAD) modules. The column used was a HICHROM 2.5 µm RPB (4.6 × 50 mm). The experimental conditions are listed as follows: flow rate was 1 mL/min, the injection volume was typically 5 µL, and the absorbance was monitored at 354 nm. Mobile phase A contains milliQ water with a 0.1 % of formic acid and, mobile phase B contains MeCN with a 0.1 % of formic acid.
The gradient used for the runs was: increases from 20% to 100% of phase B in 2 min, and hold at 100% for 3 minutes.

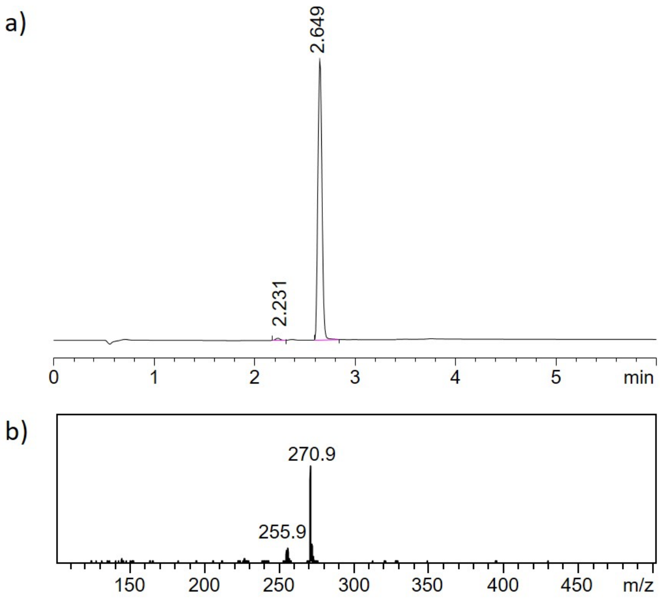

Figure S1. HPLC trace (a) and mass spectrum (b) of **1**.

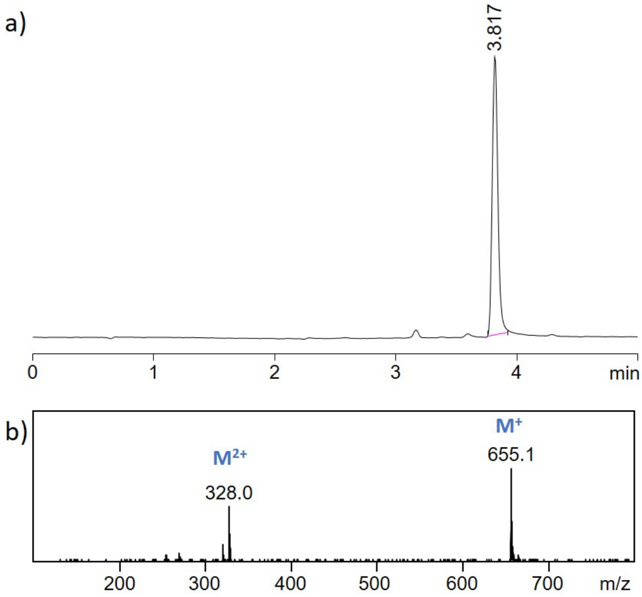

Figure S2. HPLC trace (a) and mass spectrum (b) of compound **13**.

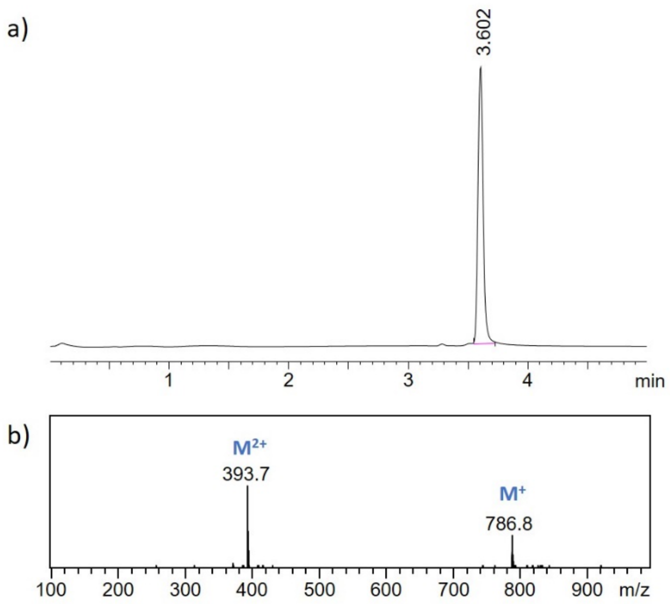

Figure S3. HPLC trace (a) and mass spectrum (b) of compound **14**.

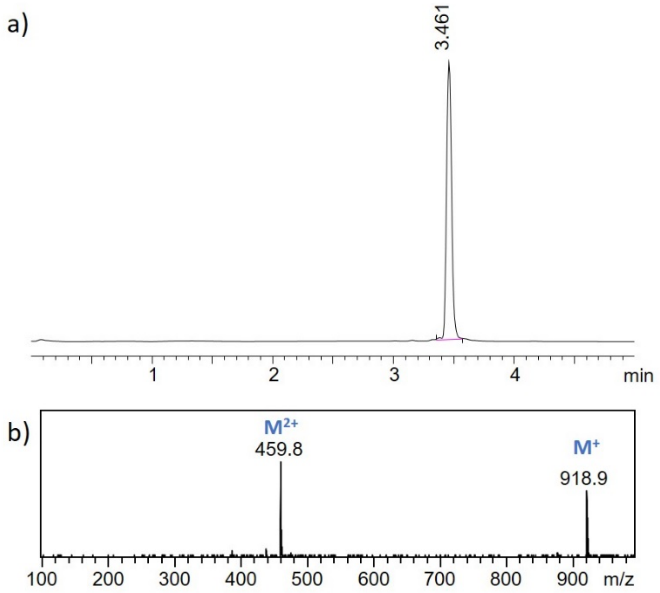

Figure S4. HPLC trace (a) and mass spectrum (b) of compound **15**.

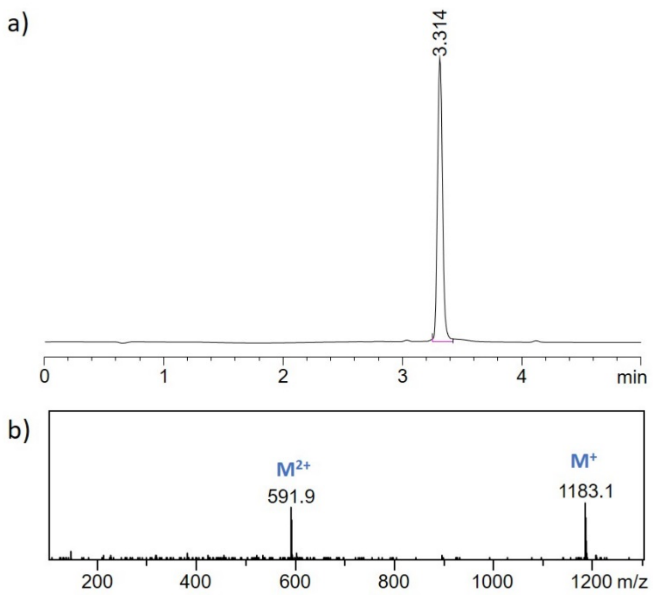

Figure S5. HPLC trace (a) and mass spectrum (b) of compound **16**.

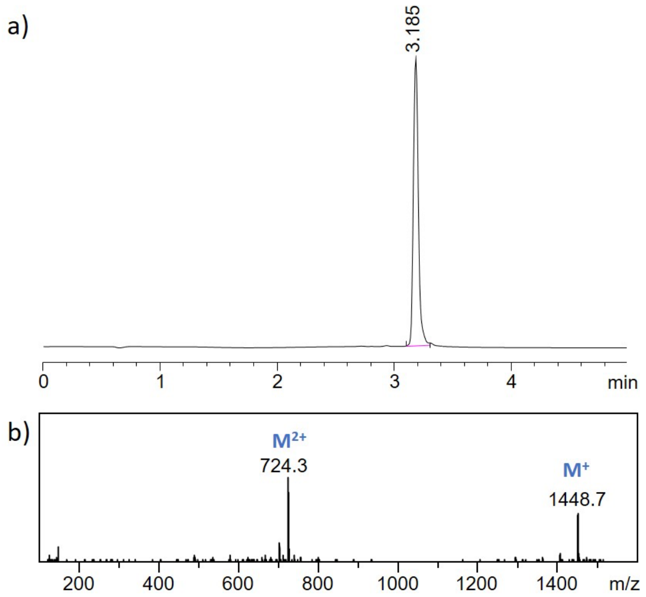

Figure S6. HPLC trace (a) and mass spectrum (b) of compound **17**.

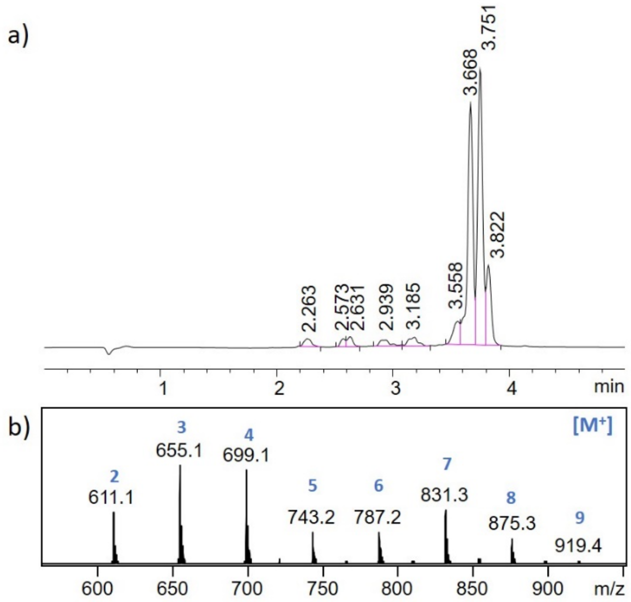

Figure S7. HPLC trace (a) and mass spectrum (b) of compound **23**. Individual peaks are labelled with the number of PEG units in the chain linking the two BTA head groups.

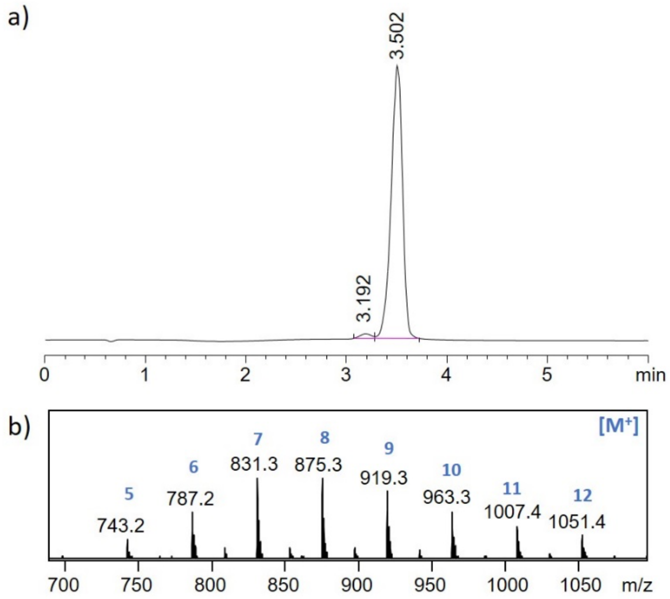

Figure S8. HPLC trace (a) and mass spectrum (b) of compound **24**. Individual peaks are labelled with the number of PEG units in the chain linking the two BTA head groups.

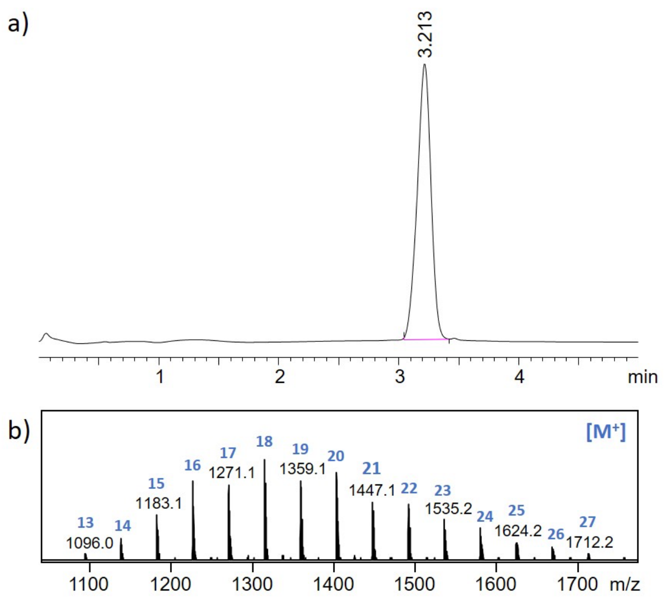

Figure S9. HPLC trace (a) and mass spectrum (b) of compound **25**. Individual peaks are labelled with the number of PEG units in the chain linking the two BTA head groups.

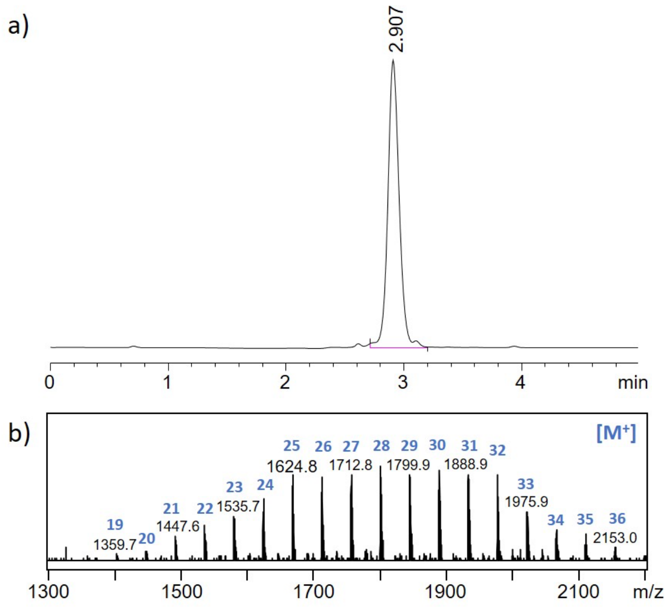

Figure S10. HPLC trace (a) and mass spectrum (b) of compound **26**. Individual peaks are labelled with the number of PEG units in the chain linking the two BTA head groups.

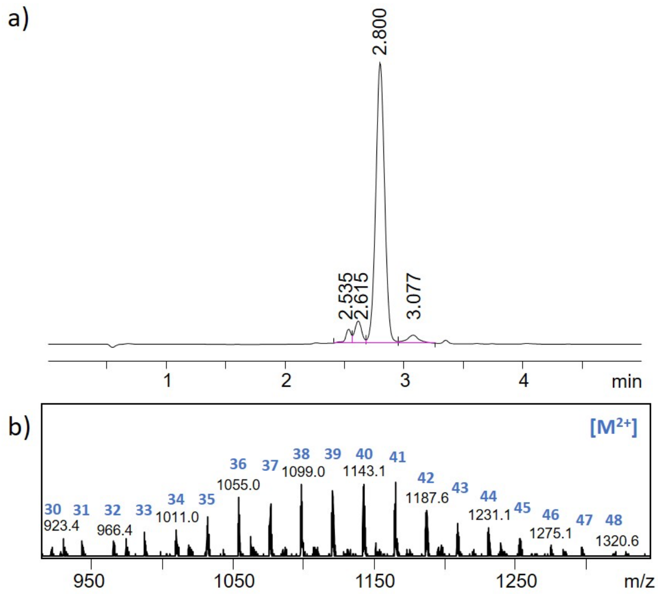

Figure S11. HPLC trace (a) and mass spectrum (b) of compound **27**. Individual peaks are labelled with the number of PEG units in the chain linking the two BTA head groups.

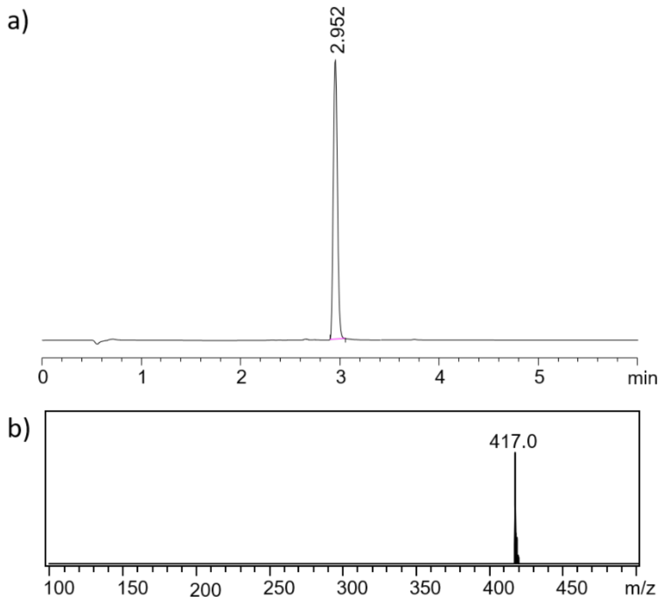

Figure S12. HPLC trace (a) and mass spectrum (b) of compound **29**.

Table S1. HPLC retention times observed for monodisperse BTA dimers.

Compound	Ret. Time (min)	M_w (g/mol)
**14**	3.57	787.0
**15**	3.46	919.1
**16**	3.31	1183.5
**17**	3.17	1447.7

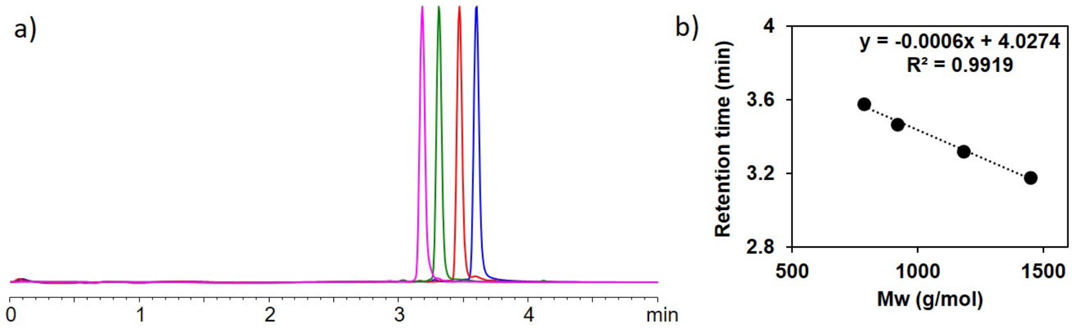

Figure S13. a) Superimposed HPLC traces: **14** (blue), **15** (red), **16** (green) and **17** (pink). b) Representation of the retention time vs the molecular weight observed for the mondisperse BTA dimers.

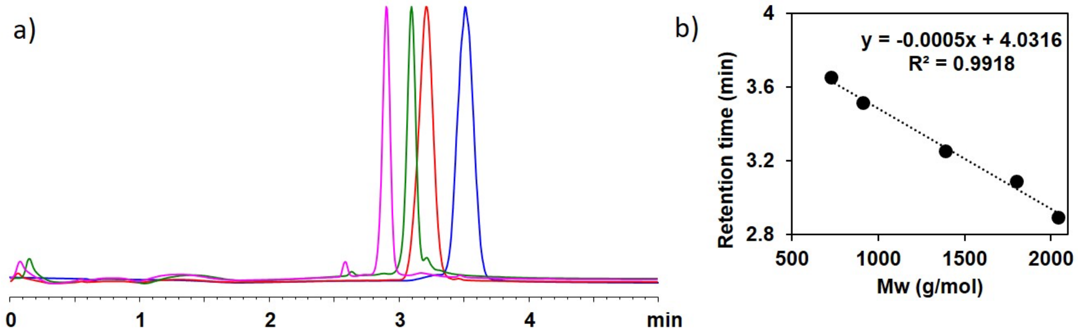

Figure S14. Superimposed HPLC traces: **24** (blue), **25** (red), **26** (green) and **27** (pink). b) Representation of the retention time vs the molecular weight observed for the polydisperse BTA dimers.

The following equations were used to calculate the number average weight (M_N), the weight-average molecular weight and the polydispersity index (PD) of **27**-**31**. M_i are the molecular masses of the peaks observed in the mass spectrum, and N_i are the intensities of the peaks.

$$M_N = \frac{\sum M_i N_i}{\sum N_i}$$

$$M_W = \frac{\sum (M_i)^2 N_i}{\sum M_i N_i}$$

$$PD = \frac{M_W}{M_N}$$

Table S2. Calculated molecular weight distributions for polydisperse BTA dimers.

**Compound**	**Ret. Time (min)**	**M_w (g/mol) Expected**	**M_w (g/mol) Calculated Ret.time**	**M_w (g/mol)**	**M_N (g/mol)**	**PDI**
**23**	3.65	710	625.6	725.7	736.5	1.014
**24**	3.51	910	853.04	909.7	924.0	1.015
**25**	3.25	1510	1287.3	1389.3	1406.8	1.012
**26**	3.08	2010	1563.7	1799.3	1815.6	1.009
**27**	2.89	2510	1887.9	2039.3	2234.5	1.095

4. Fluorescence characterization of the dimers

1 mL of stock solutions of the corresponding dyes (1 mM) were prepared in pure DMSO. Experiments were carried out at 25°C. The samples were prepared by dilution of the corresponding amount of stock solution in PBS 1X. All the spectra were recorded using a 2 µM solution of the dye. The concentration used was 2 µM. The excitation spectra were recorded using 425 nm as emission wavelength and reading between 250 and 405 nm (blue). The emission spectra were recorded using 355 nm as excitation wavelength and reading between 375 and 500 nm (red). A 10 nm slit was used for all the measurements.

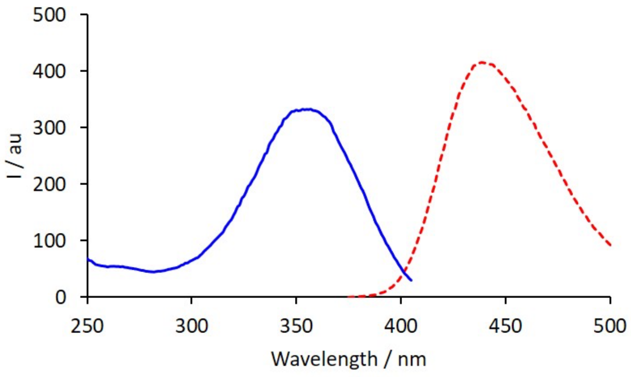

Figure S15. Fluorescence spectra of **1**.

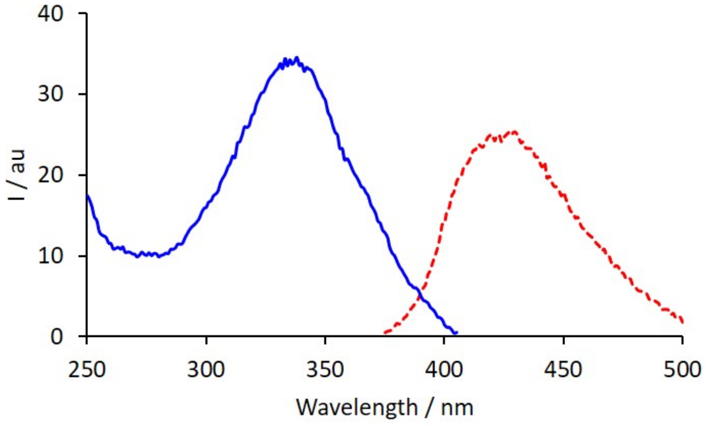

Figure S16. Fluorescence spectra of **14**.

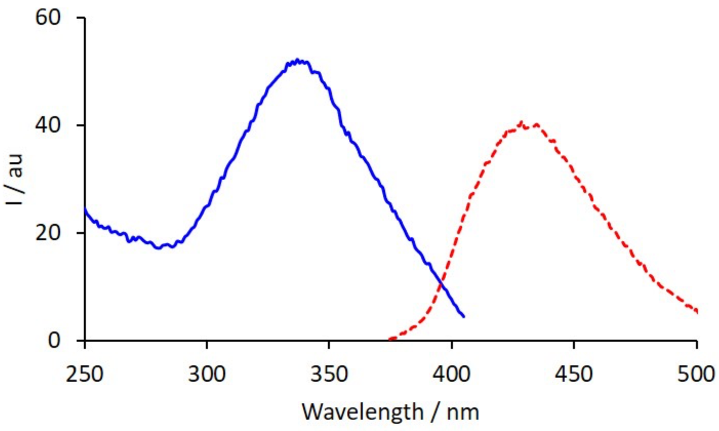

Figure S17. Fluorescence spectra of **15**.

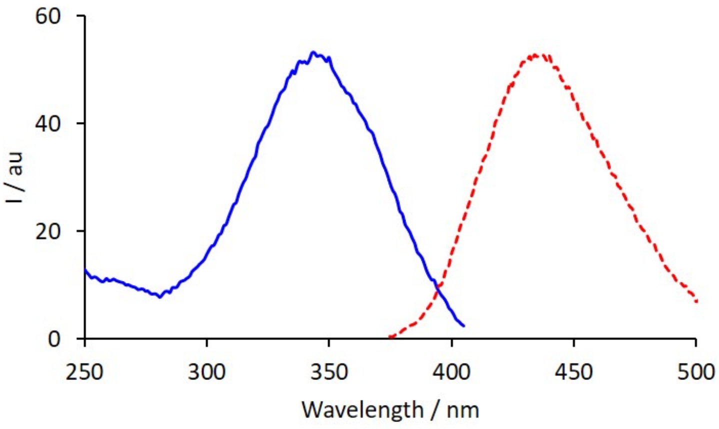

Figure S18. Fluorescence spectra of **16**.

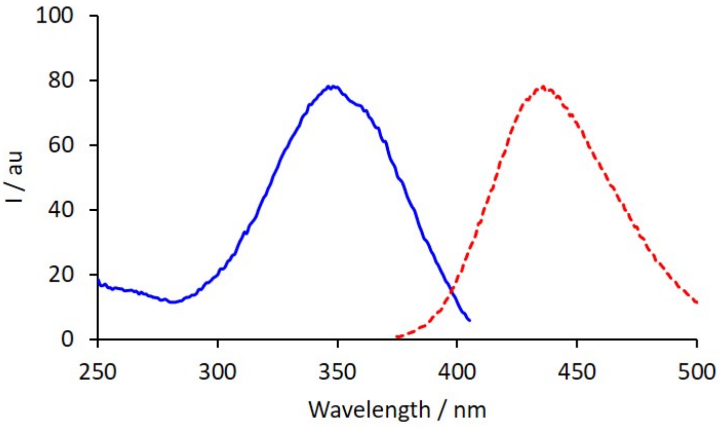

Figure S19. Fluorescence spectra of **17**.

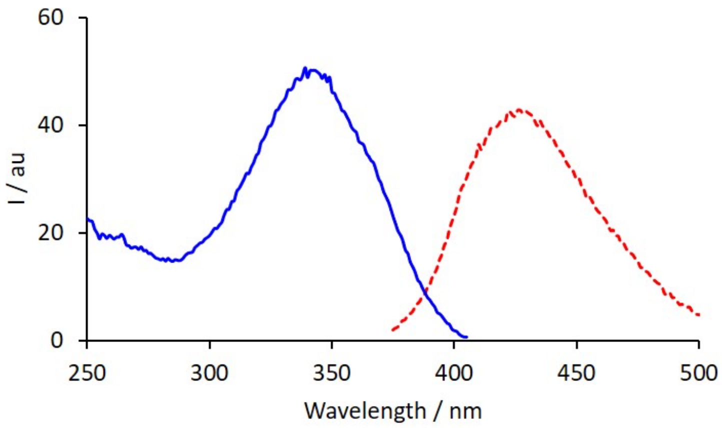

Figure S20. Fluorescence spectra of **24**.

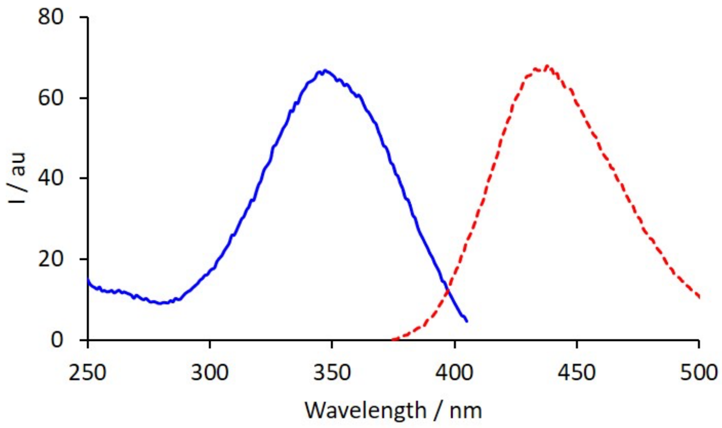

Figure S21. Fluorescence spectra of **25**.

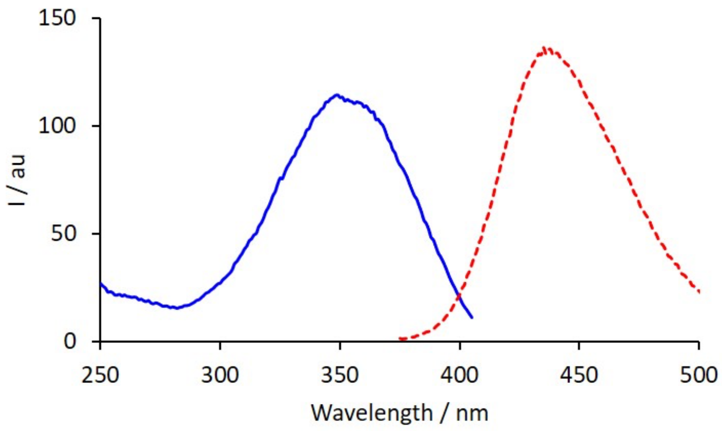

Figure S22. Fluorescence spectra of **26**.

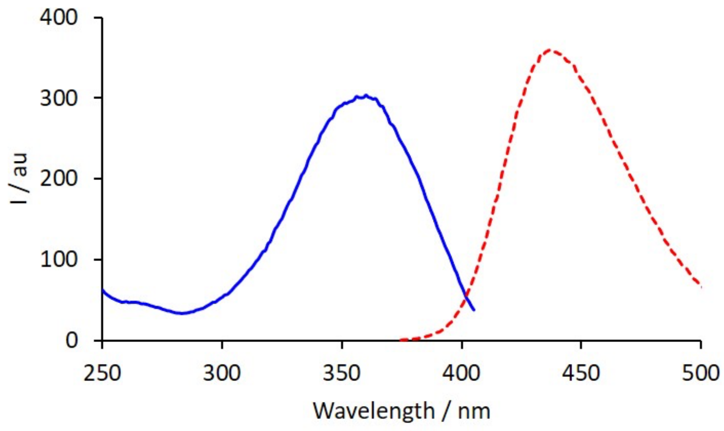

Figure S23. Fluorescence spectra of **27**.

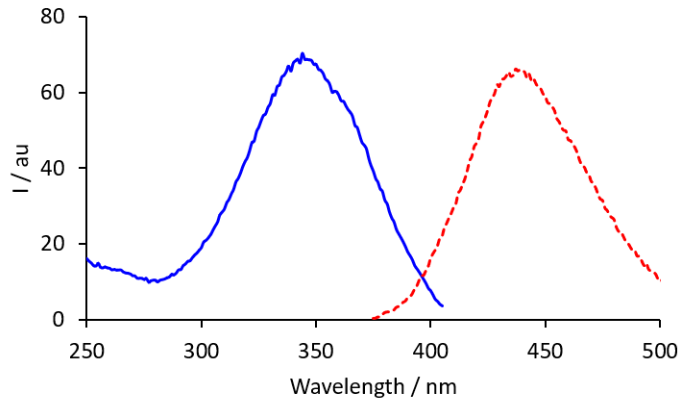

Figure S24. Fluorescence spectra of **29**.

5. Fluorescence titrations

1 mL of stock solutions of the corresponding dyes (1 mM) were prepared in pure DMSO. Stock solutions of protein aggregates, α-synuclein (10 μM), Aβ42 (4 μM) and tau (4 μM), were prepared in PBS 1X buffer. The titration host solutions were prepared by dilution of the corresponding amount of stock solution in PBS 1X, and experiments were carried out at 298 K. The fluorescence spectra were analysed using a Microsoft Excel spreadsheet to fit the changes in fluorescence intensity at fixed wavelengths to a 1:1 binding isotherm by optimizing the association constant, the optical brightness of the free and bound guest, and the concentration of protein binding sites using purpose written VBA macros. All measurements were repeated at least three times, and the errors are quoted at the 95% confidence limit.

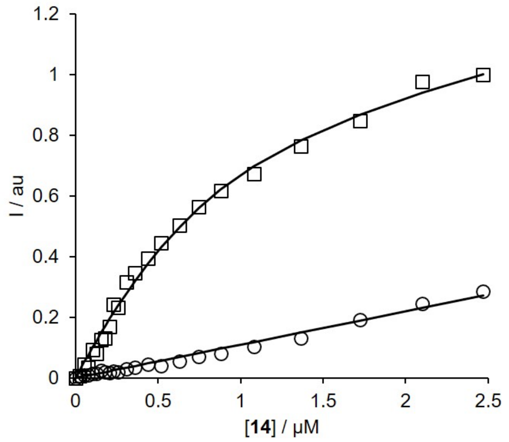

Figure S25. Fluorescence titration of compound **14**. The squares are the data for a titration of a 1 μM solution of α-synuclein solution with increasing amounts of a 5 μM solution of **14**. The spectra were recorded using 355 nm as excitation wavelength and using the emission spectra at 425 nm. The circles are the fluorescence intensity at 425 nm for a titration of the dye into PBS buffer solution. The lines are the fitted data for both processes.

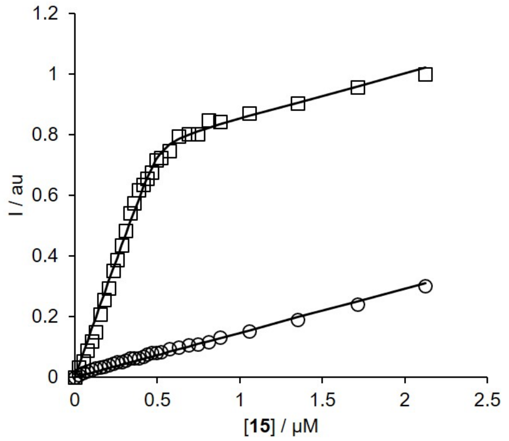

Figure S26. Fluorescence titration of compound **15**. The squares are the data for a titration of a 1 μM solution of α-synuclein solution with increasing amounts of a 20 μM solution of **15**. The spectra were recorded using 355 nm as excitation wavelength and using the emission spectra at 425 nm. The circles are the fluorescence intensity at 425 nm for a titration of the dye into PBS buffer solution. The lines are the fitted data for both processes.

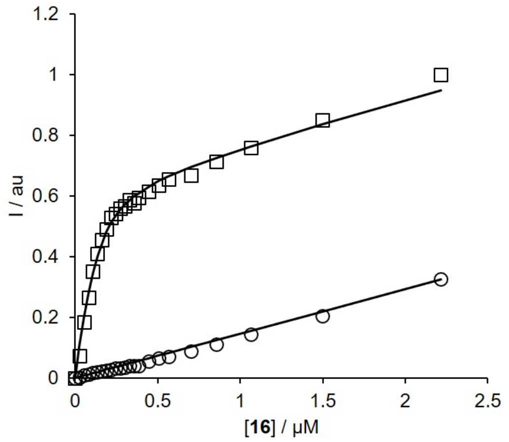

Figure S27. Fluorescence titration of compound **16**. The squares are the data for a titration of a 1 μM solution of α-synuclein solution with increasing amounts of a 5 μM solution of **16**. The spectra were recorded using 355 nm as excitation wavelength and using the emission spectra at 425 nm. The circles are the fluorescence intensity at 425 nm for a titration of the dye into PBS buffer solution. The lines are the fitted data for both processes.

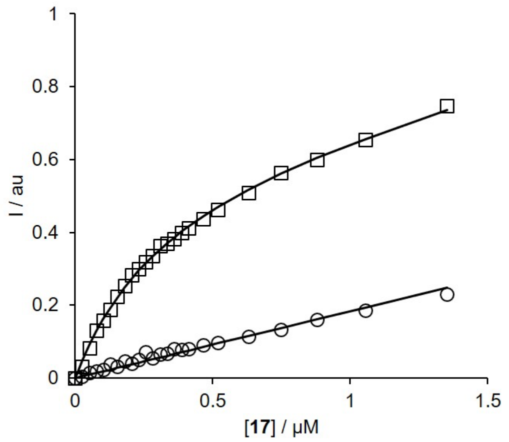

Figure S28. Fluorescence titration of compound **17**. The squares are the data for a titration of a 1 μM solution of α-synuclein solution with increasing amounts of a 5 μM solution of **17**. The spectra were recorded using 355 nm as excitation wavelength and using the emission spectra at 425 nm. The circles are the fluorescence intensity at 425 nm for a titration of the dye into PBS buffer solution. The lines are the fitted data for both processes.

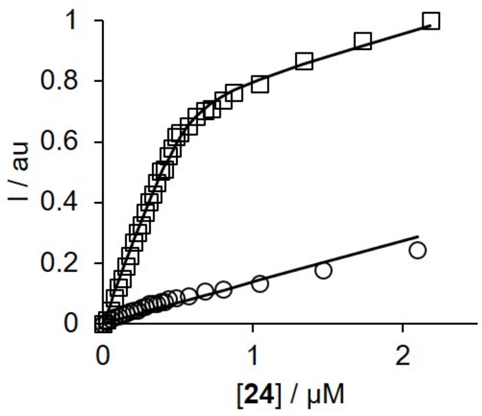

Figure S29. Fluorescence titration of compound **24**. The squares are the data for a titration of a 1 μM solution of α-synuclein solution with increasing amounts of a 5 μM solution of **24**. The spectra were recorded using 355 nm as excitation wavelength and using the emission spectra at 425 nm. The circles are the fluorescence intensity at 425 nm for a titration of the dye into PBS buffer solution. The lines are the fitted data for both processes.

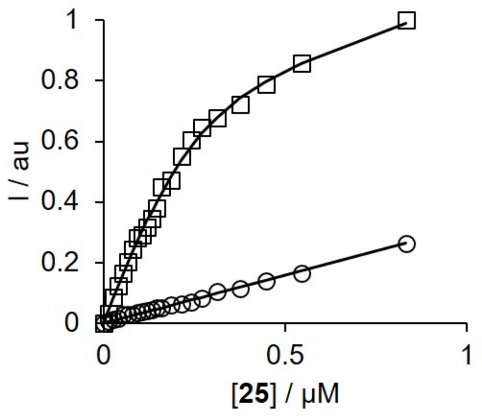

Figure S30. Fluorescence titration of compound **25**. The squares are the data for a titration of a 1 μM solution of α-synuclein solution with increasing amounts of a 2.5 μM solution of **25**. The spectra were recorded using 355 nm as excitation wavelength and using the emission spectra at 425 nm. The circles are the fluorescence intensity at 425 nm for a titration of the dye into PBS buffer solution. The lines are the fitted data for both processes.

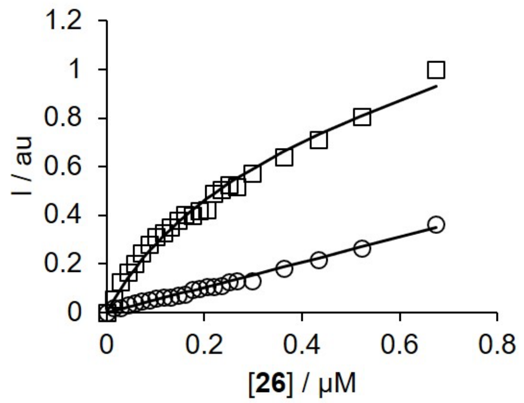

Figure S31. Fluorescence titration of compound **26**. The squares are the data for a titration of a 1 µM solution of α-synuclein solution with increasing amounts of a 2.5 µM solution of **26**. The spectra were recorded using 355 nm as excitation wavelength and using the emission spectra at 425 nm. The circles are the fluorescence intensity at 425 nm for a titration of the dye into PBS buffer solution. The lines are the fitted data for both processes.

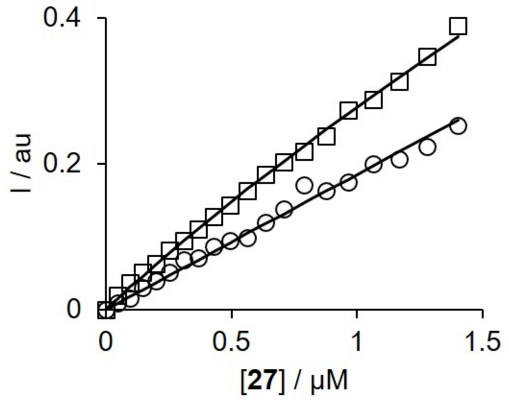

Figure S32. Fluorescence titration of compound **27**. The squares are the data for a titration of a 1 µM solution of α-synuclein solution with increasing amounts of a 7.5 µM solution of **27**. The spectra were recorded using 355 nm as excitation wavelength and using the emission spectra at 425 nm. The circles are the fluorescence intensity at 425 nm for a titration of the dye into PBS buffer solution. The lines are the fitted data for both processes.

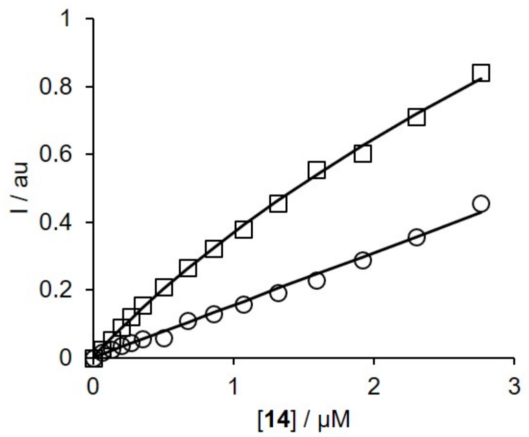

Figure S33. Fluorescence titration of compound **14**. The squares are the data for a titration of a 0.5 μM solution of Aβ_{42} solution with increasing amounts of a 10 μM solution of **14**. The spectra were recorded using 355 nm as excitation wavelength and using the emission spectra at 425 nm. The circles are the fluorescence intensity at 425 nm for a titration of the dye into PBS buffer solution. The lines are the fitted data for both processes.

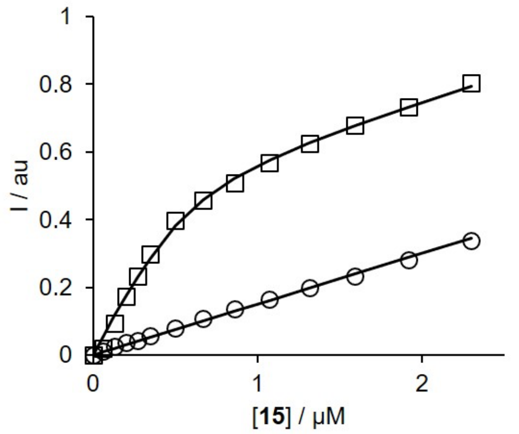

Figure S34. Fluorescence titration of compound **15**. The squares are the data for a titration of a 0.5 μM solution of Aβ_{42} solution with increasing amounts of a 10 μM solution of **15**. The spectra were recorded using 355 nm as excitation wavelength and using the emission spectra at 425 nm. The circles are the fluorescence intensity at 425 nm for a titration of the dye into PBS buffer solution. The lines are the fitted data for both processes.

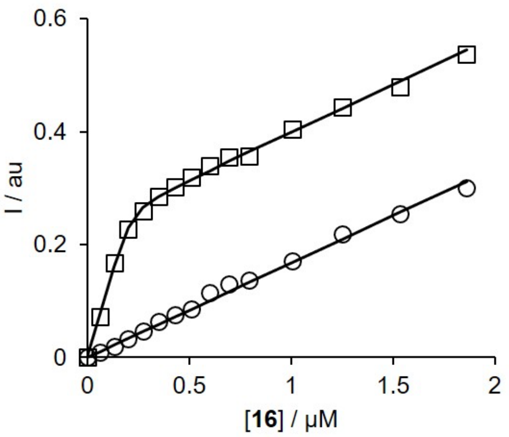

Figure S35. Fluorescence titration of compound **16**. The squares are the data for a titration of a 0.5 μM solution of Aβ_{42} solution with increasing amounts of a 12.5 μM solution of **16**. The spectra were recorded using 355 nm as excitation wavelength and using the emission spectra at 425 nm. The circles are the fluorescence intensity at 425 nm for a titration of the dye into PBS buffer solution. The lines are the fitted data for both processes.

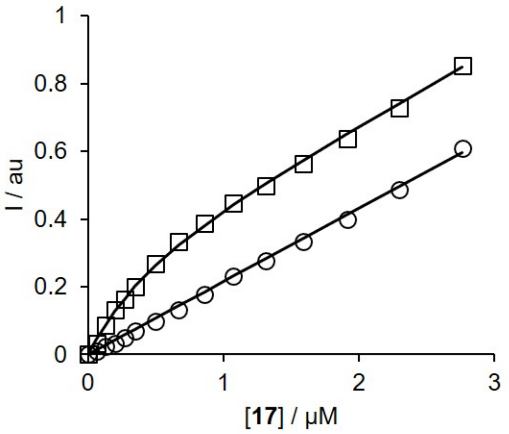

Figure S36. Fluorescence titration of compound **17**. The squares are the data for a titration of a 0.5 μM solution of Aβ_{42} solution with increasing amounts of a 10 μM solution of **17**. The spectra were recorded using 355 nm as excitation wavelength and using the emission spectra at 425 nm. The circles are the fluorescence intensity at 425 nm for a titration of the dye into PBS buffer solution. The lines are the fitted data for both processes.

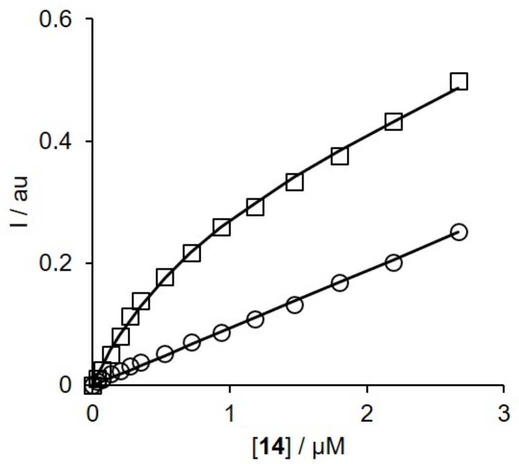

Figure S37. Fluorescence titration of compound **14**. The squares are the data for a titration of a 0.5 µM solution of htau 0N4R solution with increasing amounts of a 12.5 µM solution of **14**. The spectra were recorded using 355 nm as excitation wavelength and using the emission spectra at 425 nm. The circles are the fluorescence intensity at 425 nm for a titration of the dye into PBS buffer solution. The lines are the fitted data for both processes.

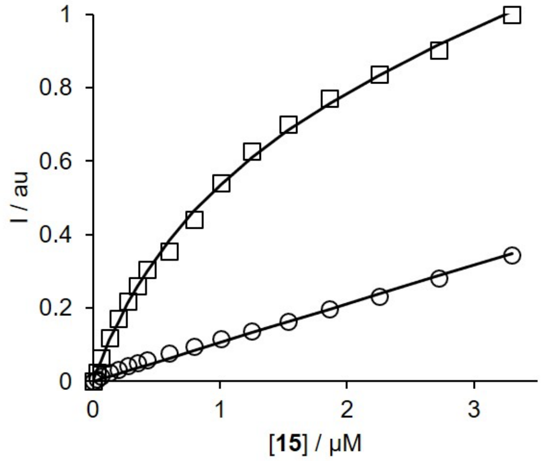

Figure S38. Fluorescence titration of compound **15**. The squares are the data for a titration of a 0.5 µM solution of htau 0N4R solution with increasing amounts of a 12.5 µM solution of **15**. The spectra were recorded using 355 nm as excitation wavelength and using the emission spectra at 425 nm. The circles are the fluorescence intensity at 425 nm for a titration of the dye into PBS buffer solution. The lines are the fitted data for both processes.

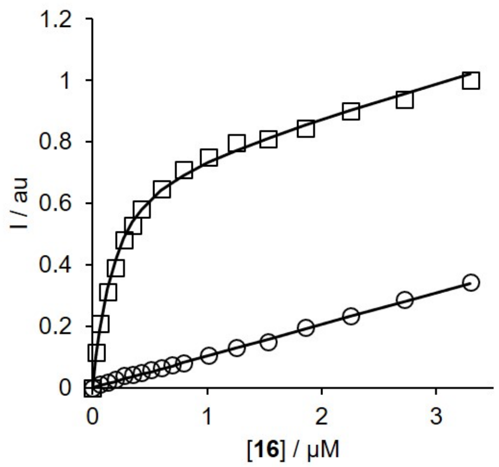

Figure S39. Fluorescence titration of compound **16**. The squares are the data for a titration of a 0.5 μM solution of htau 0N4R solution with increasing amounts of a 12.5 μM solution of **16**. The spectra were recorded using 355 nm as excitation wavelength and using the emission spectra at 425 nm. The circles are the fluorescence intensity at 425 nm for a titration of the dye into PBS buffer solution. The lines are the fitted data for both processes.

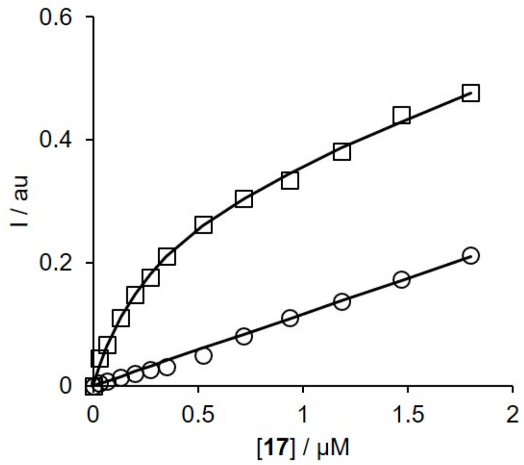

Figure S40. Fluorescence titration of compound **17**. The squares are the data for a titration of a 0.5 μM solution of htau 0N4R solution with increasing amounts of a 12.5 μM solution of **17**. The spectra were recorded using 355 nm as excitation wavelength and using the emission spectra at 425 nm. The circles are the fluorescence intensity at 425 nm for a titration of the dye into PBS buffer solution. The lines are the fitted data for both processes.

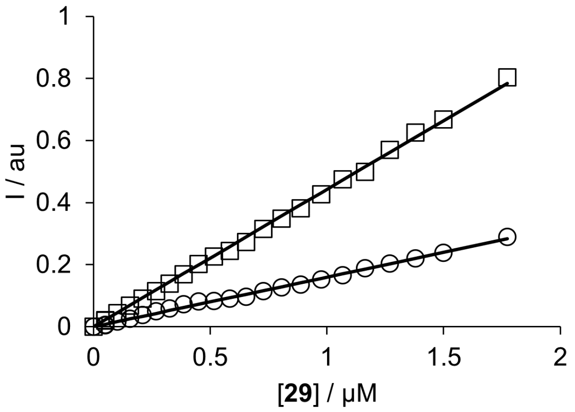

Figure S41. Fluorescence titration of compound **29**. The squares are the data for a titration of a 1 µM solution of α-synuclein solution with increasing amounts of a 10 µM solution of **29**. The spectra were recorded using 355 nm as excitation wavelength and using the emission spectra at 425 nm. The circles are the fluorescence intensity at 425 nm for a titration of the dye into PBS buffer solution. The lines are the fitted data for both processes.

6. Protein aggregation

α-Synuclein protein aggregation was performed following a previously reported procedure.[5] Seed fibrils were produced by incubating a 500 µL solution of monomeric recombinant α-synuclein at 183 µM concentration in PBS buffer at 40 °C for 72 h under stirring with a Teflon stir bar on a heat plate. The fibrils were sonicated between 20 and 30 seconds using a probe sonicator (Bandelin, Sonopuls HD 2070), using 10% maximum power and 50% cycles three times. The sample was then separated into aliquots and stored at -20 °C until required.

Amyloid-β_{1-42} (aβ_{42}) fibrils were obtained by incubating 4 µM of monomeric amyloid-β_{1-42} (Stratech, Catalogue Number: A-1167-2-RPE) in PBS for 4h at 37°C with constant agitation as previously described.[6] The sample was divided into aliquots and kept at - 20 °C until used.

Tau aggregation reactions were performed using 50 µM 0N4R htau (InVivo BioTech Services GmbH, Germany) in buffer containing 100 mM Tris, 150 mM NaCl and 0.1 mM EDTA. The aggregation was initiated by the addition of 0.05 mg/ml heparin (1:4 heparin:tau ratio). The aggregation reaction was incubated at 37°C under quiescent conditions for 120 hours and kept at 4 °C until used.

Nanoscale morphologies of fibril samples were observed by TEM using a Thermo Scientific (FEI Company) Talos F200X G2 microscope operating at 200 kV. Images were recorded with a Ceta 4k x 4k CMOS camera. For sample preparation, TEM grids (continuous carbon film on 300 mesh Cu) were glow discharged using a Quorum Technologies GloQube at 25 mA for 60 s. A 2.5 µL sample of fibril in 1xPBS (4 µM Aβ_{42}, 10 µM αSyn, 50 µM tau with 12.5 µM heparin) was placed on a freshly glow-discharged grid, and after 2 min was carefully removed by blotting with filter paper. The sample was negatively stained using 2.0 µL of aqueous 2% (w/v) uranyl acetate solution for 60 s. The grid was blotted and dried in air for 2 h at room temperature before use.

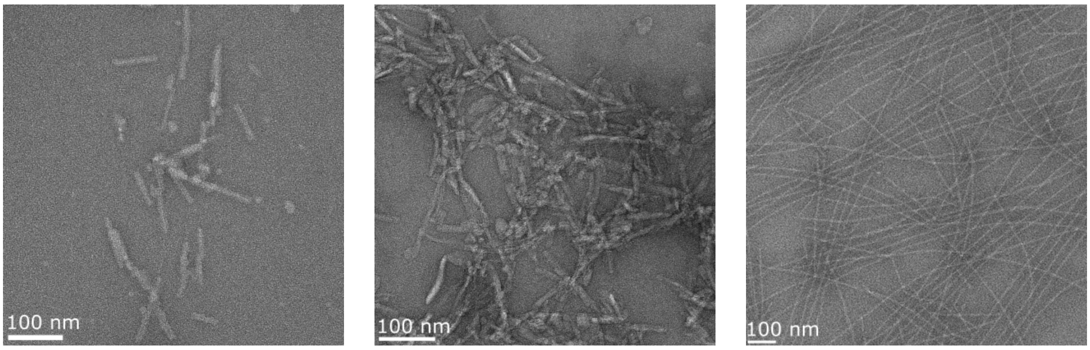

Figure S42. Representative TEM micrograph of protein aggregates: from left to right αSyn, Aβ_{42}, tau.

CD spectra of fibril samples were recorded in 1xPBS (pH 7.4) with a Chirascan CD1 Spectrometer (Applied Photonics Ltd.) equipped with a Series 800 Temperature Controller (Alpha Omega Instruments). Far-ultraviolet measurements (190 nm - 250 nm) were recorded at 25 °C with a 10 mm optical pathlength, a time-per-point of 1.0 s, and a wavelength step of 0.2 nm. Protein concentrations of 1.0 µM for α-Syn, 1.0 µM for tau, and 2.0 µM for Aβ_{42} were used and CD spectra were averaged over six scans. Data were baseline corrected by subtracting the complete buffer spectrum of 1xPBS (pH 7.4) averaged over six scans. Applied Photophysics Pro-Data

Chirascan software was used to convert the data to molar ellipticity, and to smooth the data using Savitsky-Golay smoothing and a window size of five.

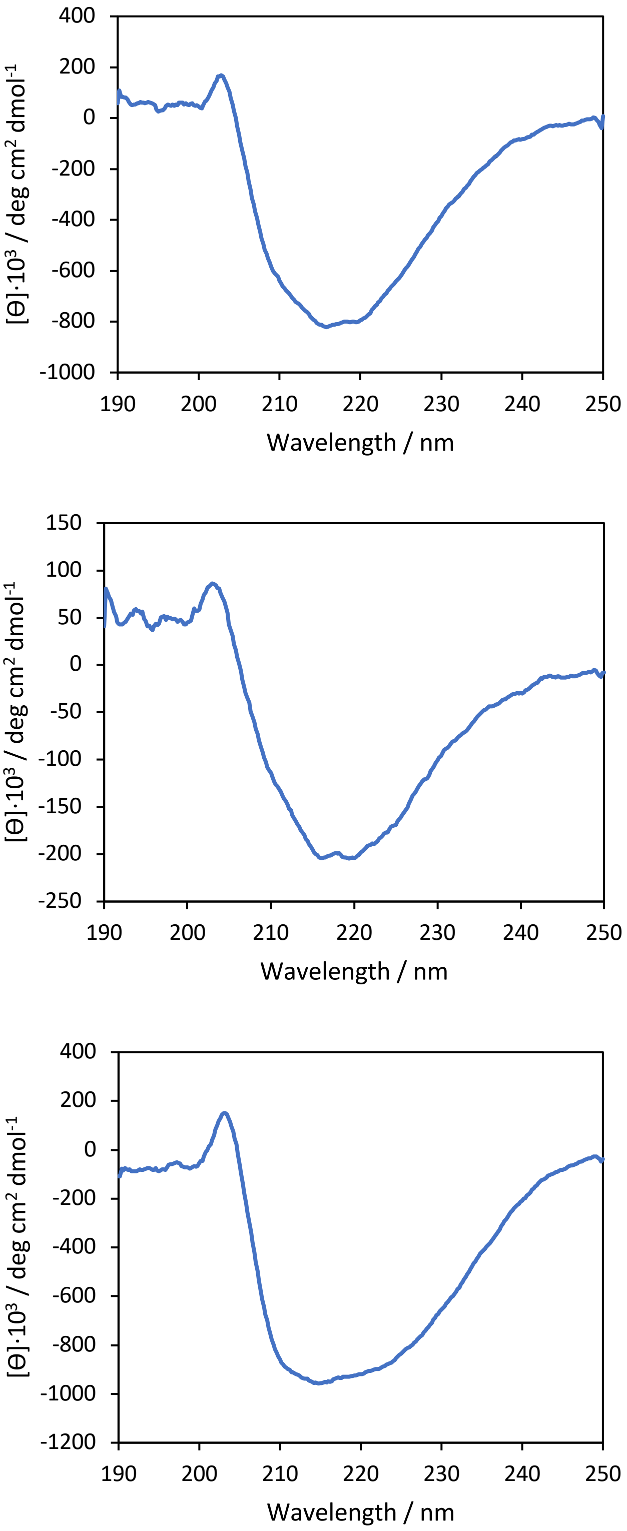

Figure S43. Circular dichroism spectra of protein aggregates in 1xPBS (pH 7.4): from top to bottom αSyn, Aβ_{42}, tau.

7. Total internal reflection fluorescence microscopy (TIRFM) imaging

Imaging was performed using a homebuilt total internal reflection fluorescence microscope as reported previously.[7] Briefly, this imaging mode restricts detectable axial fluorescence signal to within ~200 nm from the glass-water interface. For imaging, the output from laser operating at 405 nm (Oxxius LaserBoxx, product number LBX-405-100-CIR-PP) was aligned and directed parallel to the optical axis at the edge of a 60x Plan Apo TIRF, NA 1.45 oil objective, (Nikon Corporation), mounted an Eclipse TE2000-U microscope (Nikon Corporation) fitted with a Perfect Focus unit. Fluorescence was collected by the same objective and was separated from the returning TIR beam by a dichroic (Di01-R405/488/561/635, Semrock), and passed through appropriate filter (FF01-480/40-25 or FF01-434/17-25 Semrock, for ThT or BTA dimers, respectively). The images were recorded on an EMCCD camera (Evolve 512, Photometrics) operating in frame transfer mode (EMGain of 11.5 e−/ADU and 250 ADU/photon). Each pixel was 241 nm in length. For each data set, 4×4 image grids were measured in at least three different regions of the coverslip. The distance between the nine images measured in each grid was set to 350 µm, and was automated (bean-shell script, Micromanager) to prevent user bias. Images were recorded at 50 ms exposure time for 100 frames with 405 nm illumination (150–200 W/ c2).

Recombinant α-synuclein was diluted in filtered PBS (Whatman Anatop 25 0.02 µm) and mixed with ThT or BTA dimers for a final imaging volume of 50 µL. ThT, dimers and α-synuclein imaging concentration used is indicated for each experiment. All samples were prepared in LoBind microcentrifuge (Eppendorf, Hamburg, Germany) to limit surface adsorption.

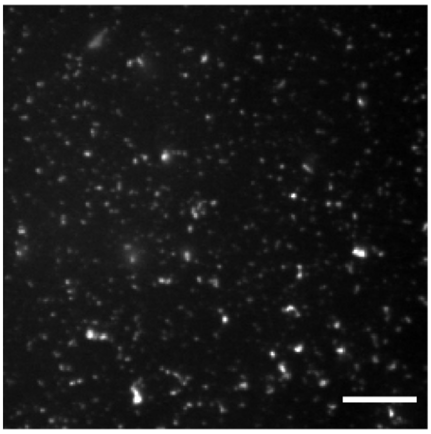

Figure S44. TIRFM imaging of 2.8 µM recombinant α-synuclein with 5 µM ThT. Scale bar 10 µm.

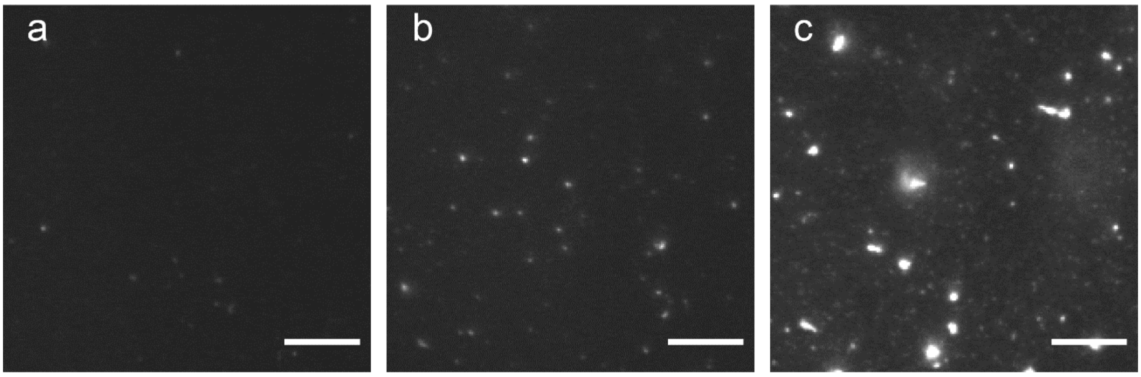

Figure S45. TIRFM imaging of 0.6 µM recombinant α-synuclein fibrils (0.6 µM) with **15** with a) 100 nM, b) 250 nM and, c) 500 nM, using the same contrast. Scale bar 10 µm.

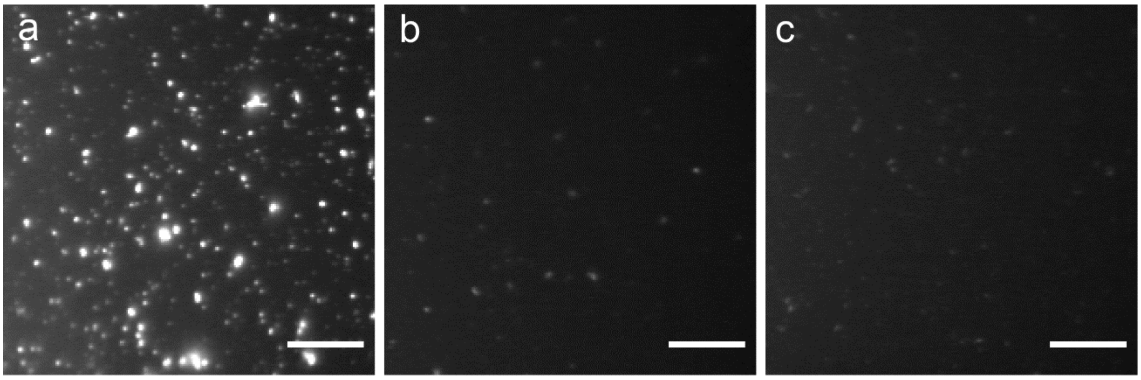

Figure S46. TIRFM imaging of 0.6 µM recombinant α-synuclein fibrils with 0.5 µM of a) **15**, b) **24** and, c) **14** using the same contrast. Scale bar 10 µm.

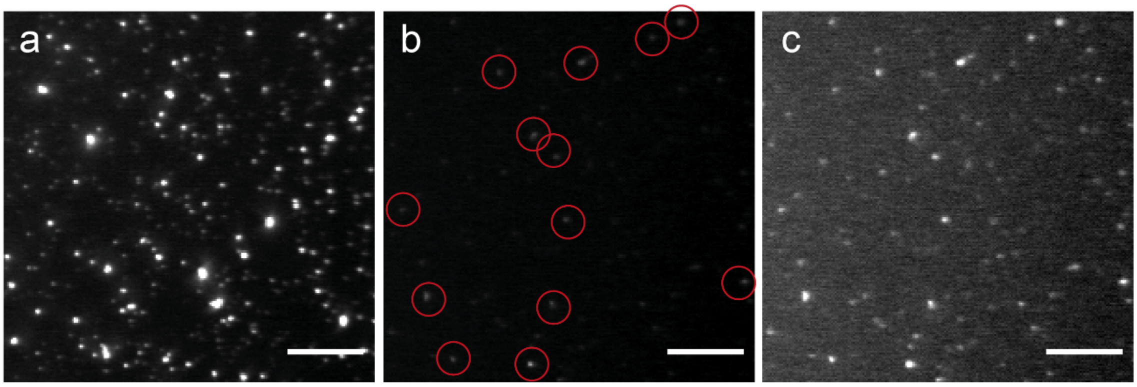

Figure S47. TIRFM images of 200 nM recombinant α-synuclein sonicated fibrils in the presence of a) 200 nM **15** and b) 5 µM ThT. a) and b) have the same contrast and c) is the same as b) but with the contrast adjusted. The red circles in b) highlight the few fluorescent puncta detected when compared with a). Scale bar 10 µm.

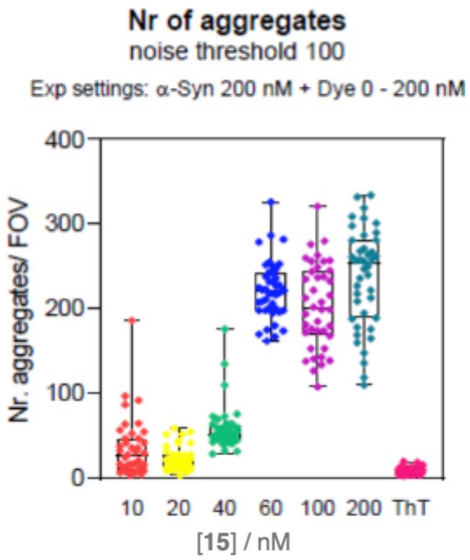

Figure S48. Quantification of number of aggregates in the field of view (FOV) in TIRFM images of α-synuclein sonicated fibrils in the presence of different concentrations of **15** or 5 µM ThT. Fibrils were obtained from 200 nM samples of the monomer.

8. Human Tissue

Frozen brain tissues from neuropathologically confirmed cases of PD and AD were obtained from the Cambridge Brain Bank (Table S3). Tissue blocks were post-fixed in 4% paraformaldehyde and cryoprotected before snap freezing in OCT. Brain sections (15 μm) were generated using a cryostat (Bright OTF5000) for immunohistochemistry and compound staining.

Table S3. Details of human tissue used (SN substantia nigra, FL frontal lobe)

Case no	Diagnosis	Braak Stage	Brain region	Age
**1**	PD	IV	SN	84
**2**	PD	IV	SN	78
**3**	PD	IV	SN	89
**4**	AD	VI	FL	79
**5**	AD	VI	FL	82
**6**	AD	VI	FL	68

9. Immunohistochemistry and Staining

Fixed brain sections were washed with 70% EtOH and allowed to air dry for 20 min before incubation overnight at 4°C with either a mixture of monoclonal antibodies for α-syn (1:500, ab27766, Abcam) and pS129 α-syn (1:500, ab51253, Abcam) for PD sections or a mixture of monoclonal antibody for Aβ (1:500, 800701, Biolegend) and polyclonal antibody for human tau (1:500, A002401-2, Agilent) for AD sections. Sections were washed with PBS 3 x 15 min and incubated for 3 h at room temperature in secondary antibody, either a mixture of goat anti-mouse ALEXA-647 (1:1000, A21235, ThermoFisher) and goat anti-rabbit ALEXA-647 (1:1000, A11011, ThermoFisher) for PD sections or a mixture of goat anti-mouse ALEXA-647 (1:1000, A21235, ThermoFisher) and goat anti-rabbit ALEXA-546 (1:1000, A11035, ThermoFisher) for AD sections. Compounds **13**, **14** and **15** were also diluted in the secondary antibody preparation at stated concentrations. Sections were washed with PBS 3 x 10 min and once with 70% EtOH 5 min before blocking autofluorescence with Autofluorescence Inhibitor (2160, Millipore) for 5 min. After 3 x 1 min washes in 70% EtOH sections were mounted using Fluorosave mounting medium (345789, Millipore). Fluorescence images were taken using a Leica DMI4000B.

10. References

1. a) Qin L., Vastl J., Gao J. *Mol. BioSyst.*, **2010**, *6*, 1791–1795; b) Mathis C. A., Wang Y., Holt D. P., Huang G-F., Debnath M. L., Klunk W. E. *J. Med. Chem.*, **2003**, *46*, 2740–2754.

2. Coferon, INC.; Arnold, Lee Daniel; Foreman, Kenneth, W.; Jin, Meizhong; Wanner, Jutta; Werner, Douglas, S. **Patent:** WO2015/81284 A1, **2015**.

3. a) Krishnamurthy, V. M.; Semetey, V.; Bracher, P. J.; Shen, N.; Whitesides, G. M. *J. Am. Chem. Soc.*, **2007**, *129*, 1312–1320; b) Ahmed, S. A.; Tanaka, M. *J. Org. Chem.*, **2006**, *71*, 9884–9886.

4. a) Krishnamurthy, V. M.; Semetey, V.; Bracher, P. J.; Shen, N.; Whitesides, G. M. *J. Am. Chem. Soc.*, **2007**, *129*, 1312–1320; b) Ahmed, S. A.; Tanaka, M. *J. Org. Chem.*, **2006**, *71*, 9884–9886.

5. Buell, A. K.; Galvagnion, C.; Gaspar, R.; Sparr, E.; Vendruscolo, M., Knowles, T. P. J.; Linse, S.; Dobson, C. M.; *PNAS*, **2014**, *111*, 7671–7676.

6. Flagmeier, P.; De, S.; Wirthensohn, D. C.; Lee, S. F.; Vincke, C.; Muyldermans, S.; Knowles, T. P. J.; Gandhi, S.; Dobson, C. M.; Klenerman, D. *Angew. Chem. Int. Ed.* **2017**, *56*, 7750-7754.

7. Horrocks, M. H.; Lee, S. F.; Gandhi, S.; Magdalinou, N. K.; Chen, S. W.; Devine, M. J.; Tosatto, L.; Kjaergaard, M.; Beckwith, J. S:, Zetterberg, H.; Iljina, M.; Cremades, N.; Dobson, C. M.; Wood, N. W.; Klenerman, D. *ACS Chem. Neurosci.* **2016**, *7*, 399-406.
